# AMPK mediates regulation of glomerular volume and podocyte survival

**DOI:** 10.1172/jci.insight.150004

**Published:** 2021-10-08

**Authors:** Khadija Banu, Qisheng Lin, John M. Basgen, Marina Planoutene, Chengguo Wei, Anand C. Reghuvaran, Xuefei Tian, Hongmei Shi, Felipe Garzon, Aitor Garzia, Nicholas Chun, Arun Cumpelik, Andrew D. Santeusanio, Weijia Zhang, Bhaskar Das, Fadi Salem, Li Li, Shuta Ishibe, Lloyd G. Cantley, Lewis Kaufman, Kevin V. Lemley, Zhaohui Ni, John Cijiang He, Barbara Murphy, Madhav C. Menon

**Affiliations:** 1 Division of Nephrology, Department of Medicine, Icahn School of Medicine at Mount Sinai, New York, New York, USA.; 2 Division of Nephrology, Department of Medicine, Yale University School of Medicine, New Haven, Connecticut, USA.; 3 Department of Nephrology, Renji Hospital, School of Medicine, Shanghai Jiao Tong University, Shanghai, China.; 4 Morphometry and Stereology Laboratory, Charles R. Drew University of Medicine and Science, Los Angeles, California, USA.; 5 Laboratory of RNA Molecular Biology, The Rockefeller University, New York, New York, USA.; 6 Department of Pathology, Icahn School of Medicine at Mount Sinai, New York, New York, USA.; 7 Department of Pediatrics, Children’s Hospital Los Angeles, University of Southern California, Los Angeles, California, USA.

**Keywords:** Cell Biology, Nephrology, Cytoskeleton, Mouse models, Structural biology

## Abstract

Herein, we report that Shroom3 knockdown, via Fyn inhibition, induced albuminuria with foot process effacement (FPE) without focal segmental glomerulosclerosis (FSGS) or podocytopenia. Interestingly, knockdown mice had reduced podocyte volumes. Human minimal change disease (MCD), where podocyte Fyn inactivation was reported, also showed lower glomerular volumes than FSGS. We hypothesized that lower glomerular volume prevented the progression to podocytopenia. To test this hypothesis, we utilized unilateral and 5/6th nephrectomy models in Shroom3-KD mice. Knockdown mice exhibited less glomerular and podocyte hypertrophy after nephrectomy. FYN-knockdown podocytes had similar reductions in podocyte volume, implying that Fyn was downstream of Shroom3. Using SHROOM3 or FYN knockdown, we confirmed reduced podocyte protein content, along with significantly increased phosphorylated AMPK, a negative regulator of anabolism. AMPK activation resulted from increased cytoplasmic redistribution of LKB1 in podocytes. Inhibition of AMPK abolished the reduction in glomerular volume and induced podocytopenia in mice with FPE, suggesting a protective role for AMPK activation. In agreement with this, treatment of glomerular injury models with AMPK activators restricted glomerular volume, podocytopenia, and progression to FSGS. Glomerular transcriptomes from MCD biopsies also showed significant enrichment of Fyn inactivation and Ampk activation versus FSGS glomeruli. In summary, we demonstrated the important role of AMPK in glomerular volume regulation and podocyte survival. Our data suggest that AMPK activation adaptively regulates glomerular volume to prevent podocytopenia in the context of podocyte injury.

## Introduction

Podocytes are specialized epithelial cells on the urinary side of the glomerular filtration barrier, and podocyte actin cytoskeletal disorganization is near universal with nephrotic syndrome (NS) and visualized as foot process effacement (FPE) (1). Focal segmental glomerulosclerosis (FSGS) causes proteinuria and NS, in which podocytes show diffuse FPE associated with podocyte loss, glomerulosclerosis, and progressive renal failure (2); minimal change disease (MCD), in spite of diffuse FPE, shows no podocytopenia and low rates of disease progression. MCD can be morphologically indistinguishable from early FSGS, and some MCD cases reportedly transition to FSGS (2). Hence, comparing signaling events in MCD podocytes during FPE versus FSGS podocytes could define critical mechanisms that maintain podocyte survival in MCD. Using morphometry in humans and experimental models, FSGS has been associated with larger glomerular volumes (Vglom) and podocyte hypertrophy (3–8). By contrast, the specific significance of restricting glomerulomegaly (or Vglom) in MCD is unknown. Signals that promoted an “MCD-like” pathology in the setting of podocyte injury and FPE by restricting glomerulomegaly, preventing podocytopenia and progression to FSGS, would be of considerable therapeutic interest in all NS.

Our group and others have serially reported on the role of Shroom3 in podocytes (9–12). Using an inducible knockdown (KD) model, in young mice (8–12 weeks old), we recently reported that glomerular Shroom3 KD caused proteinuria with diffuse FPE (10). We uncovered a potentially novel protein-protein interaction between Shroom3 and Fyn that regulated Fyn activation. In podocytes in this model, inhibited Nphs1 phosphorylation downstream of Fyn inactivation caused actin cytoskeletal disorganization and FPE. Fyn is a nonreceptor tyrosine kinase and regulates multiple signaling cascades via phosphorylation of tyrosine residues (13). Interestingly, podocyte Fyn inactivation was also specifically associated with human MCD where diffuse FPE without podocytopenia is seen (14, 15). Analogously, in young mice with Fyn inactivation following Shroom3 KD, glomeruli showed no podocytopenia or FSGS in spite of diffuse FPE, an MCD-like pathology. We also observed that podocyte volume was reduced by Shroom3 KD both in vitro and in vivo (10).

Based on this surprising morphometric finding, we first examined a cohort of human NS cases from the Neptune consortium. We identified significantly reduced Vglom in MCD versus FSGS, suggesting maintained Vglom regulation in MCD cases and glomerulomegaly in FSGS. We then performed detailed morphometric studies in our Shroom3-KD model of diffuse FPE, followed by in vitro studies to understand the mechanism and phenotypic studies in the context of podocyte injury, to examine the impact of regulation of Vglom on podocyte survival. These studies revealed enhanced activation of AMPK with either Shroom3 or Fyn KD in podocytes, which accounted for reduced podocyte and Vglom. We confirmed that the mechanism of AMPK activation in podocytes in this model is via cytoplasmic redistribution of the AMPK-activating kinase, LKB1. Because AMPK is a major regulator of cell growth and survival by inhibiting cellular protein synthesis and enhancing autophagy, we postulated that AMPK is a key signaling molecule mediating Vglom regulation and podocyte survival in the context of FPE. Consistent with this, we found that inhibition of AMPK signaling in Shroom3-KD mice with podocyte FPE increased Vglom and induced podocytopenia. Furthermore, activation of AMPK in mice after nephron loss–induced hypertrophic injury restricted Vglom and protected from podocytopenia and progression to FSGS. Finally, evaluation of NS cases within the Nephrotic Syndrome Study Network Consortium (NEPTUNE) showed significant enrichment of signatures of FYN kinase downregulation (and Ampk activation) within MCD glomerular transcriptomes, confirming the translational relevance of our findings.

## Results

### Glomerular morphometry shows significantly lower Vglom in MCD versus FSGS cases.

**Figure 1:**
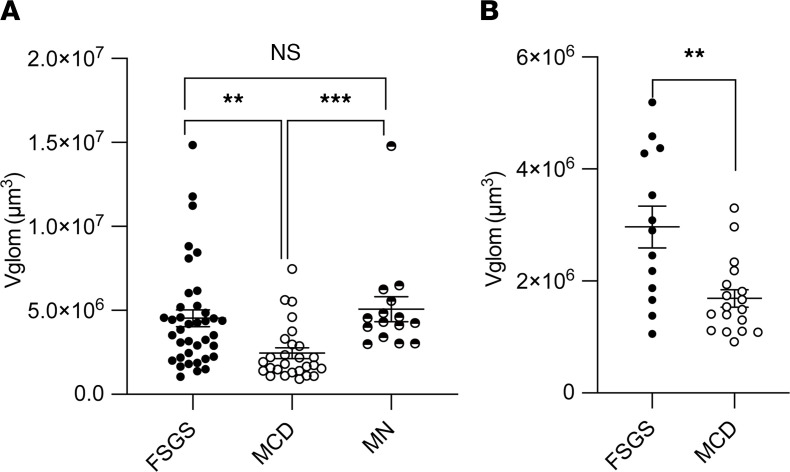
Glomerular morphometry shows significantly lower Vglom in MCD versus FSGS cases. Glomerular morphometry was performed using glomerular area profiles and Weibel-Gomez equation on scanned images of PAS-stained paraffin sections from NS biopsies at enrollment in a subset of the NEPTUNE cohort (*n* = 80 biopsies; 27 MCD, 38 FSGS, 15 membranous nephropathy). Dot plots compare (**A**) mean Vglom (μm^3^) for all FSGS (black solid circles), MCD (black hollow circles), and membranous nephropathy cases (black partly solid circles) in the NEPTUNE morphometry cohort and (**B**) mean Vglom (μm^3^) in pediatric cases (≤18 years) of FSGS (*n* = 13) and MCD (*n* = 18). Line and whiskers indicate mean ± SEM; Kruskal-Wallis test with Tukey’s post test (>2 groups) Mann-Whitney *U* test (2 groups); **P < 0.01, ***P < 0.001; PAS, periodic acid–Schiff.

The Neptune consortium is the largest multicenter prospective cohort of NS collated in the United States with uniform sample/data collection (16). A subset cohort of biopsy-proven NS cases currently has available Aperio-scanned images enabling morphometric evaluation by the NEPTUNE pathology core (5). We investigated Vglom in this subset with annotated diagnoses of FSGS, MCD, or membranous nephropathy (*n* = 80). We excluded the diagnosis of “other NS” from our analyses given the heterogeneity of this entity. The clinical and demographic characteristics of this cohort at enrollment and the outcomes of these patients by diagnosis are shown in Table 1. We applied the Weibel-Gomez formula (adapted from ref. 5 and Methods; see Supplemental Figure 1A; supplemental material available online with this article; https://doi.org/10.1172/jci.insight.150004DS1) on area cross-sections of glomerular tufts identified in the 2 paraffin sections (3–60 glomeruli/patient) to perform Vglom estimation. Mean Vglom (μm^3^) calculated from these 2 random sections within the same biopsy were highly correlated, validating the morphometric assessment (R^2^ = 0.987; *P* < 0.001; Supplemental Figure 1B). We then utilized data from all glomerular profiles from 1 periodic acid–Schiff (PAS) section for mean Vglom estimation and analyses. We found that MCD cases (*n* = 27) had significantly lower mean Vglom than FSGS (*n* = 38) or membranous nephropathy (*n* = 15) (Figure 1A). To minimize confounding of mean Vglom by older age in FSGS cases, we restricted our analyses to pediatric cases alone (≤18 years). Consistently, pediatric MCD (*n* = 18) also had lower mean Vglom than pediatric FSGS (*n* = 13) (Figure 1B). There were no pediatric membranous nephropathy cases. Furthermore, in Cox proportional hazard models agnostic to diagnoses, increasing Vglom was associated with increased composite risk of end-stage renal disease and/or 40% or greater decline in estimated glomerular filtration rate (eGFR) during follow-up, independent of age (HR = 1.18 per 10^6^ μm^3^; Table 2). The covariates included in these models were those identified as significantly different between the 3 NS diagnoses in univariable analyses (Table 1). Hence, these data identified lower Vglom in MCD cases versus FSGS, representing maintained Vglom regulation in MCD (and dysregulation in FSGS), and the data showed an association of higher Vglom with adverse renal outcomes in NS.

### Global or podocyte-specific Shroom3 KD reduced glomerular and podocyte volume.

**Figure 2:**
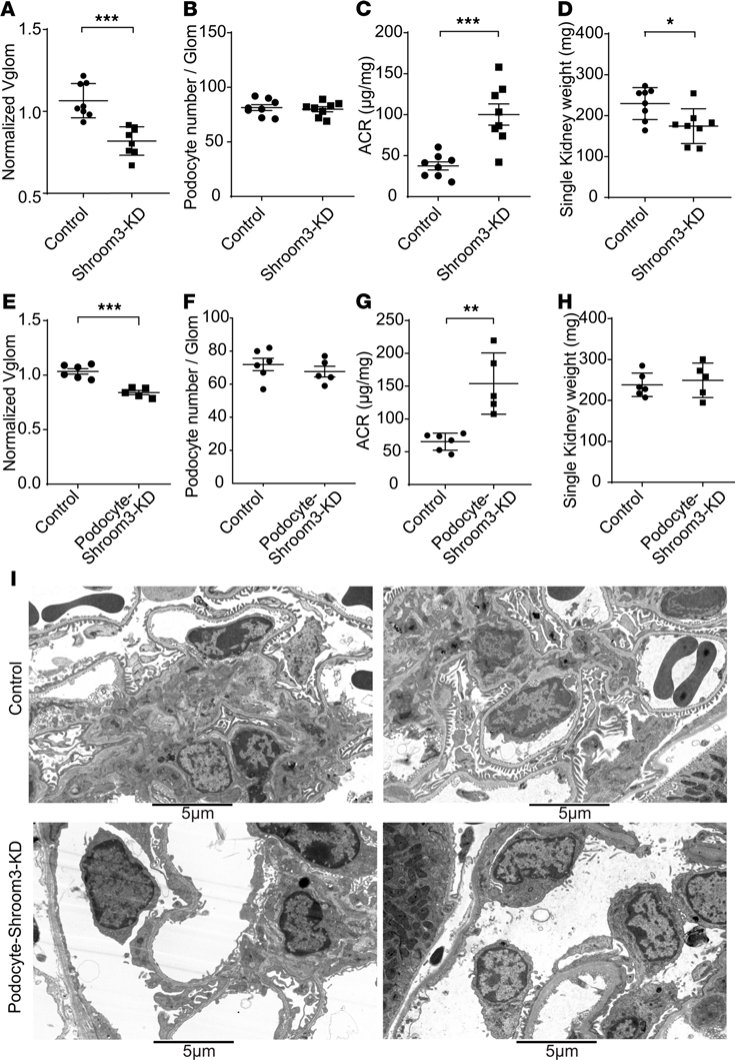
Global or podocyte-specific Shroom3 knockdown reduced glomerular and podocyte volume. (**A–D**) Global Shroom3-KD and control mice (~12 weeks) were DOX fed for 6 weeks (*n* = 8 each). Kidney tissues were embedded in plastic and 1 μm sections stained with Toluidine blue. Dot plots compare (**A**) mean Vglom (×1000 μm^3^) by Cavalieri method (10 glomeruli/mouse), (**B**) mean podocyte numbers per glomerulus per animal (fractionator-disector method), (**C**) ACR (mcg/mg) at 6 weeks, and (**D**) single kidney weights at euthanization (mg) in knockdown versus control groups. (**E–I**) To induce podocyte-specific Shroom3-KD mice and controls (~12 weeks old) were DOX fed for 6 weeks (*n* = 6 vs. 5). Dot plots compare (**E**) mean Vglom (×1000 μm^3^) by Cavalieri method (10 glomeruli/mouse), (**F**) mean podocyte numbers per glomerulus per animal (fractionator-disector method), (**G**) ACR (mcg/mg) at 6 weeks, and (**H**) single kidney weights at euthanization (mg) in podocyte-specific knockdown versus control groups. (**I**) Panel shows electron microscopic images of representative podocyte-specific Shroom3-KD mice and controls (*n* = 2 each) showing podocyte FPE in knockdown glomeruli. ACR, albumin/creatinine ratio. Line and whiskers indicate mean ± SEM; unpaired *t* test; *P < 0.05, **P < 0.01, ***P < 0.001.

Our human data provided rationale to comprehensively evaluate glomerular morphometry in our inducible *Shroom3*-KD mice, where we had observed diffuse FPE without podocytopenia, along with reduced podocyte volume. In this mouse model (Shroom3-KD mice), universal shRNA-mediated Shroom3 KD and turbo GFP (tGFP) production were induced in all tissues by doxycycline feeding (DOX; see Supplemental Methods) (9, 10, 17). Nontransgenic or monotransgenic littermates were used as controls. As we previously reported, global Shroom3-KD mice develop diffuse podocyte FPE by 6 weeks of DOX (10). We first evaluated Vglom using the Cavalieri principle (Supplemental Figure 2, A and B) after inducing *Shroom3* KD. Here, we identified significant Vglom reductions in Shroom3-KD versus controls (Figure 2A and Supplemental Table 1A; *n* = 8; mean difference in Vglom ~23%). As described previously (10, 18), we also estimated the volume of glomerular components, i.e., podocytes (PodoVglom), capillary lumens plus endothelium (Cap+EndoVglom), and mesangial components (see Methods and Supplemental Figure 2, C and D). Glomerular component volume analysis revealed reductions in PodoVglom (*P* < 0.05) and Cap+EndoVglom (*P* = 0.06) in Shroom3-KD glomeruli versus controls (Supplemental Table 1A). No podocytopenia was identifiable at 6 weeks DOX in Shroom3-KD mice (Figure 2B); indeed, podocyte numerical density (podocytes per unit Vglom expressed as n/μm^3^) was higher in Shroom3-KD glomeruli (Supplemental Figure 2E). As previously described, Shroom3 KD induced increased albuminuria (Figure 2C) without azotemia (Supplemental Figure 2F). We also observed significantly lower single kidney weights in Shroom3-KD mice, while body weights remained similar to controls (Figure 2D and Supplemental Figure 2G, respectively). The mean difference in kidney weights was 24%, similar to Vglom changes, and suggested the involvement of nonglomerular kidney cells in the renal phenotype observed with global Shroom3 KD. We evaluated nephron endowment in control and Shroom3-KD mice using nephron density (Vglom density and number density by morphometry), which was similar in the 2 groups and did not explain changes in Vglom (controls vs. Shroom3-KD = 189 ± 50.7 vs. 174 ± 44.8 per mm^3^ of cortex; *P* = 0.5; Supplemental Figure 2, H and I).

To test the hypothesis that podocyte Shroom3 regulated Vglom, we crossed Nphs1-rtTA (19) mice with our inducible Shroom3-shRNA mice (9, 10) for podocyte-specific *Shroom3* KD (Podocyte-Shroom3-KD mice). Glomerular protein extracts showed Shroom3 KD and tGFP production in Podocyte-Shroom3-KD mice (Supplemental Figure 2J), and quantitative (qPCR) analysis confirmed *Shroom3* KD in Podocyte-Shroom3-KD glomeruli (Supplemental Figure 2K). Podocyte-Shroom3-KD mice with 6 weeks of DOX similarly demonstrated significantly reduced mean Vglom (mean difference ~15%) as well as reduced PodoVglom and Cap+EndoVglom (Figure 2E and Supplemental Table 1B) versus controls (*n* = 6 vs. 5). No podocytopenia was identifiable by the fractionator-disector method (Figure 2F). Podocyte Shroom3-KD mice also showed increased albuminuria without azotemia (Figure 2G and Supplemental Figure 2L). Neither body weights (Supplemental Figure 2M) nor single kidney weights (Figure 2H) were significantly different between Podocyte-Shroom3-KD and control mice, suggesting minimal effect on nonglomerular cells due to podocyte-specific Shroom3 KD. Electron microscopy examination revealed podocyte FPE (Figure 2I), similar to global Shroom3-KD animals (10). Quantification of foot process width (FPW) consistently revealed higher FPW among representative Podocyte-Shroom3-KD mice versus controls (Supplemental Figure 2N). These data suggested that, in addition to inducing albuminuria with FPE, global or podocyte-specific Shroom3 KD reduced Vglom in adult mice without podocytopenia.

### Shroom3 KD restricted glomerular hypertrophy after unilateral nephrectomy.

**Figure 3:**
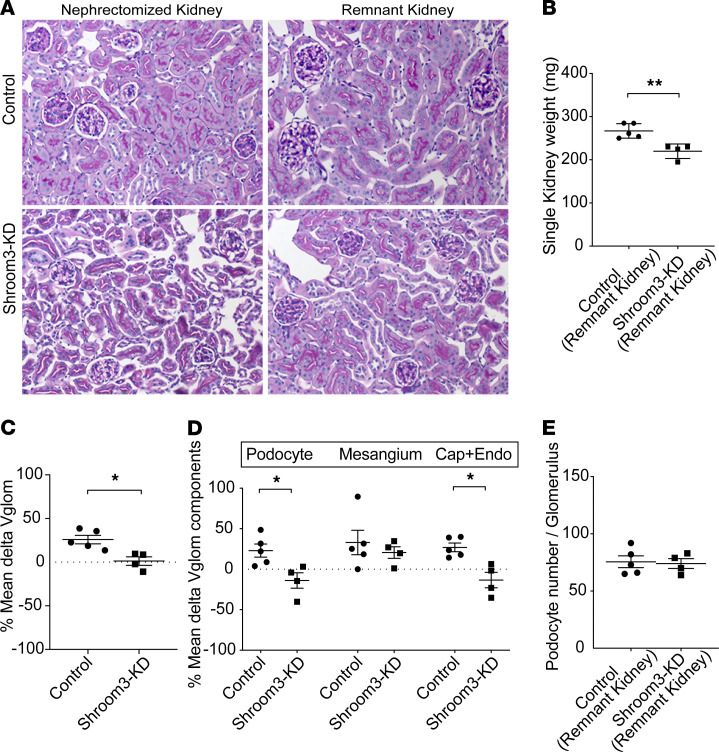
Shroom3 knockdown restricted glomerular hypertrophy after unilateral nephrectomy. Global Shroom3-KD and control mice (~12 weeks) were DOX fed for 6 weeks and then subjected to unilateral nephrectomy (*n* = 5 vs. 4). (**A**) Panel shows respective representative PAS-stained images (20×) of nephrectomized and remnant kidneys (at day 7 after nephrectomy) of knockdown and control mice. (**B**) Dot plots compare mean kidney weight of the remnant kidney (mg) in knockdown versus control groups. (**C**) Percentage change (or Δ) of mean Vglom and (**D**) Vglom components (podocyte, mesangial, and capillary + endothelium [Cap+Endo] components) of nephrectomized and remnant kidneys for each animal are shown in dot plots. (**E**) Dot plots show mean podocyte numbers per glomerulus of remnant kidney per animal in each group (fractionator-disector method, 10 glomeruli/animal). Line and whiskers indicate mean ± SEM; unpaired *t* test; *P < 0.05, **P < 0.01.

Because morphometric data from podocyte-specific Shroom3-KD phenocopied global Shroom3-KD, we used global Shroom3-KD mice, which were backcrossed into a susceptible BALB/c background for further experiments. To further examine Vglom regulation by Shroom3, we performed unilateral nephrectomy in Shroom3-KD and control mice as described (8) and evaluated glomerular hypertrophy using nephrectomized and remnant kidneys (Figure 3A) at 1 week after nephrectomy (*n* = 4 vs. 5, respectively). First, after unilateral nephrectomy, the mean weight of the remnant kidney was reduced in global Shroom3-KD versus control animals (Figure 3B). By morphometry, the percentage change in mean Vglom after nephrectomy was restricted in Shroom3-KD but not in control mice (Figure 3C). As described previously (Supplemental Figure 3, A and B) (10, 18), nephrectomized and remnant kidneys were used to evaluate the postnephrectomy expansion of glomerular components. Among glomerular components, PodoVglom expansion and Cap+EndoVglom expansion were significantly restricted (Figure 3D) in Shroom3-KD remnant kidneys versus controls. No podocytopenia was identifiable in remnant kidneys in either Shroom3-KD or control groups at 1 week after nephrectomy (fractionator/disector method [Figure 3E] or Wilms’ tumor 1 (WT1) immunofluorescence [data not shown]). After nephrectomy, Shroom3-KD mice had significantly increased albuminuria but no azotemia versus controls (Supplemental Figure 3, A and B). These data showed that remnant kidneys in Shroom3-KD mice showed restricted Vglom expansion without podocytopenia and further demonstrated the regulation of Vglom by Shroom3 KD.

### Shroom3 KD reduces cellular protein content and RNA biogenesis in vitro and in vivo mediated via FYN.

**Figure 4:**
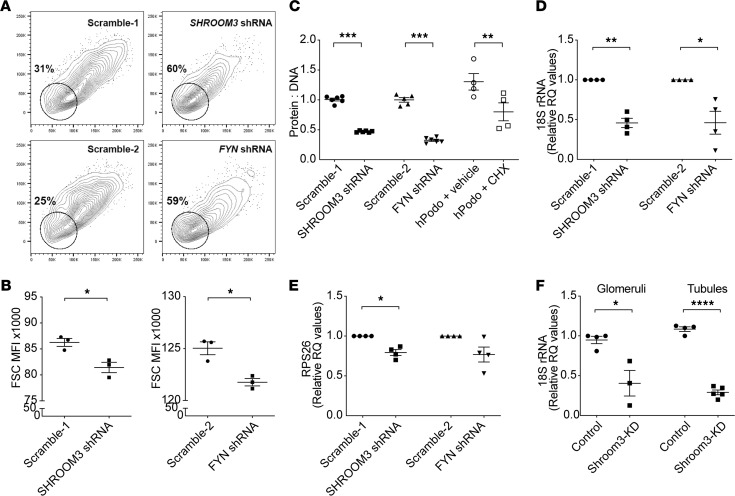
Shroom3 knockdown reduces cellular protein content and RNA biogenesis in vitro and in vivo mediated via FYN. Puromycin-selectable, stable Shroom3 and Fyn knockdown podocytes were generated using lentiviral shRNA infection (Scramble-1 and Scramble-2 are respective scramble-sequence infected controls). Stable podocytes were differentiated (>7 days) in collagen-coated plates. (**A**) Representative panels show forward scatter (FSC) MFI of Scramble-1, Scramble-2, Shroom3-, and FYN-shRNA podocytes; (**B**) shows corresponding dot plots. Arbitrary gate (circle) shows compact clustering of knockdown cells versus scramble (*n* = 3 sets each). (**C**) Dot plots compare protein/DNA ratios of Scramble-1, Scramble-2, Shroom3-, and FYN-shRNA podocytes. Cycloheximide (CHX) was used as a positive control to inhibit protein synthesis. Dot plots show mean fold-changes of (**D**) 18S rRNA copies and (**E**) ribosomal protein S26 (RPS26) transcripts in Scramble-1, Scramble-2, Shroom3-, and FYN-shRNA (normalized to actin; *n* = 4 sets); (**F**) compares 18S rRNA copies in glomerular and tubular fractions of control versus Shroom3-KD mice. Line and whiskers indicate mean ± SEM; unpaired *t* test; *P < 0.05, **P < 0.01, ***P < 0.001, ****P < 0.0001; **D** and **E** compared by paired *t* test; RQ, relative quantity.

Since we reported that Shroom3 KD reduced podocyte volume and inactivated FYN in podocytes (10), we first examined whether the regulation of cell volume by Shroom3 was mediated via FYN. We generated a FYN KD stable podocyte line using lentivirally transduced shRNA. *FYN*-shRNA podocytes had significantly reduced cell volume by flow cytometric forward scatter versus corresponding Scramble-2 podocytes and similar volume as Shroom3-shRNA podocytes (10) (Figure 4, A and B; *n* = 3 sets). This suggested that FYN was downstream of the regulation of podocyte volume by Shroom3.

To examine whether Shroom3/Fyn KD in podocytes reduced cell size by reducing cellular protein content, we performed protein/DNA ratio estimation in Shroom3/Fyn-KD podocytes versus scramble controls. We used cycloheximide (20), a protein synthesis inhibitor, as a positive control. We identified markedly reduced protein/DNA ratio with Shroom3 or Fyn KD (Figure 4C). Next, we examined RNA biogenesis by quantifying ribosomal RNA copies, including 18S, 5S, and RPS26 in KD cells (normalized to actin). We observed significantly reduced 18SrRNA copies in vitro in Shroom3 or FYN-KD podocytes (Figure 4D). *RPS26* transcripts were also reduced in SHROOM3-shRNA podocytes (Figure 4E). In vivo in both glomerular and tubular fractions, 18S rRNA copies were significantly reduced in Shroom3-KD kidneys versus controls (Figure 4F), also suggesting inhibited protein synthesis in nonglomerular kidney cells in global Shroom3-KD animals. Interestingly, 5S rRNA transcripts were unchanged in in vitro Shroom3/Fyn-KD and in vivo Shroom3-KD versus control animals (Supplemental Figure 4, A and B).

### Shroom3 or Fyn KD increases cellular AMPK activation.

**Figure 5:**
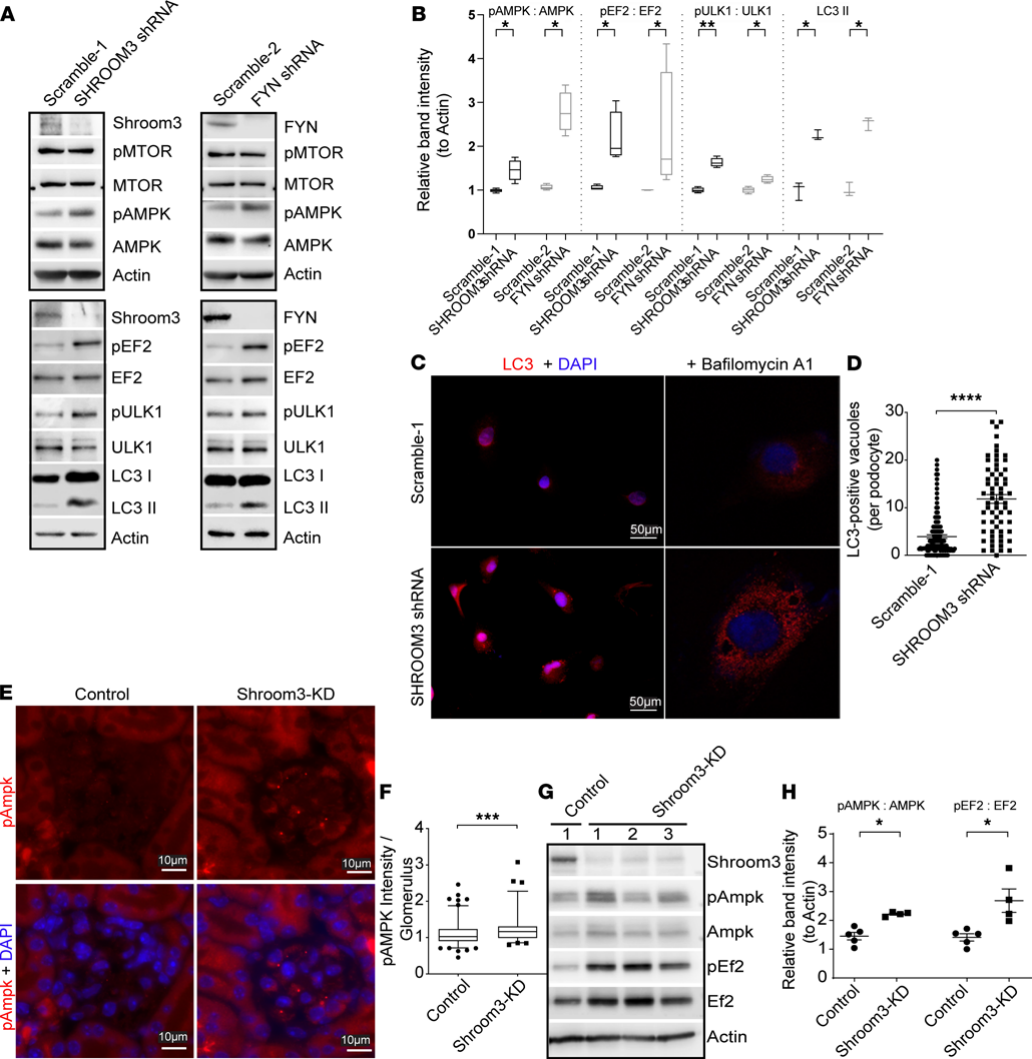
Shroom3 or Fyn knockdown increases cellular AMPK activation. (**A**) WBs of lysates from Scramble-1, *SHROOM3*-shRNA (top panel), and Scramble-2 *FYN*-shRNA podocytes (top panel) probed for SHROOM3 or FYN and phosphorylated and total MTOR, phosphorylated and total AMPK, and actin. Representative WBs of lysates from Scramble-1, SHROOM3-shRNA (bottom panel), and Scramble-2 FYN-shRNA podocytes (bottom panel) probed for SHROOM3 or FYN, phosphorylated and total EF2, phosphorylated and total ULK1, LC3-I and II, and actin. (**B**) Box plots show respective relative band intensity (normalized to actin) (*n* ≥ 3 sets). (**C**) Representative immunofluorescence images (left panels = 20×) of *SHROOM3*-shRNA and Scramble-1 cells stained for LC3 (TRITC). Right panels show confocal images obtained after 24 hours of bafilomycin treatment. (**D**) Dot plots quantify LC3-positive vacuoles per podocyte (*n* = 2 sets). (**E**) Representative immunofluorescence images show glomerular staining for phosphorylated AMPK (top row) and merge with DAPI (bottom row) in Shroom3-KD and control mice. (**F**) Box plots quantify intensity of phosphorylated AMPK/per glomerular outline per group (30 glomeruli/animal; *n* = 5 each). (**G**) Representative WBs of glomerular lysates from Shroom3-KD mice and controls probed for Shroom3, phosphorylated and total Ampk, phosphorylated and total Ef2 and actin, and (**H**) respective relative band intensities are shown (normalized to actin; *n* > 4 each). WB, Western blot. In the box-and-whisker plot, the box represents the middle quartiles, the lines indicate the median, and the whiskers denote the 5th–9th percentile. Line and whiskers indicate mean ± SEM; unpaired *t* test; *P < 0.05, **P < 0.01, ***P < 0.001, ****P < 0.0001.

Since cellular size and protein biosynthesis were reduced with Shroom3-Fyn KD and previous data from Fyn-knockout mice showed increased activation of AMPK (21), a negative regulator of cellular protein biosynthesis, we examined AMPK signaling after Shroom3 or Fyn KD in podocytes. We identified significantly increased AMPK phosphorylation at threonine-172 (or pAMPK) in both *SHROOM3*- and *FYN*-shRNA–transduced podocytes versus respective scramble controls (Figure 5, A and B; *n* = 4 sets). Cellular AMPK activation is stereotypically induced by increased AMP/ATP ratio and is a negative regulator of protein synthesis (22, 23). Consistent with this, phosphorylated EF2/total EF2 ratio downstream of AMPK was enhanced in KD podocytes versus controls, suggesting inhibited protein translation (Figure 5, A and B). Phosphorylation of MTOR was, however, not significantly different in scramble versus KD lines. Increased levels of phosphorylated ULK1 and LC3II (downstream of pAMPK) were also identified in KD podocytes (Figure 5, A and B). We identified increased LC3-positive vacuoles *in SHROOM3*-shRNA podocytes versus controls; bafilomycin (24) treatment further accentuated LC3-positive vacuoles in *SHROOM3*-shRNA cells, confirming significantly increased autophagic flux (Figure 5, C and D). In agreement, increased pAMPK staining was seen in glomeruli of Shroom3-KD versus control mice (*n* = 4 vs. 5 mice; Figure 5, E and F; and Supplemental Figure 5A). Glomerular lysates from Shroom3-KD/control animals confirmed increased pAmpk and phospho-Ef2 in Shroom3-KD mice (Figure 5, G and H). We also examined whole kidney lysates and tubular extracts of Shroom3-KD/control animals (*n* = 4 each) and confirmed increased Lc3-II in Shroom3-KD mice (Supplemental Figure 5, B–D), suggesting extension of Ampk-activation to nonglomerular cells with global Shroom3 KD. Together, these data demonstrated increased cellular AMPK activation after Shroom3 or FYN KD with reduced protein synthesis and increased autophagy, leading to reduced cellular protein content.

We previously demonstrated that Shroom3 KD led to Fyn inactivation due to loss of Shroom3-Fyn interaction between the respective SH3-binding and SH3 domains (10). Additionally, cells with FYN deletion or inactivation showed increased pAMPK via increased LKB1 cytoplasmic distribution (21). LKB1 phosphorylates Thr-172 of the kinase subunit of AMPK and is ubiquitous. We therefore examined LKB1 localization in *SHROOM3*-shRNA podocytes. Nuclear, cytoplasmic, and membrane protein extracts after subcellular fractionation showed a consistently reduced nuclear pool of LKB1 and increased LKB1 cytoplasmic/nuclear ratio, suggesting LKB1 redistribution to the cytoplasm in *SHROOM3-*shRNA podocytes versus scramble (Supplemental Figure 5, E and F). Consistent with AMPK activation, phosphorylated-EF2 was also increased in *SHROOM3*-shRNA podocytes in subcellular fractions (Supplemental Figure 5E). In summary, after Shroom3 KD in podocytes, Fyn inactivation led to LKB1 redistribution to the cytoplasm and consequent AMPK activation (Supplemental Figure 5G).

### AMPK activation reduces Vglom and mitigates podocytopenia in aged Shroom3-KD mice with podocyte FPE.

**Figure 6:**
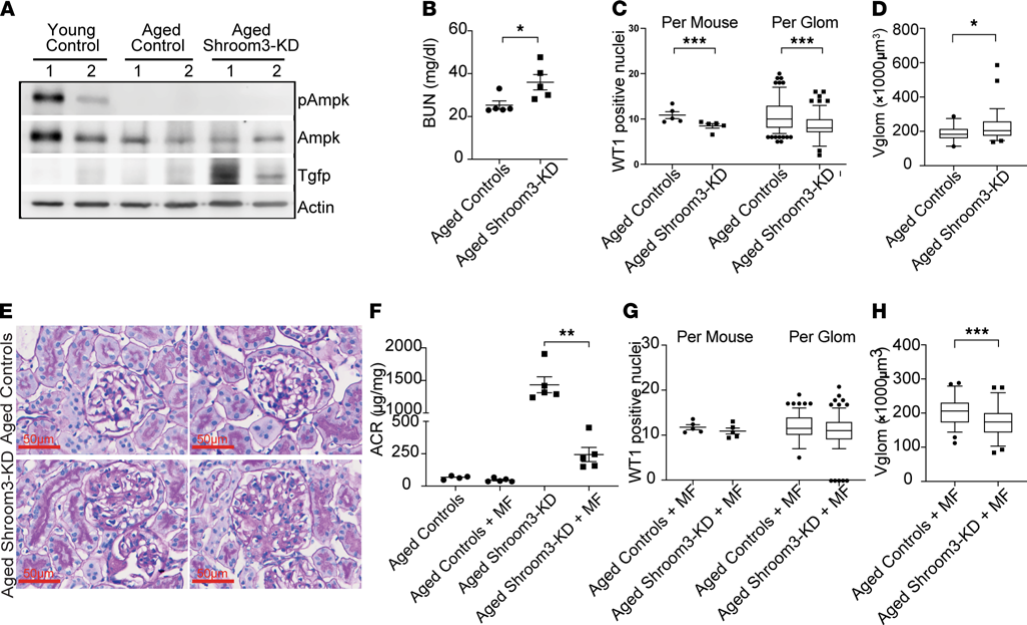
AMPK activation reduces Vglom and mitigates podocytopenia in aged Shroom3-KD mice with podocyte FPE. Shroom3-KD and control mice were age 1 year or older and DOX fed for 6 weeks (*n* = 5 per group). (**A**) Representative WBs of kidney lysates probed for total and phosphorylated Ampk, Turbo-Gfp, and actin of young controls, aged controls, and aged Shroom3-KD mice. Dot plots show (**B**) blood urea nitrogen (mg/dl) in aged control and Shroom3-KD mice. (**C**) Dot plots show podocytes/glomerulus/animal, and box plots (line at median) show distribution of podocytes/glomerulus/group. (**D**) Vglom per group (×1000 μm^3^) between aged control and Shroom3-KD groups. (**E**) Representative PAS images of 2 glomeruli (63×) showing mesangial expansion in aged Shroom3-KD mice (bottom row) versus aged controls (top row). In subsequent experiments, metformin-water (MF) was added at week 2 of DOX to aged control/Shroom3-KD mice. (**F**) Dot plots compare albumin/creatinine ratio (μg/mg) at 6 weeks DOX in aged control versus aged Shroom3-KD mice, both with and without MF treatment. (**G**) Dot plots compare podocytes/glomerulus/animal and box plots (line at median) show distribution of podocytes/glomerulus/group and (**H**) Vglom (×1000 μm^3^) per group between aged control+MF and Shroom3-KD+MF groups. In the box-and-whisker plot, the box represents the middle quartiles, the lines indicate the median, and the whiskers denote the 5th–9th percentile. Line and whiskers indicate mean ± SEM; unpaired *t* test; *P < 0.05, **P < 0.01, ***P < 0.001; WB, Western blot; PAS, periodic acid–Schiff; WT1, Wilms’ tumor 1 protein; 30 glomerular profiles/animal were used for WT1 immunofluorescence; 10 glomeruli/animal were measured for Vglom).

We have previously reported that aged mice (>1 year) with a similar duration of Shroom3 KD developed podocyte loss and early FSGS (10), distinct from young Shroom3-KD mice. To understand whether this loss of podocyte protection during aging was associated with reduced Ampk activation in response to Shroom3 KD (since age-related decline in AMPK activation is also reported elsewhere; refs. 25–27), we studied aged control and Shroom3-KD mice. Using aged versus young controls, we demonstrated reduced pAmpk in kidney lysates and in glomeruli of aged controls (Figure 6A and Supplemental Figure 6, A and B), representing age-related decline of Ampk activation in renal tissues. Further, previously seen enhanced pAmpk in young Shroom3-KD mice was not observed in aged Shroom3-KD mice (Figure 6A vs. Figure 5, E–H). Hence, Shroom3 KD alone was insufficient to enhance AMPK activation in aged kidneys. At 6 weeks of DOX, aged Shroom3-KD mice developed azotemia and podocytopenia (Figure 6, B and C, and Supplemental Figure 6E, respectively**;** *n* = 5 each)**,** in contrast to young Shroom3-KD mice. Mean Vglom was significantly higher in aged KD mice, suggesting an inability to regulate Vglom when Shroom3 KD was not associated with AMPK activation (Figure 6D). PAS staining also showed mesangial expansion in aged KD mice (Figure 6E).

In subsequent experiments, we used an AMPK activator (28), metformin in drinking water (MF; see Methods), to further study the contribution of Ampk to the podocytopenia observed in aged Shroom3-KD mice (*n* = 5 each group). First, MF treatment restored enhanced Ampk phosphorylation with Shroom3 KD in aged Shroom3-KD lysates (significantly greater than in aged controls) (Supplemental Figure 6, C and D). Albuminuria in MF-treated aged Shroom3-KD mice was lower than untreated aged Shroom3-KD mice (Figure 6F). Compared with aged controls, aged Shroom3-KD mice treated with MF did not show podocytopenia at 6 weeks of DOX (Figure 6G). MF-fed aged controls and Shroom3-KD mice showed similar levels of blood urea nitrogen (BUN) and creatinine (Supplemental Figure 6, F and G). MF treatment was also associated with a reduction in mean Vglom in aged Shroom3-KD mice versus aged controls at 6 weeks (Figure 6H), and thus was similar to young Shroom3-KD mice.

These data suggested that in aged Shroom3-KD mice, loss of Vglom regulation and podocytopenia occurred when podocyte FPE occurred in the absence of enhanced AMPK activation. MF use in aged Shroom3-KD mice enhanced AMPK activation and reduced Vglom (versus aged controls), improved proteinuria (versus aged KD mice), and was protective against podocytopenia.

### AMPK inhibition reverses Vglom reduction and promotes podocytopenia in young Shroom3-KD mice.

**Figure 7:**
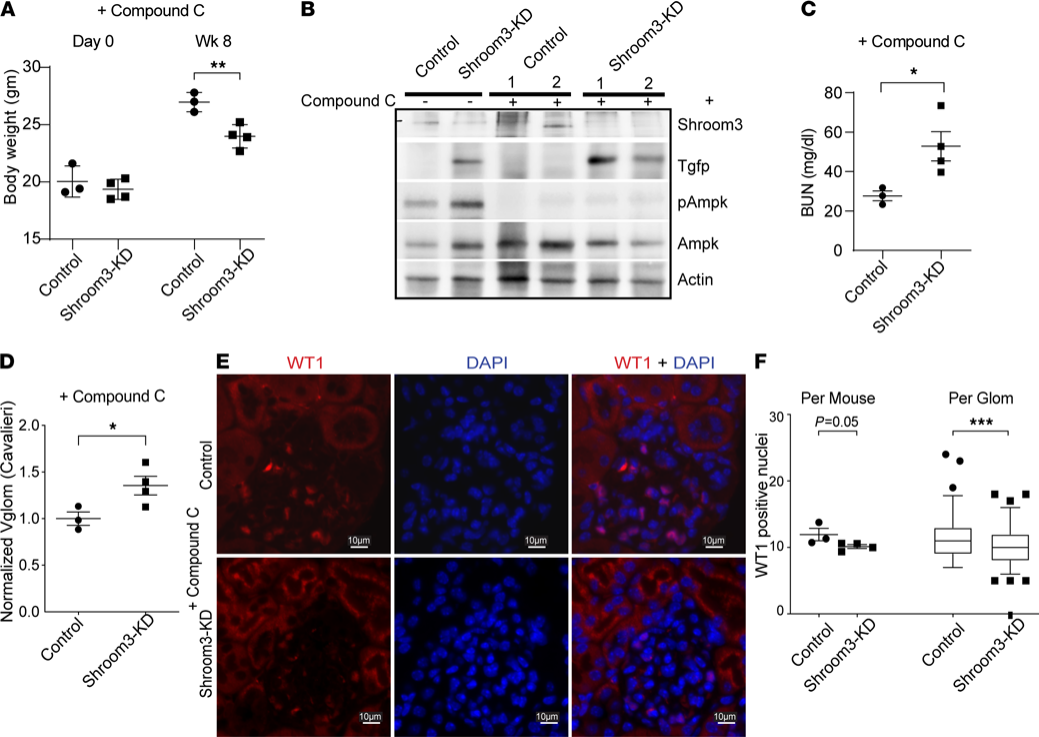
AMPK inhibition reverses Vglom reduction and promotes podocytopenia in Shroom3-KD mice. Shroom3-KD and control mice (~8 weeks old) were DOX fed for 8 weeks. AMPK-inhibitor Compound C was injected (20 mg/kg i.p. × 4 doses at week 5) and mice followed until week 8 when tissues were collected. (**A**) Dot plots show mean body weight (g) (day 0 vs. at week 8) in both groups. (**B**) Representative WBs of whole kidney lysates from control/Shroom3-KD mice confirmed inhibition of AMPK activation by Compound C. Dot plots show (**C**) blood urea nitrogen (BUN in mg/dl) and (**D**) morphometric quantification of Vglom (×1000 μm^3^) in Shroom3-KD and control mice treated with Compound C. (**E**) Representative immunofluorescence images (40×) of WT1 and DAPI stained glomerulus of Shroom3-KD and control mice receiving Compound C. (**F**) Dot plots show podocyte numbers/glomerulus/animal and box plots (line at median) show corresponding distribution of podocytes/glomerulus/group (30 glomerular profiles/mouse). In the box-and-whisker plot, the box represents the middle quartiles, the lines indicate the median, and the whiskers denote the 5th–9th percentile. Line and whiskers indicate mean ± SEM; unpaired *t* test; *P < 0.05, **P < 0.01, ***P < 0.001; WT1, Wilms’ tumor 1 protein.

Next, we studied whether pharmacological AMPK inhibition altered Vglom regulation and reduced podocyte survival in young Shroom3-KD mice with FPE without podocytopenia. We employed Compound C (29), a selective small-molecule competitive AMPK inhibitor acting via its ATP binding site reported to inhibit AMPK activation even in the presence of AMPK activators (30, 31). We administered Compound C at week 5 of DOX feeding (20 mg/kg/dose × 4 doses i.p.) to 8-week-old Shroom3-KD mice and controls (*n* = 4 vs. 3). We aimed to inhibit AMPK activation after inducing Shroom3 KD and podocyte FPE. As shown, Shroom3-KD mice had significantly lower body weight after Compound C administration at 8 weeks versus controls (Figure 7A). Kidney lysate immunoblotting (Figure 7B) and glomerular immunofluorescence (Supplemental Figure 7, A–C) confirmed complete inhibition of Ampk activation by Compound C in both groups of mice. Azotemia was induced by Compound C only in KD mice and not in controls (Figure 7C and Supplemental Figure 7D vs. Supplemental Figure 2, F and L). Most consistently, morphometry revealed loss of Vglom regulation in Shroom3-KD mice after Ampk inhibition (Figure 7D), with increased Vglom and podocyte and capillary/endothelial component measurements versus controls (Supplemental Table 2). Glomeruli of Shroom3-KD mice, which previously showed podocyte FPE but without podocytopenia at 6 and 8 weeks of DOX (Figure 2, B and F; ref. 10), now developed podocyte loss (WT1 staining) (Figure 7, E and F) with Compound C. Hence, inhibition of AMPK by Compound C in young Shroom3-KD mice was followed by loss of protective morphometric changes and induction of podocytopenia.

### AMPK activation reduces Vglom and preserves podocyte numbers in nephron loss–induced glomerular hypertrophy.

Finally, we asked whether pharmacological Ampk activation would promote favorable Vglom regulation and podocyte survival in WT mice. To test this hypothesis, we administered PF0640957 (PF), a highly specific AMPK agonist (32), to BALB/c mice subjected to 5/6th nephrectomy, a model for FSGS resulting from maladaptive hypertrophy of remnant glomeruli and podocytes. BALB/c mice, without or with PF (BALB/c+PF mice) were subjected to 2/3rd nephrectomy, followed by contralateral nephrectomy 7 days later, and euthanized after a further 6 weeks (*n* = 6 vs. 5, respectively; see Methods). PF06409577 gavage was initiated a day before the first surgery.

**Figure 8:**
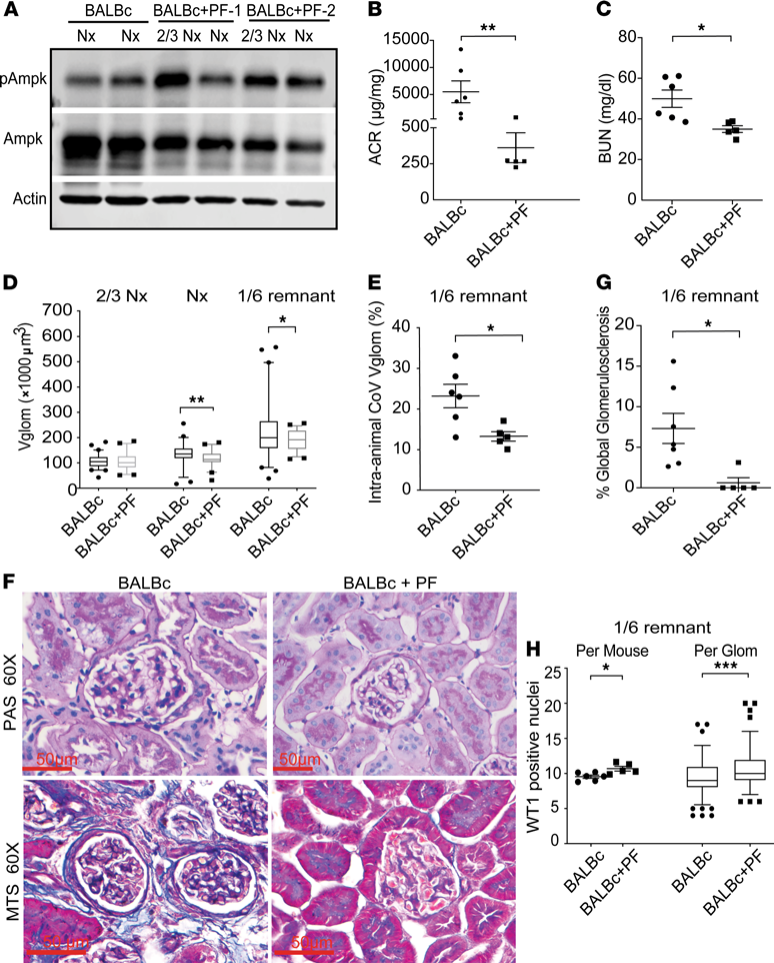
AMPK activation reduces glomerular volume and preserves podocyte numbers in nephron loss–induced glomerular hypertrophy. Adult BALB/c mice (near 8 weeks) underwent 2/3rd nephrectomy (2/3 Nx) followed by contralateral nephrectomy (Nx) 1 week later, and were followed for 6 more weeks when the remaining kidney tissue (1/6th remnant) was harvested. Experimental animals were gavaged with AMPK-activator PF06409577 at 3 doses/week; BALB/c (*n* = 6) versus BALB/c+PF (*n* = 5). (**A**) WBs of lysates from 2/3rd Nx and Nx kidneys showed AMPK activation in BALB/c+ PF versus BALB/c. Dot plots compare (**B**) albumin/creatinine ratio (μg/mg) and (**C**) blood urea nitrogen (mg/dl) at euthanization. (**D**) Box plots show the distribution of Vglom (×1000 μm^3^) in the 2/3rd Nx, Nx, and 1/6th remnants of BALB/c and BALB/c+PF animals (line at median; *n* = 10 glomeruli/sample). (**E**) Dot plots show intra-animal coefficients of variation of Vglom in 1/6th remnants expressed as percentage. (**F**) Representative images (60×) of 1/6th remnants of PAS- (top row) and MTS-stained sections (bottom row) show increased glomerulosclerosis in BALB/c group. (**G**) Percentage of glomeruli in 1/6th remnants with global sclerosis (on MTS stain) are shown. (**H**) Dot plot shows podocytes/glomerular profile/animal by WT1/DAPI stain, and box plots (line at median) show distribution of podocytes/glomerulus in each group (≥20 glomerular profiles per 1/6th remnant). In the box-and-whisker plot, the box represents the middle quartiles, the lines indicate the median, and the whiskers denote the 5th–9th percentile. Line and whiskers indicate mean ± SEM; unpaired *t* test; *P < 0.05, **P < 0.01, ***P < 0.001; WB, Western blot; PAS, periodic acid–Schiff; MT, Masson’s trichrome; glom, glomerulus; WT1, Wilms’ tumor 1 protein.

Baseline BUN was similar in both groups (not shown), and mice showed similar weight loss trends with surgery (Supplemental Figure 8A). Kidney lysates from the BALB/c+PF group obtained from sequential nephrectomy samples confirmed Ampk activation (Figure 8A). BALB/c+PF mice showed significantly attenuated albuminuria (Figure 8B) and improved azotemia (Figure 8C and Supplemental Figure 8B) by euthanization.

We performed morphometry on the serially obtained 2/3rd kidney contralateral nephrectomies (2/3rd Nx) and 1/6th remnants. Baseline Vglom (Figure 8D) and podocyte numbers (Supplemental Figure 8C) from 2/3rd Nx kidneys were similar in both groups. Nephrectomized kidneys and 1/6th remnants in the BALB/c+PF group showed significantly reduced Vglom versus corresponding BALB/c samples (Figure 8D)_._ Further, 1/6th remnants of BALB/c showed widely distributed values of Vglom (Figure 8D), suggesting occurrence of both sclerosed and hypertrophic glomeruli, and BALB/c+PF remnants demonstrated improved Vglom regulation with significantly reduced intra-animal coefficients of variation (Figure 8E). Histologically, BALB/c remnants had significantly more sclerotic glomeruli on Masson’s trichrome stain (Figure 8, F and G), whereas BALB/c+PF 1/6th remnants showed significantly higher podocyte numbers (Figure 8H) and podocyte numerical density (Supplemental Figure 8D). Hence, Ampk agonism regulated Vglom, restricted Vglom hypertrophy, promoted podocyte survival, and mitigated FSGS in a volume stress model of glomerular injury in WT BALB/c mice.

### Transcriptomes of MCD versus FSGS reveal signatures of Fyn kinase inactivation and Ampk activation in MCD glomeruli.

Based on our murine data suggesting a key role for Ampk in transition between an MCD-like pathology to one with podocytopenia and FSGS, we examined human MCD and FSGS cases in a published cohort within NEPTUNE. To test the hypothesis that Ampk may be specifically activated in human MCD cases, we examined expression microarray data from glomerular transcriptomes of MCD (*n* = 9) versus FSGS (*n* = 17) in this cohort (Affymetrix 2.1 ST located at NCBI’s Gene Expression Omnibus, GSE68127; ref. 33). First, we identified significantly upregulated and downregulated differentially expressed genes (DEGs) in MCD to FSGS comparisons from this data set (by LIMMA test *P* < 0.05, i.e., significant DEGs; 916 upregulated and 1044 downregulated DEGs; Supplemental Table 3). These DEGs were input into ENRICHR (34) and analyzed using multiple enrichment platforms. As shown in Supplemental Figure 9, A–D, upregulated DEGs revealed significant enrichment of signals of Fyn kinase inactivation and Ampk activation. Notably, among the top enriched kinases, CSNK1E (casein kinase epsilon isoform) is a canonic Ampk target that links metabolism with circadian rhythm (35, 36), while NUAK2 is an Ampk-like kinase regulated by LKB1 (37) with an identical kinase domain as Ampk and overlapping kinome (38). Consistently downregulated DEGs analyzed using kinase enrichment assay and protein-protein interaction platforms identified LCK as the top enriched kinase (Supplemental Figure 9, E and F). LCK is an Src kinase whose binding activates Fyn and overlaps with the kinome of Fyn (39). However, LCK is not expressed in podocytes (40), whereas Fyn is abundant. Key metabolic signals downstream of AMPK activation (41), PPAR-alpha signaling, and β-oxidation of fatty acids were also significantly enriched in MCD glomerular transcriptomes versus FSGS (Supplemental Figure 9, C and D).

Premade concept nodes directly curated in Nephroseq also demonstrated significantly enriched β-oxidation of fatty acids (top 5% DEGs) and TCA cycle (top 1% DEGs) in MCD versus FSGS glomeruli from this data set using ENRICHR (Supplemental Figure 9, G and H). Hence, these data suggest downregulation of Fyn signaling and upregulation of AMPK signaling within human MCD glomerular transcriptomes compared with FSGS.

**Figure 9:**
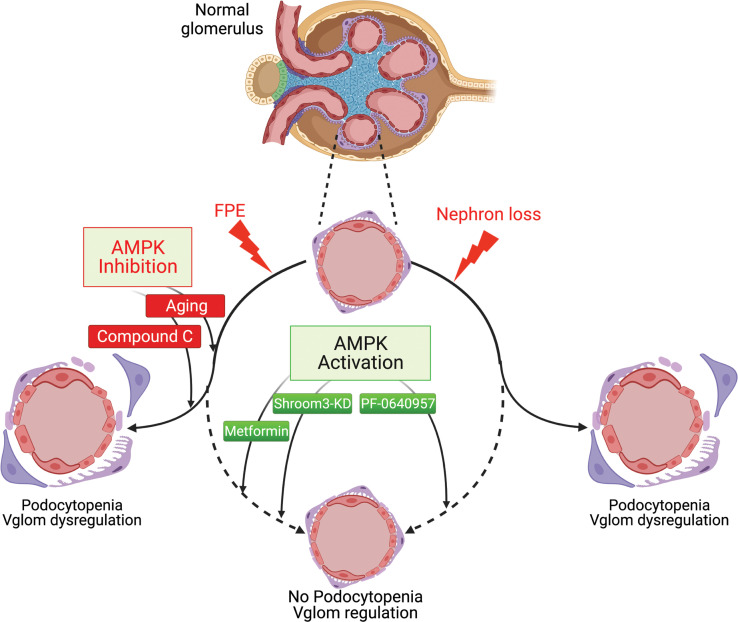
Role of AMPK signaling in glomerular volume regulation and podocyte survival in the context of podocyte injury. Illustration depicts response of normal glomeruli to injury stimuli, i.e., nephron loss in 5/6th nephrectomy or podocyte FPE with Shroom3 knockdown (solid arcuate lines). Sustained hypertrophic stress or Ampk inhibition (Compound C or aging shown as red lines), along with podocyte FPE, led to glomerular volume dysregulation and podocytopenia. In these contexts, Ampk activation (green lines) with PF, MF, or by Shroom3 knockdown (in young mice) preserved glomerular volume regulation and protected against podocytopenia (arcuate dashed lines), thus promoting an MCD-like pathology in young Shroom3-KD mice.

In summary, we revealed AMPK signaling as a key regulator of Vglom and podocyte survival in injured podocytes with FPE or when facing glomerular hypertrophic stress (Figure 9) and translationally demonstrated enrichment of this pathway in human MCD versus FSGS.

## Discussion

Podocyte loss correlates with renal survival in experimental models (8, 42). The association of Vglom and podocyte morphometric parameters with outcomes is reported from human cohorts (3, 5, 43, 44). Hence, identifying novel signals for Vglom regulation in injured glomeruli and podocyte survival mechanisms in the presence of FPE are both crucial. Using a multicenter NS cohort, we showed that MCD diagnosis, where diffuse podocyte FPE does not lead to podocytopenia, was associated with lower Vglom versus FSGS and membranous nephropathy. Furthermore, reduced Vglom by itself was associated with improved renal outcomes. Based on these observations and analogous findings in our young Shroom3-KD mice with diffuse FPE without podocytopenia, we investigated downstream signaling involved in Vglom regulation and podocyte survival. We identified enhanced AMPK phosphorylation with Shroom3 or Fyn KD via LKB1 release (21). Ampk activation was associated with increased autophagy and reduced cellular protein content downstream.

In this regard, we made the important observation here that podocyte-specific *Shroom3* KD regulates not only PodoVglom but also Cap+EndoVglom and total Vglom. These data suggest that primary alterations in podocytes without evidence of podocyte loss can regulate adjacent capillary volume. Recent elegant data using mice with hyperactive Mtor in podocytes similarly showed increases in podocyte size, PodoVglom, and total Vglom before the onset of podocyte loss (4). Although we and others (4) have now demonstrated the regulation of underlying glomerular capillary size by primary changes within podocytes, the mechanisms underlying this finding need examination using coculture experiments and simultaneous interrogation of podocyte-endothelial transcriptomes to unravel crosstalk pathways that are initiated in the podocyte and involved in Vglom regulation.

Ampk activation regulates several downstream signaling pathways, including anabolism, autophagy, energy conservation, and mitochondrial homeostasis (reviewed by Carling; ref. 45). Among these, identifying the central pro-survival mechanism(s) after podocyte AMPK is activated needs further examination. AMPK regulates cell growth by modulating mTORC1 signaling but also by directly inhibiting ribosomal biogenesis and protein translation (by phosphorylating EF2) and activating autophagy (22, 23). We showed here that AMPK activation in podocytes regulated podocyte volume, albeit without significant changes in MTOR phosphorylation (4). This is consistent with recent work showing the in vivo role of Ampk in the regulation of podocyte autophagy (46, 47). This is also relevant given that the AMPK/autophagy axis is reported to be protective in FSGS models (46, 48). Reduced 18S RNA seen here with increased pAMPK is likely from inhibition of RNA-polymerase-I by PRKAG2 (γ-2 subunit of AMPK) also expressed by podocytes (23).

We and others (49–52) have demonstrated that Ampk activation can mitigate FSGS and improve podocyte survival in animal models. Our experiments further revealed that in Shroom3-KD mice with podocyte FPE (10), coincidental AMPK activation regulated Vglom and prevented podocytopenia, promoting an MCD-like pathology. The critical role of Ampk activation in preventing glomerular enlargement, podocytopenia, and azotemia was demonstrated in aged mice (in which Ampk activation is reduced) and after pharmacological Ampk inhibition by Compound C. Most importantly, activating Ampk in podocytes with Shroom3 KD induced FPE or after nephron loss in WT mice, promoted adaptive Vglom regulation and prevented podocytopenia. While the beneficial role of AMPK activators in proteinuric disease in diabetes or obesity is established (50, 53–56), our data describe a role for AMPK signaling in preventing podocytopenia and restricting glomerulomegaly in the context of podocyte FPE, in turn controlling the MCD-FSGS transition. Further experimental data are essential to study the modulation of the podocyte Ampk signaling axis by established circulating mediators of CKD and podocyte injury, such as SUPAR (57, 58), TGF-β (9, 59), and TNF-α (60, 61) that could then link podocytopenia, glomerulomegaly, and progressive disease with Ampk signaling in each of these instances. More translational work is also needed to examine enriched Ampk signaling genes as biomarkers of an MCD-like clinical course in NS cases and in understanding the role of Ampk activation in mitigating specific role in mitigating the transition to FSGS by sustaining podocyte survival during injury with FPE. These data are therefore an important platform to examine AMPK-based therapeutics in human NS using approved AMPK activators (62) or LKB1-based agents (63).

We have not addressed Fyn knockout in vivo (64). We acknowledge this as a limitation of our work. However, global Fyn-KO mice had FPE without podocyte loss until at least 1 year of age (similar to Shroom3-KD mice) and reduction in cell size, suggesting in vivo Ampk activation (13, 21). We cannot completely rule out ancillary mechanisms of Ampk activation by cytoskeletal or metabolic stressors in Shroom3-KD cells. Since we used global Shroom3-KD mice, our current data also cannot exclude beneficial effects of Ampk activation (by MF or PF) and deleterious effects of Ampk inhibition in nonpodocyte cells as contributing to observed renal phenotypes.

In summary, utilizing a Shroom3-KD murine model, we revealed the role of Ampk signaling in the regulation of podocyte and Vglom. We applied an aging model and pharmacological agents to demonstrate the key role of AMPK in regulating podocyte survival in injured glomeruli with podocyte FPE. These findings are of importance to podocyte biology and pathology in NS and have considerable application to therapeutics in glomerular diseases given the availability of AMPK activators.

## Methods

The following is a summary of the methods used. See Supplemental Methods for more detailed methods.

### Cell culture.

A human podocyte cell line (gift from Moin Saleem, University of Bristol, Bristol, United Kingdom) was differentiated using RPMI-1640. Protein/DNA ratio assay was done using 1000-podocytes/well in 96-well plates. in vitro autophagy studies were performed using Bafilomycin A1 at day 7 of differentiation (100 nM for 24 hours) (24).

### Reverse transcription qPCR.

Transcript expression was assayed by real-time PCR using specific primers (see Supplemental Table 4). Amplification curves were analyzed via the 2^–ΔΔCt^ method.

### Western blotting.

Cells were lysed for immunoblot analysis as described previously (18) and probed using polyclonal SHROOM3 rabbit antibody (SAB3500818, MilliporeSigma), Fyn rabbit polyclonal antibody (4023S, Cell Signaling), Actin mouse monoclonal antibody (AC-15) (A5441, MilliporeSigma), phospho-mTOR (Ser2448) rabbit monoclonal antibody [EPR426(2)] (ab109268, Abcam), mTOR (Ser2448) rabbit polyclonal antibody (ab2732, Abcam), phospho-AMPK (Thr172) rabbit polyclonal antibody (2535, Cell Signaling), AMPK rabbit polyclonal antibody (2532, Cell Signaling), phospho-eEF2 (Thr56) rabbit polyclonal antibody (2331, Cell Signaling), eEF2 rabbit polyclonal antibody (2332, Cell Signaling), turboGFP mouse monoclonal antibody (OTI2H8) (TA150041, CiteAb), Phospho-ULK1 (Ser555) rabbit polyclonal antibody (5869, Cell Signaling), ULK1 (D8H5) rabbit monoclonal antibody (8054, Cell Signaling), and LC3A/B rabbit polyclonal antibody (4108, Cell Signaling).

### shRNA suppression studies.

Human *SHROOM3* (9, 10) and *FYN* short hairpin clones (Dharmacon, Inc.) were tested for optimal suppression in 293T cells, HK-2 cells, and human podocytes to generate stable cell lines.

### Immunofluorescence.

For immunofluorescence, 5 μm paraffin sections of formalin-fixed kidney-tissues were deparaffinized and processed for unmasking and antigen retrieval followed by incubation with primary antibodies.

### Quantitative image analysis.

For pAMPK, glomeruli were outlined using Zen Pro 2.6 (blue edition) software (40× images), and the areas of pAMPK staining were measured for signal intensity and expressed as average signal intensity total area/glomerulus.

### Murine Shroom3-KD model.

Double transgenic mice, chicken β-actin promoter, reverse tetracycline transactivator (rtTA)/Shroom3-shRNA mice CAGS-rtTA or Nphs1-rtTA (gifts from Jeffrey H. Miner, Washington University School of Medicine, St. Louis, Missouri, USA; ref. 19) were generated for global- or podocyte-specific KD (i.e., Shroom3-KD or podocyte-Shroom3-KD, respectively). Male mice (~8–12 weeks old) were DOX-fed for 6 weeks. For aging studies, Shroom3-KD and BALB/c control mice were aged 1 year or older before DOX. Kidney tissues and glomeruli were processed (See Supplemental Methods). For uninephrectomy, DOX-fed male Shroom3-KD/podocyte-Shroom3-KD versus control mice were nephrectomized and kidneys saved. Residual kidney was collected at 1 week. For 5/6th nephrectomy, surgery was performed to remove 2/3rd of the left kidney. After 7 days, contralateral nephrectomy was done. At each surgery, tissues were saved for studies (65). For AMPK inhibition studies using Compound C, after 4 weeks DOX, mice were injected with Compound C (20 mg/kg × 4 doses) and euthanized at 8 weeks. For AMPK activation studies, 5/6th nephrectomy mice were administered PF-06409577 (gavage 50–100 mg/kg; see Supplemental Methods) (65). The MF protocol was a dose of 500 mg/500 mL in drinking water (66) added at week 2 of DOX. For in vivo sample assays, BUN measurement was performed on serum samples using Quantichrom assay (BioAssay Inc.), and serum creatinine was assayed using StatSensor assay (Nova Biomedical Inc.; see Supplemental Methods).

### Vglom — Weibel-Gomez method.

The Weibel-Gomez method was used to measure Vglom in human NS biopsies. This method uses 1 PAS-stained and 1 trichrome-stained paraffin section from Aperio-scanned images of NS biopsies from the NEPTUNE study (5). The areas of all complete glomerular profiles present in the section were measured by planimetry (Supplemental Figure 1A). Vglom = A^3/2^ × 1.38 μm^3^ where A is the average glomerular tuft area and 1.38 is the shape correction factor assuming glomeruli are spheres (67). The mean Vgloms obtained from 2 sections within each patient were highly correlated (Supplemental Figure 1B). The PAS-stained sections were used for stereological analyses.

### Vglom — Cavalieri method.

One-millimeter cubes were cut from the cortex, fixed in glutaraldehyde, and embedded in Epon. Vglom was measured using 1 μm thick sections and the Cavalieri principle (Supplemental Figure 2A). As described previously (10, 18), high-magnification images (~1700×) were used for estimation of Vglom components (Supplemental Methods; Supplemental Figure 2, B–D), i.e., PodoVglom, Cap+Endo Vglom, and mesangium were calculated by measuring the volume fraction of each component and then multiplying each fraction by the Vglom. Podocytes were counted using the fractionator/disector method (Supplemental Methods; ref. 68). The morphometrist was blinded to disease group for the human biopsies and to experimental group in murine data.

### FPW quantification.

For quantitative ultrastructural analysis of the glomerulus by transmission electron microscopy (the number of podocyte foot processes present in each micrograph was divided by the total length of GBM; to calculate the mean density of podocyte foot processes. Capillary loops in at least 3 separate glomeruli of each animal, adding up to a mean of 1088 ± 339.6 foot processes, were counted over an average of 467.9 ± 113.7 mm of the basement membrane. The thickness in each image was measured using ImageJ (NIH).

### Data collection.

Publicly available human microarray data sets for all kidney diseases, including FSGS, membranous nephropathy, MCD, and others, were downloaded from NCBI’s Gene Expression Omnibus (GEO GSE68127). We collected high-throughput transcriptome data for 99 disease and control samples. Within this data set we manually selected the samples with clinical information at GEO. For each study, we grouped the samples with the clinical and phenotypic information reported by the original study. Then, for the raw microarray data, we performed quality assessment, and all the microarray platform data were reannotated to the most recent NCBI Entrez Gene Identifiers by AILUN (http://ailun.ucsf.edu). All the expression values were base-2 log-transformed and normalized by quantile-quantile normalization.

### DEG identification.

Principal component analysis was first performed to assess the sample correlations using the expression data of all the genes. The LIMMA test was applied for analysis of data. A specific gene was considered differentially expressed if the *P* value given by these methods was less than or equal to 0.05.

### Pathway network, generation, and analyses.

The significant DEGS from tubular and glomerular microarray data were identified by comparing between MCD and FSGS cases in this data set. We used two methods to perform to perform gene enrichment analysis. INGENUITY IPA (www.ingenuity.com/products/ipa) and Enrichr (https://amp.pharm.mssm.edu/Enrichr/) were used for GO and pathways for DEGs with a fold-change cutoff of 1.5 or higher.

### Statistics.

Deidentified clinical information was obtained for the NS morphometry cohort and linked to morphometry using unique IDs. For human data, univariate comparisons of clinical/demographic factors between NS categories were done using 1-way ANOVA (Kruskal-Wallis for corresponding nonparametric analysis with Dunn’s post test) for continuous variables and χ^2^ for proportions. Spearman’s correlation coefficient was used to compare Vgloms within patients. Cox proportional hazard models were used for multivariable survival associations, including clinical/demographic factors identified as significantly different in univariable analyses. NEPTUNE-determined outcomes of end-stage renal failure, eGFR decline 40% or greater from baseline, or a composite of these events were evaluated as outcomes. Time from biopsy to event was utilized. For in vitro and in vivo MF experiments, an unpaired 2-tailed *t* test (Mann-Whitney test for corresponding nonparametric analysis) was used for data between 2 groups. The cutoff for statistical significance was a 2-tailed *P* value less than 0.05.

### Study approval.

IRB-approved protocols for clinical data (HS no. 19-00600 from Icahn School of Medicine at Mount Sinai and IRB no. -2000030284 from Yale University school of Medicine) and IACUC-approved protocols for mouse experiments (no. 2021-20363 from Yale University school of Medicine and no. 18-2082 from Icahn School of Medicine at Mount Sinai) were obtained.

## Author contributions

KB, QL, JCH, and MCM contributed to the research/study design. KB, QL, CW, MP, AC, FG, and AG performed experimentation. FG, MP, QL, KB, and MCM did mouse husbandry. JMB and KVL performed morphometry. FS and LL conducted histology. KB, QL, WZ, NC, and MCM analyzed data. AS, LK, BD, BM, and JCH provided reagents. KB, JCH, and MCM wrote the manuscript. All authors contributed to the editing of the manuscript.

## Supplementary Material

Supplemental data

## Figures and Tables

**Table 1:**
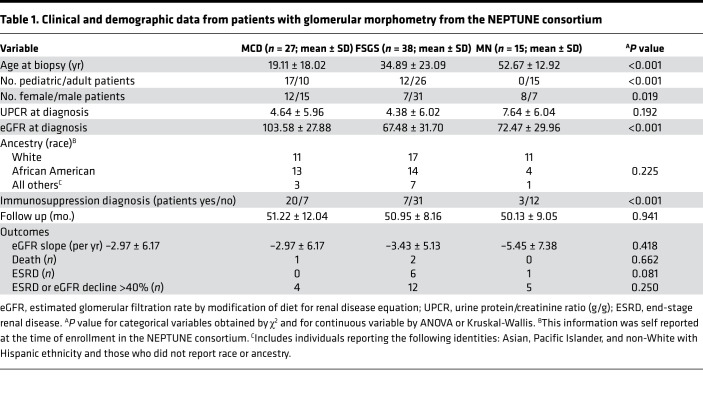
Clinical and demographic data from patients with glomerular morphometry from the NEPTUNE consortium

**Table 2:**
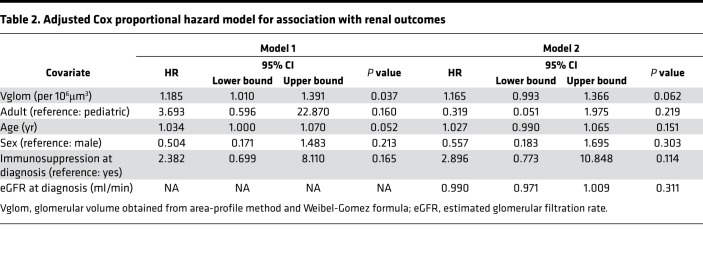
Adjusted Cox proportional hazard model for association with renal outcomes

## Version 1 09/02/2021

In-Press Preview

## Version 2 10/08/2021

Electronic publication
