# Supplementary material for: AMPK mediates regulation of glomerular volume and podocyte survival
Source: JCI Insight. 2021 Oct 8;6(19):e150004. doi: 10.1172/jci.insight.150004 (PMC8525649; doi:10.1172/jci.insight.150004)
Supplement: Supplemental data [file jciinsight-6-150004-s276.pdf]

Figure S1

A B

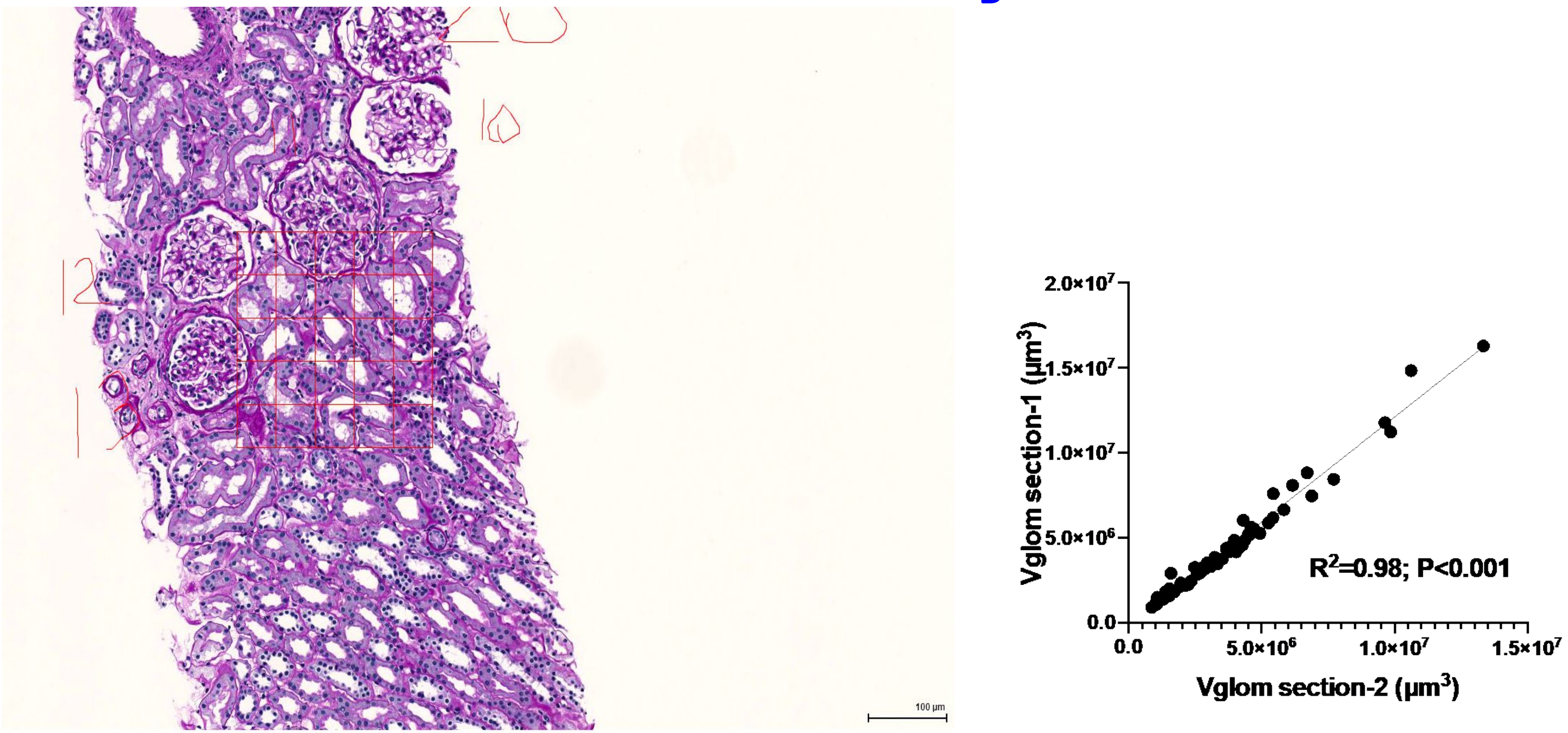

Figure S1. Glomerular morphometry shows significantly lower Vglom in MCD vs FSGS cases. (1A) Aperio-scanned image of representative NS biopsy from NEPTUNE study. Glomerular volumes were calculated from the Weibel-Gomez method from area cross sections of glomeruli measured by planimetry on PAS images. Weibel-Gomez method: Vglom = 1.38 x Area$^{3/2}$ where Vglom is the glomerular volume, 1.38 is the assumed shape factor for the glomeruli, and Area is the average cross-sectional tuft area of the glomerular profiles measured. Image adapted from Lemley et al, PLOSone, 2016 (5). **(1B)** Correlation plot of Vglom (in μm^3) from two random sections obtained with the same biopsy (R^2=0.98; P<0.001; Spearman R). [PAS= periodic acid Schiff]

Figure S2A-B

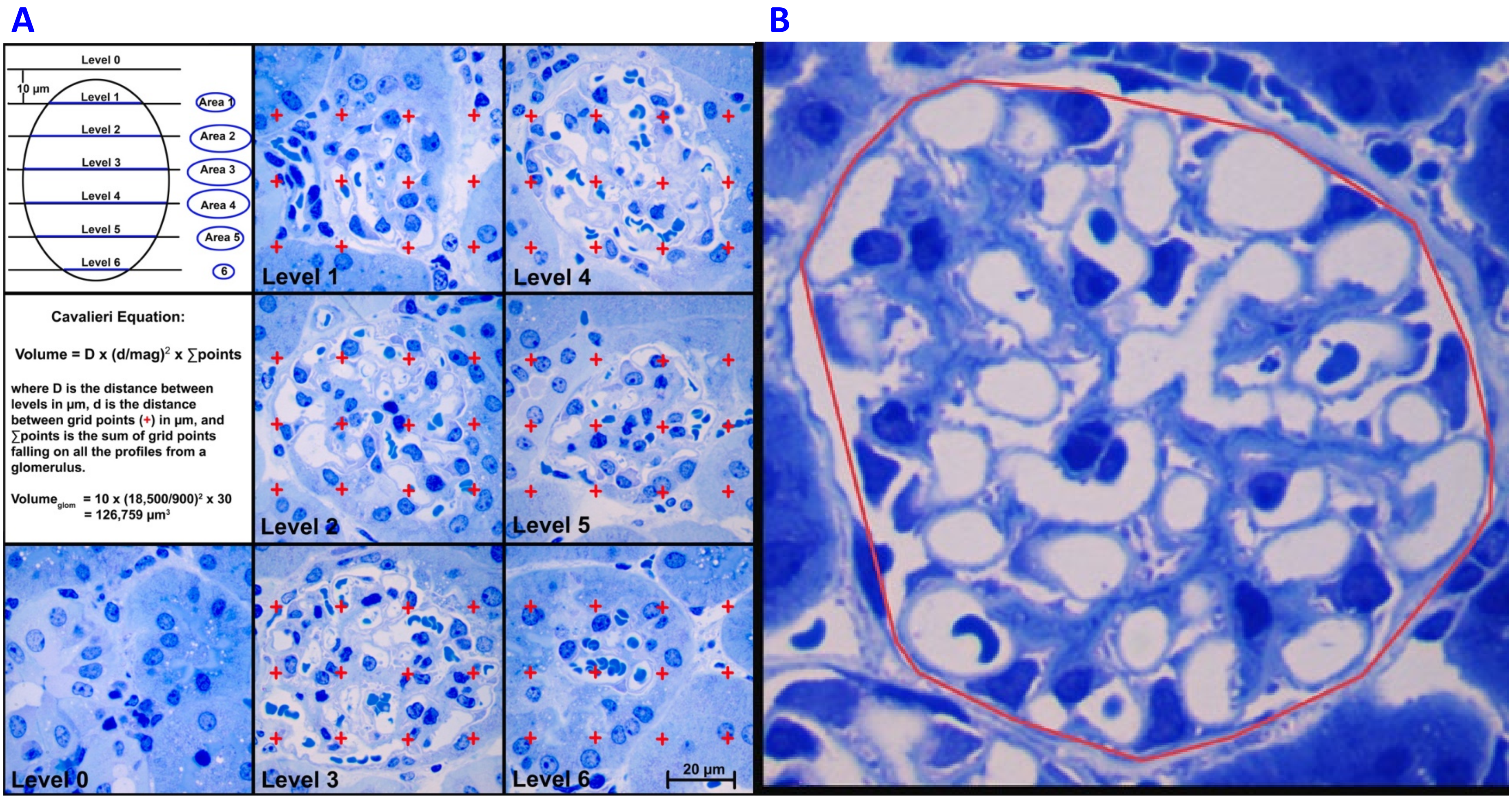

Figure S2A-B. Global or Podocyte specific Shroom3 knockdown reduced glomerular and podocyte volume: (2A) Demonstration of Cavalieri method to measure Glomerular volume. Upper left panel demonstrates the sampling scheme for the Cavalieri method to measure volume of an arbitrary particle. Middle left panel shows the Cavalieri equation. Remaining panels are images through a sample glomerulus with grid points superimposed over the toluidine blue epon sections. **(2B)** Representative image used for Vglom component analyses. Glomerular profile is defined by a minimal polygon around the glomerular profile in image **(see 2C & 2D & methods)**

Figure S2C-D

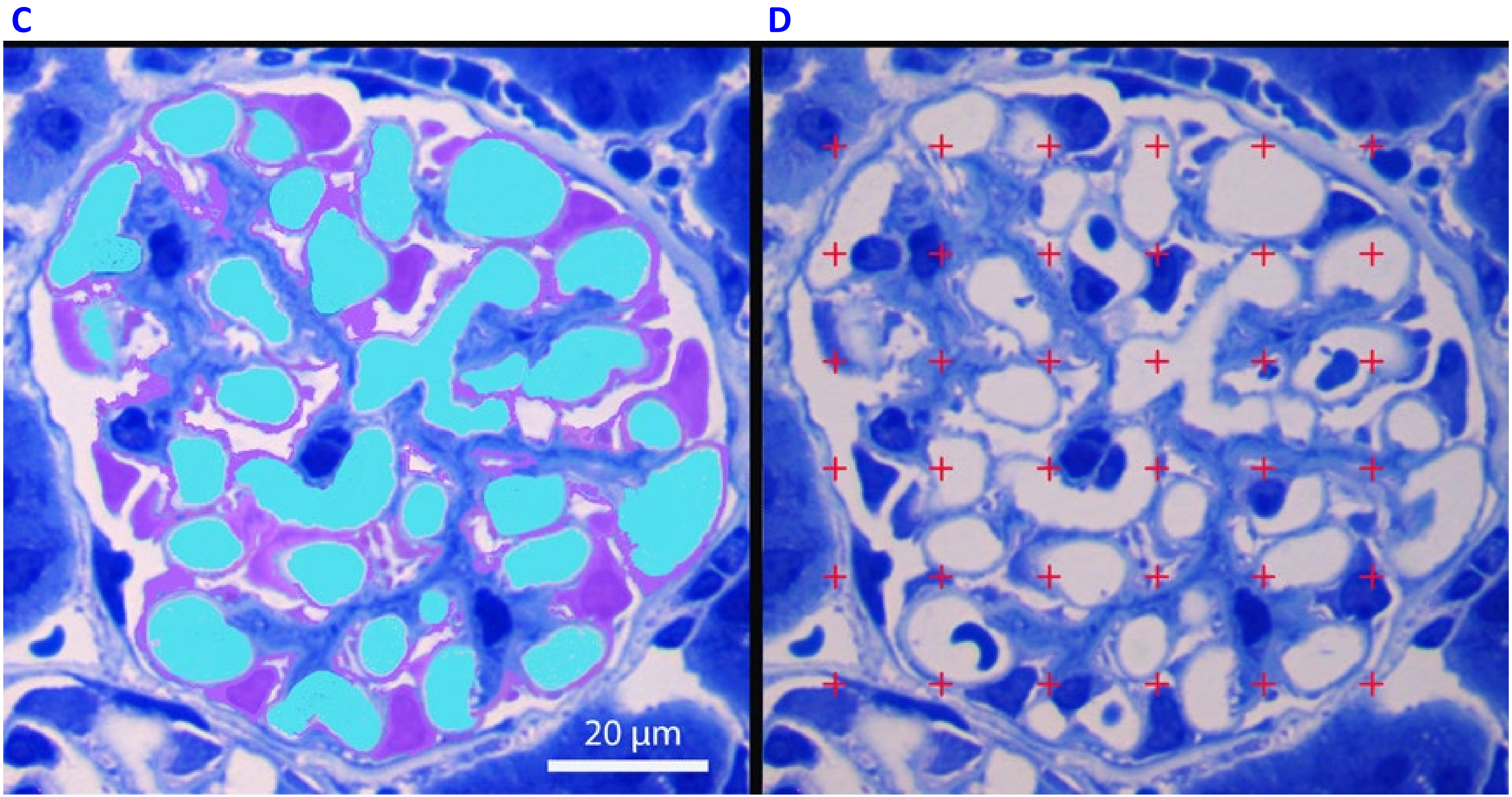

Figure S2C-D. Global or Podocyte specific Shroom3 knockdown reduced glomerular and podocyte volume (continued): Representative image used for Vglom component analyses are shown. Glomerular profile is defined by a minimal polygon around the glomerular profile in image as in **2B**. **(2C)** Each glomerular profile is divided into four Vglom components as demarcated in pseudocolor – podocytes (purple), Capillary space+endothelial cell (cyan), mesangium (original blue), and other (white within polygon). **(2D)** A counting grid is randomly placed over the glomerular profile and number of points falling on each component is counted. 10 gloms &~ 600 points were counted per kidney.
Formula: ***Volume density of Component X=Σ points on X/ Σ points on Glomerulus***

Figure S2E-K

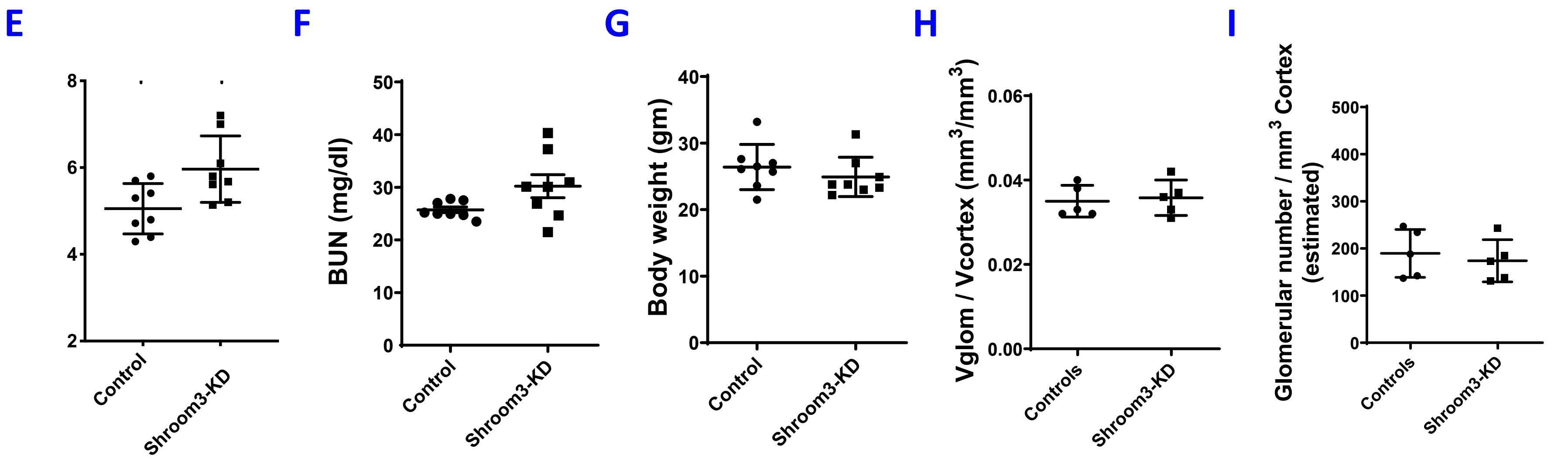

Figure S2E-I. Global or Podocyte specific Shroom3 knockdown reduced glomerular and podocyte volume: Control & Shroom3 KD mice were DOX fed for 6 weeks (n=8 each). Dot plots compare **(2E)** mean podocyte nuclear density (per μm^3 Vglom), **(2F)** mean blood urea nitrogen levels (mg/dl), and **(2G)** body weights (gms). In a subset of 5 mice, dot plots compare **(2H)** glomerular volume density (Vglom / Vcortex (mm^3/mm^3)) and **(2I)** glomerular numerical density (Glomerular number / mm^3 Cortex; Glomerular profiles counted =160±37.3, Mean±SD). [Line/Whiskers=Mean/SEM; *= P<0.05].

Figure S2M-N

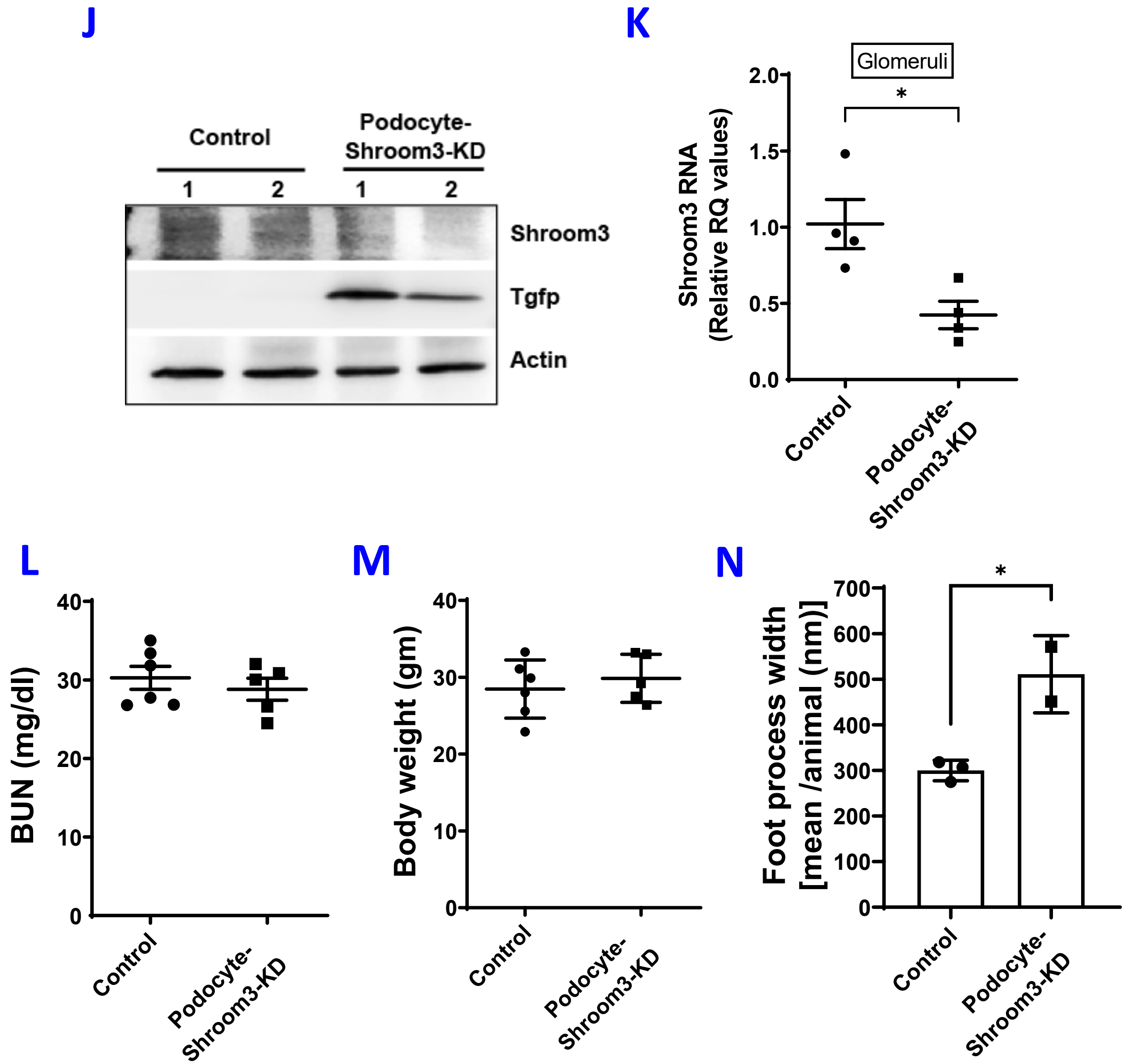

Figure S2J-N. Global or Podocyte specific Shroom3 knockdown reduced glomerular and podocyte volume: Similarly, Control and Podocyte-Shroom3-KD mice were DOX fed for 6-weeks. **(2J)** Representative WBs of Glomerular lysates (n=2 each group) demonstrate immunoblotting for Shroom3, TurboGFP, and Actin. Dot-plots compare **(2K)** copy numbers of Shroom3 transcripts normalized to Nphs1/Actin, in Glomerular RNA extracts from Control vs Podocyte-Shroom3-KD mice (n=4 each group), **(2L)** mean blood urea nitrogen levels (mg/dl), and **(2M)** body weights (gms) of control and Podocyte Shroom3-KD mice (n=6 vs 5) **(2N)** Bar graph compare Foot process width quantification (FPW (nm)) in representative Control vs Podocyte-Shroom3-KD mice (n=3 vs 2, respectively). [Line/Whiskers=Mean/SEM; *= P<0.05, RQ=Relative quantity].

Figure S3

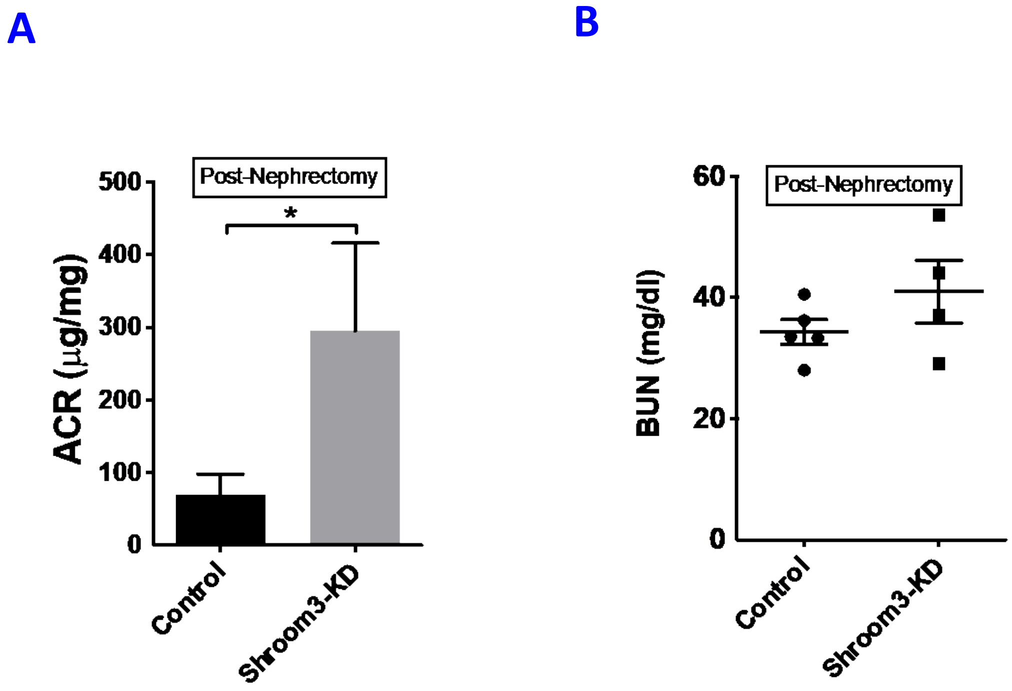

Figure S3. Shroom3 knockdown restricted glomerular hypertrophy post-unilateral nephrectomy: Control & Shroom3-KD mice underwent unilateral nephrectomy (n=5 vs 4). Remnant kidney was evaluated at 1-week post nephrectomy. Bar graph show Albumin: creatinine ratio (µg/mg) **(3A),** and dot plot show BUN (mg/dl) **(3B)** at 1 week post nephrectomy**.** [Line/Whiskers=Mean/SEM; *= P<0.05].

Figure S4

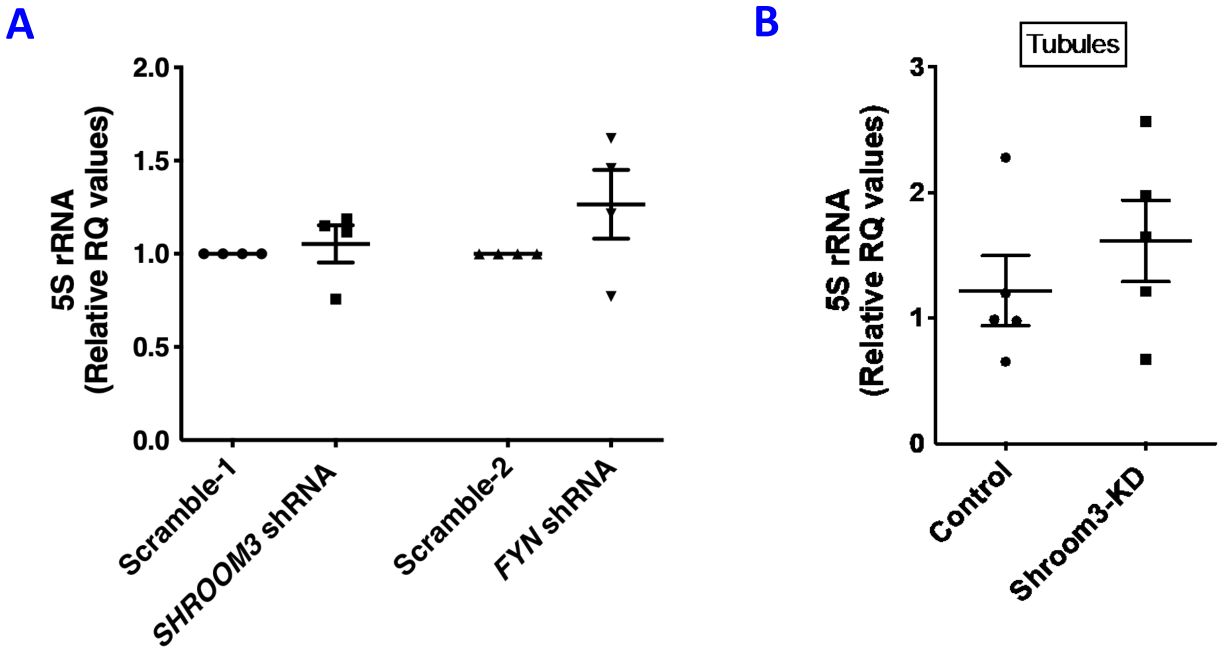

Figure S4. Shroom3 knockdown reduces cellular protein content and RNA biogenesis *in vitro* and *in vivo* mediated via FYN: (4A) Puromycin-selectable, stable Shroom3 and Fyn knockdown podocytes were generated using lentiviral shRNA infection (Scramble-1 and Scramble-2 are respective scramble-sequence infected controls). Stable podocytes were differentiated (>7 days) in collagen coated plates. In SHROOM3- & FYN-shRNA podocyte lines, dot plots show paired comparisons (n=4 sets) of copy numbers of 5S subunit of ribosomal RNA (5S rRNA) normalized to Actin, vs Scramble infected podocyte lines **(4B)** Dot plots compare copy numbers of 5S rRNA normalized to Actin, in tubular RNA extracts (n=5 vs 5 Control vs Shroom3-KD mice). [Line/Whiskers=Mean/SEM; compared by paired t-test (A) and unpaired t-test (B); RQ=Relative quantity].

Figure S5A-D

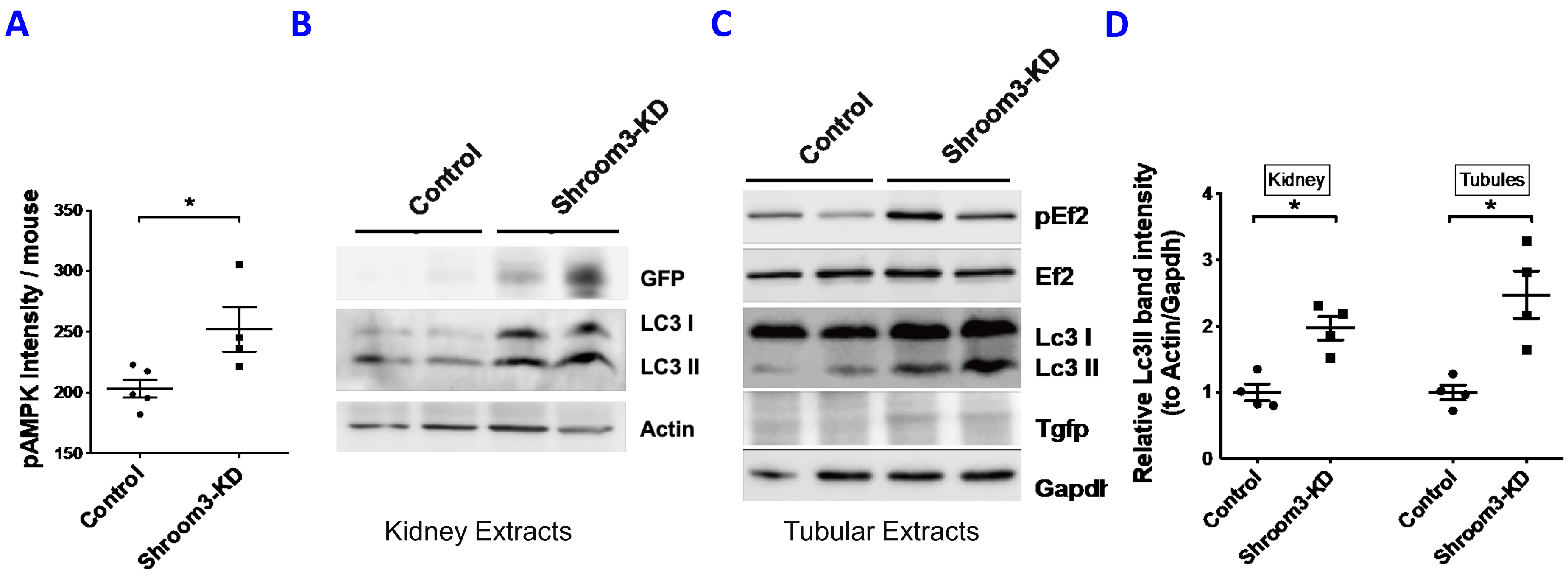

Figure S5A-D. Shroom3- or Fyn knockdown increase cellular AMPK activation: (5A) Dot plots show quantification of intensity of Phosphorylated AMPK/per glomerular outline per group (depicted per animal). Representative images of immunoblots from Control vs Shroom3-KD (n=2 each) showing **(5B)** GFP, Lc3 I & II and Actin bands in kidney extracts and **(5C)** showing immunoblots from kidney tubular extracts from Control vs Shroom3-KD (n=2 each) showing pEf2, Ef2, Lc3 I & II, Tgfp and Gapdh bands. **(5D)** Dot plots quantify respective relative band intensity of Lc3-II in Control vs Shroom3-KD lysates (normalized to Actin/Gapdh; n=4 mice). [Line/Whiskers = Mean/ SEM; *= P<0.05; unpaired t-test].

Figure S5E-F

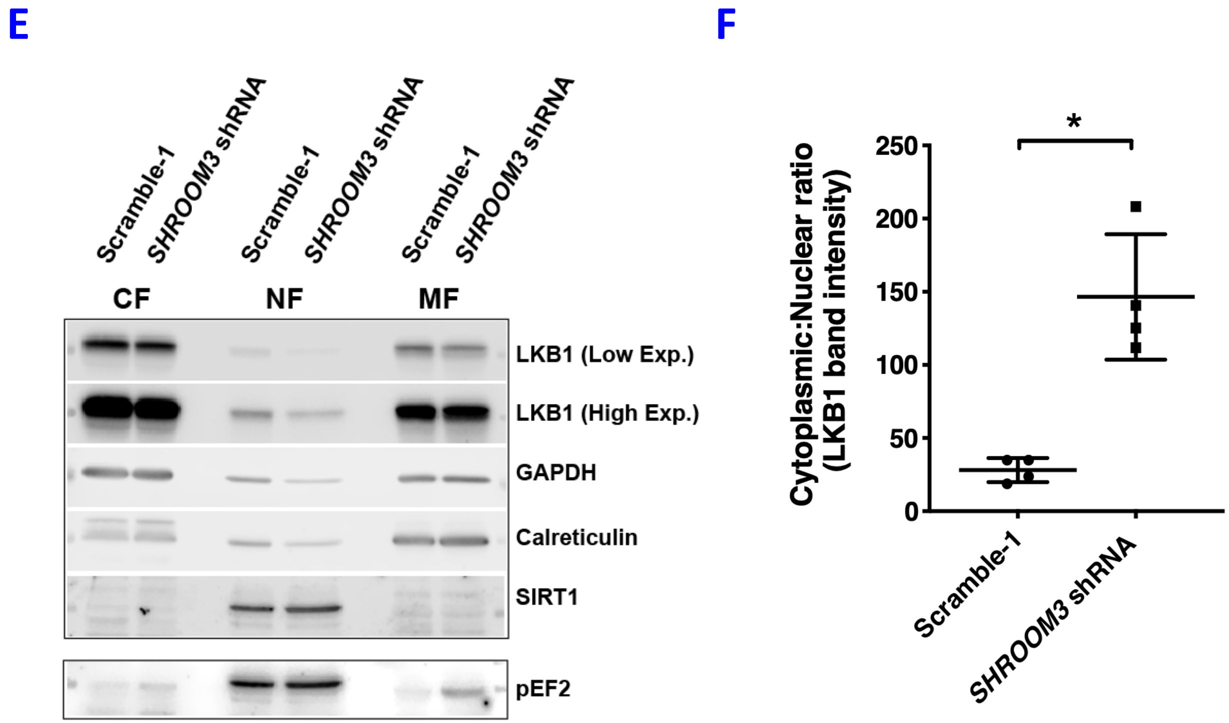

Figure-S5E-F. Shroom3- or Fyn knockdown increase cellular AMPK activation: Reduced nuclear retention of LKB1 with FYN inactivation or knockdown results in LKB1-mediated AMPK activation in podocytes. **(5E)** Representative WBs of lysates obtained after sub-cellular protein fractionation (CF/NF/MF=Cytoplasmic-/Nuclear-/Membrane Fractions, respectively) from Scramble-1, *SHROOM3*-shRNA podocytes probed for LKB1, Phosphorylated EF2; additionally, GAPDH, Calreticulin, SIRT1 were used as fractionation controls for CF, MF and NF, respectively. **(5F)** Dot plots show the respective relative band intensity of LKB1 (CF:NF ratio; n=4 sets). [Line/Whiskers=Mean/SEM; unpaired t-test; *= P<0.05].

Figure S5G

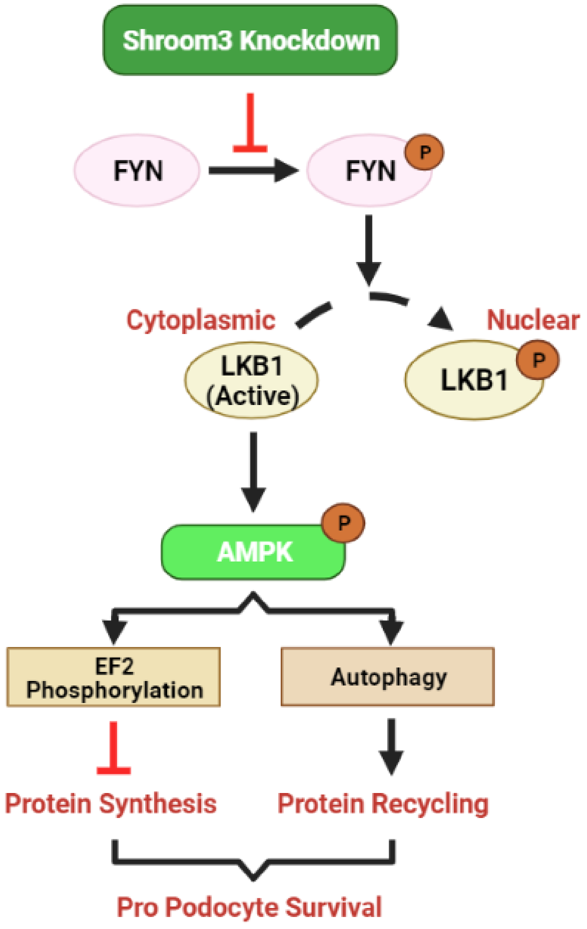

Figure-S5G. Shroom3- or Fyn knockdown increase cellular AMPK activation: Signaling schema summarizes AMPK activation in podocytes with Shroom3 knockdown mediated via LKB1 cytoplasmic redistribution downstream of Shroom3-Fyn axis. AMPK activation regulates cell and glomerular size by engaging downstream mechanisms - reduced anabolism and increased autophagy.

Figure S6

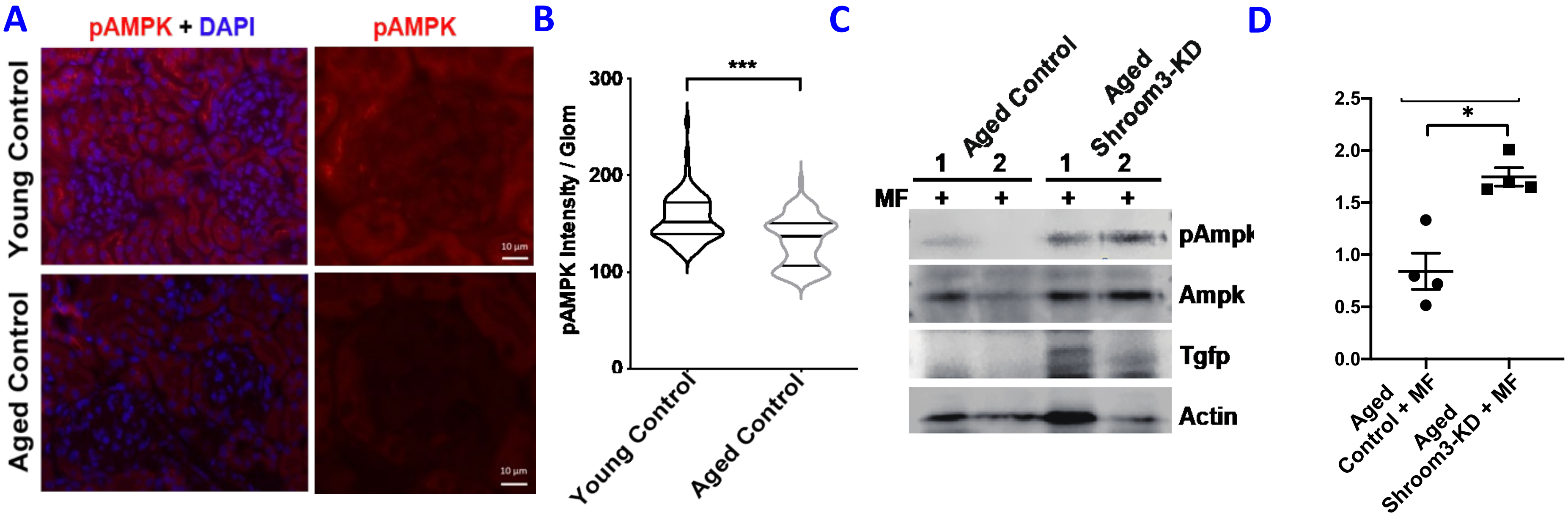

Figure S6A-D. AMPK activation reduces Vglom and mitigates podocytopenia in aged Shroom3 knockdown mice with podocyte FPE: As described previously, Control & Shroom3-KD mice were aged > 1-year and DOX feeding for 6 weeks. In subsequent experiments, at week-2, Metformin-water (MF) was added. **(6A)** Representative immunofluorescence images showing Phosphorylated AMPK/WT1/DAPI in young Controls, & Aged Shroom3-KD mice **(6B)** Violin -plots show quantification of intensity of Phosphorylated AMPK/per glomerular outline per group (depicted per glomerulus; 30 gloms/animal; n=5 each). (**6C)** Representative images of immunoblots from Aged Controls vs Shroom3-KD + MF (n=2 each) showing Phospho-Ampk, Ampk, Tgfp and Actin **(6D)** Dot plots show the respective relative band intensity of pAmpk:Ampk (to Actin; n=4) [Line/Whiskers=Mean/SEM; unpaired t-test; *= P<0.05, ***= P<0.001].

Figure S6

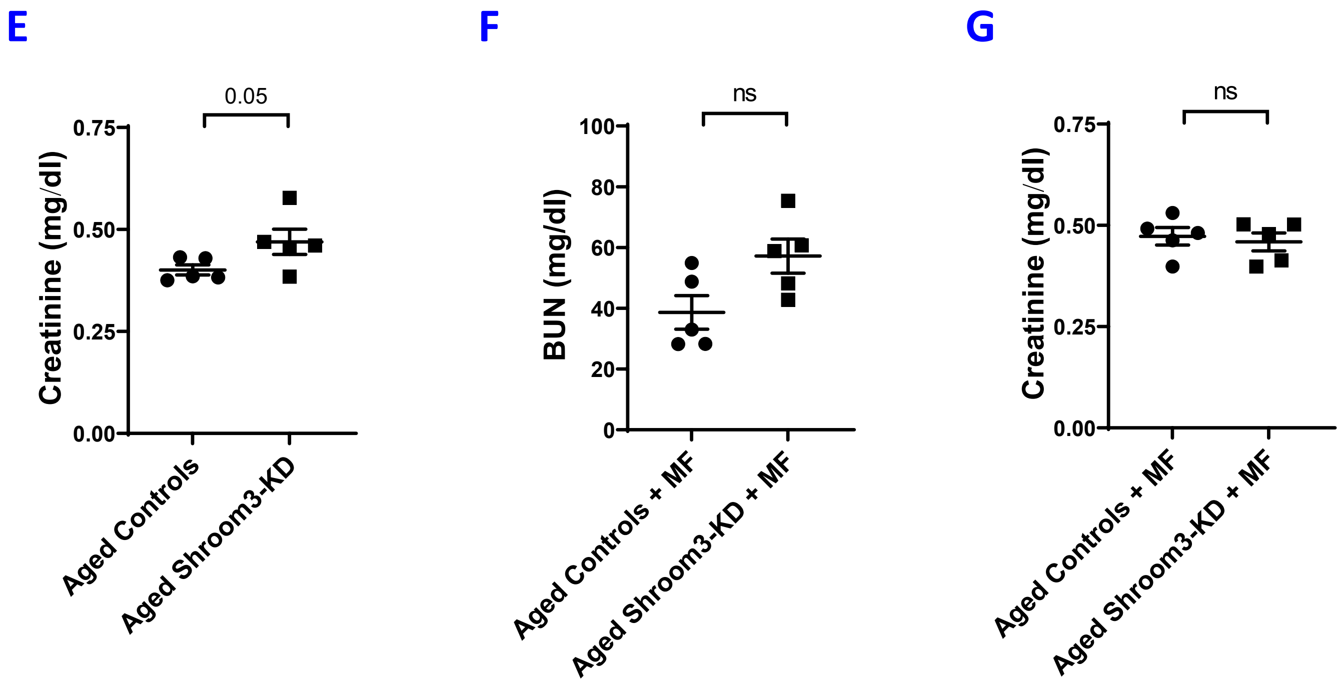

Figure S6E-G. AMPK activation reduces Vglom and mitigates podocytopenia in aged Shroom3 knockdown mice with podocyte FPE: Dot plots compare **(6E)** Creatinine (mg/dl) levels among Aged Controls and Shroom3-KD mice **(6F)** mean blood urea nitrogen (mg/dl) and **(6G)** Creatinine (mg/dl) levels among Aged Controls and Shroom3-KD mice fed with Metformin (n=5) [Line/Whiskers=Mean/SEM; *= P<0.05].

Figure S7

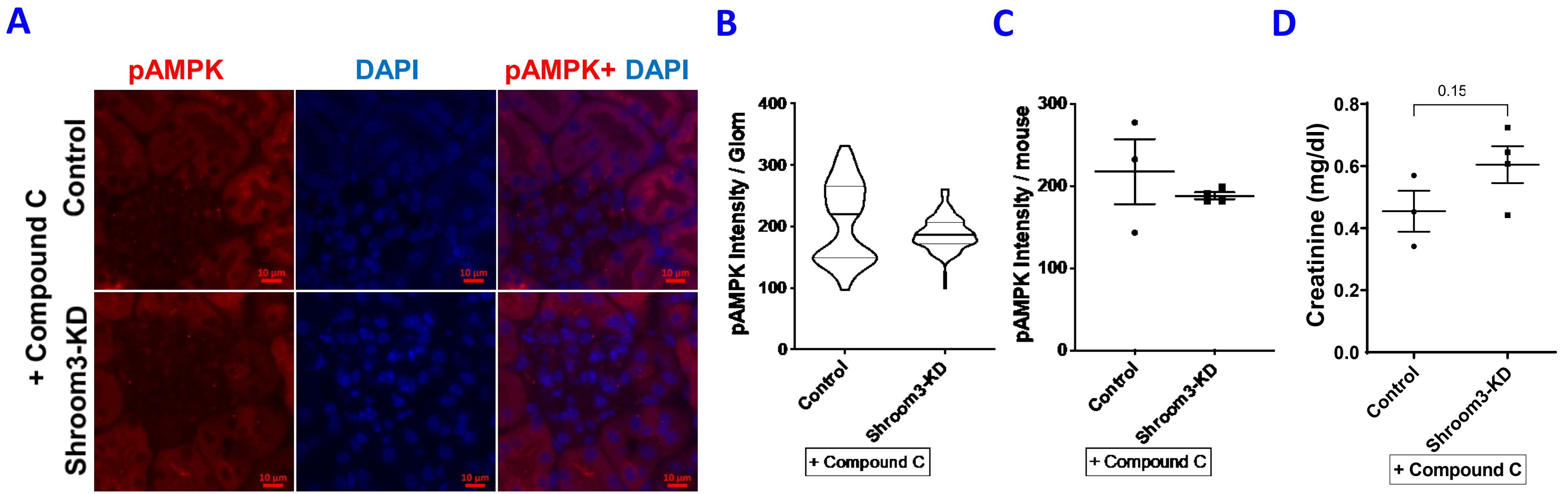

Figure S7. AMPK inhibition reverses Vglom reduction and promotes podocytopenia in Shroom3 knockdown mice: Control vs Shroom3-KD mice (~8 weeks) were DOX fed for 8 weeks and administered Compound C at week 5 **(7A)** Representative immunofluorescence images (40X) show Phosphorylated AMPK/DAPI in Control vs (upper row) & Shroom3-KD mice (lower row). **(7B)** Violin -plots show quantification of intensity of Phosphorylated AMPK/per glomerular outline per group (depicted per glomerulus; 30 gloms/animal). **(7C)** Dot plots quantify intensity of Phosphorylated AMPK/per glomerular outline per animal (n=3 vs 4), **(7D)** Dot plots compare Creatinine (mg/dl) levels in Control vs Shroom3-KD mice [Line/Whiskers=Mean/SEM; unpaired t-test].

Figure S8A-D

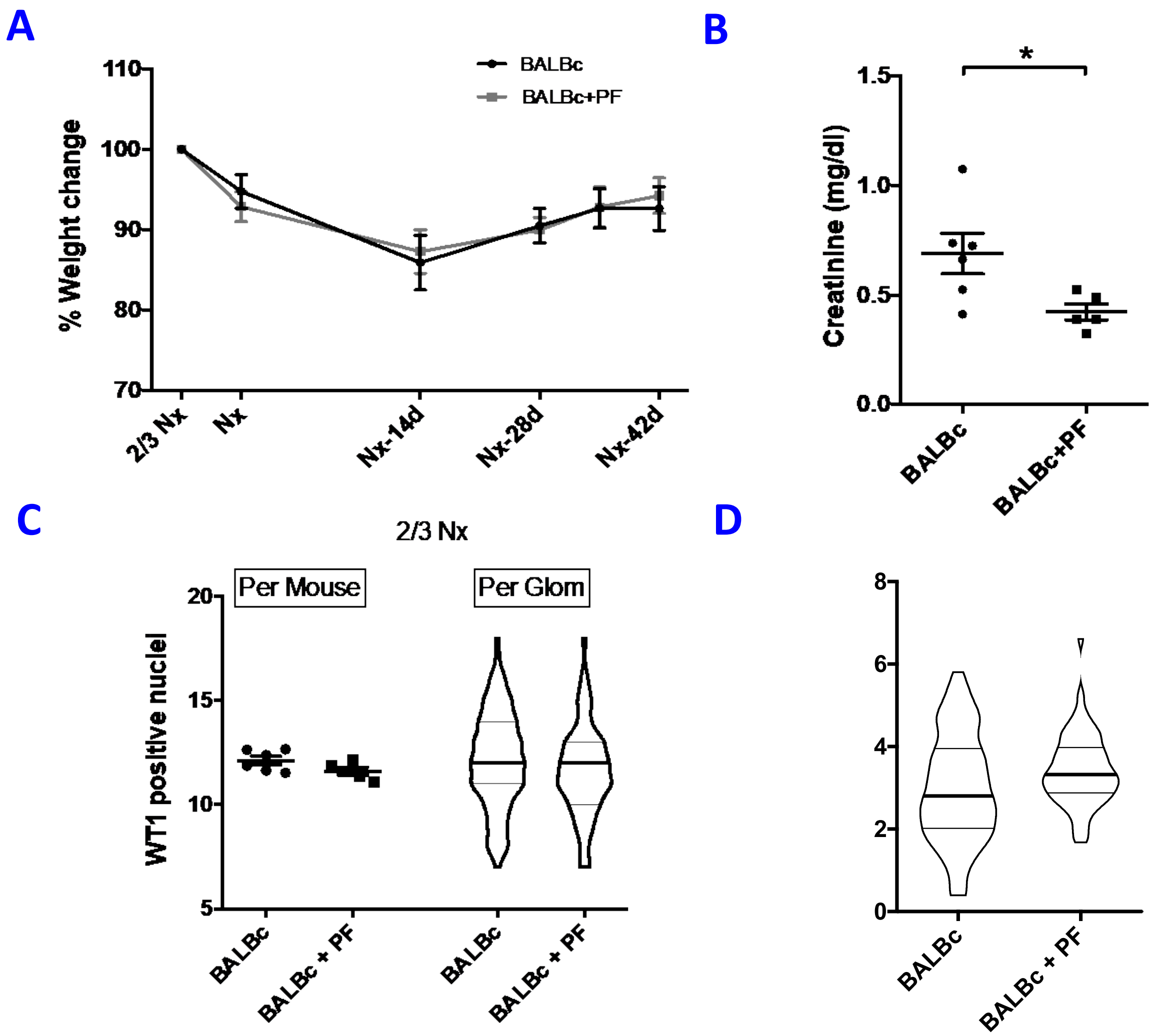

Figure S8. AMPK-activation reduces glomerular volume and preserves podocyte numbers in nephron loss-induced glomerular hypertrophy: Adult BALBc mice (near 8-wks) underwent 2/3rd nephrectomy (2/3 Nx), followed by contralateral nephrectomy (Nx) 1 week later, and were followed for 6 more weeks when the remaining kidney tissue (1/6th remnant) was harvested. Experimental animals were gavaged with AMPK-activator PF06409577 (3 doses/week; BALBc (n=6) vs BALBc+PF (n=5)). **(8A)** Lines show mean weight trends (as percentage of initial weight). **(8B)** Dot-plots compare Creatinine (mg/dl) in these groups at 6 weeks**. (8C)** Dot plots compare mean podocyte numbers/glomerulus/animal while Violin-plots (Line at median) depict distribution of mean podocytes/ glomerulus/ group (30 glomerular profiles/mouse) in 2/3 Nx kidneys obtained following first surgery. **(8D)** Violin-plots show distribution of podocyte nuclear density per glomerulus (per μm^3 Vglom) in each group (Line at median; n=10 glomeruli/mouse**)**. [Line/Whiskers = Mean/SEM; unpaired t-test; *= P<0.05; WT1= Wilm's Tumor-1 protein].

Figure S9A-D

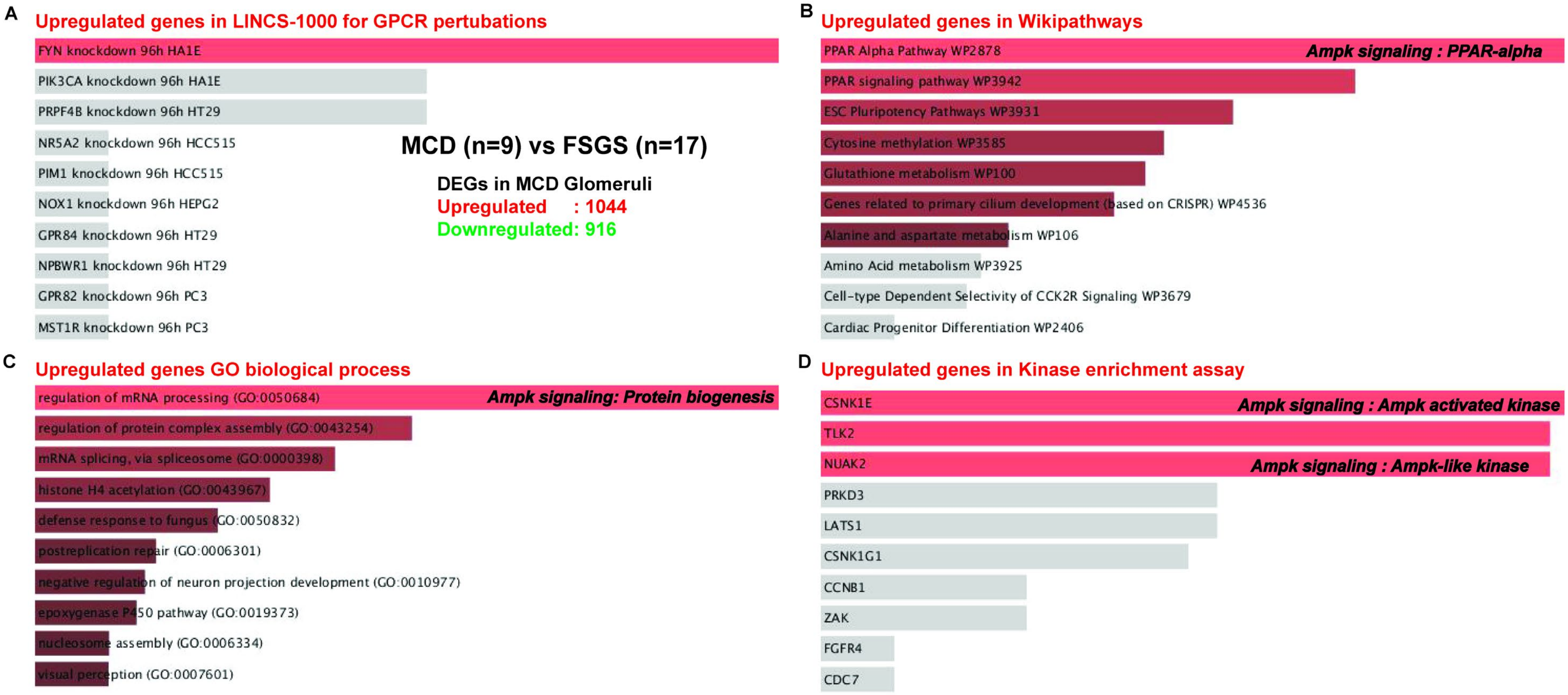

Figure S9. Transcriptomes of MCD vs FSGS reveals signatures of Fyn-kinase inactivation and Ampk-activation in MCD glomeruli: Glomerular transcriptomes of MCD (n=9) vs FSGS (n=17) cases from NEPTUNE cohort (dataset GSE68127) were analyzed for AMPK signaling using expression microarray data to identify significant DEGs (LIMMA test $P<0.05$). Significant upregulated DEGs were analyzed using enrichment platforms in ENRICHR as follows **(A)** LINCS-1000 G-protein coupled receptor perturbations, **(B)** Wikipathways**, (C)** GO-biological process and **(D)** Kinase enrichment assay. Top 10 results are ranked as bars by decreasing order of significance in each analyses (by P-value). Indicates $p<0.05$. Indicates $p>0.05$.

Figure S9E-H

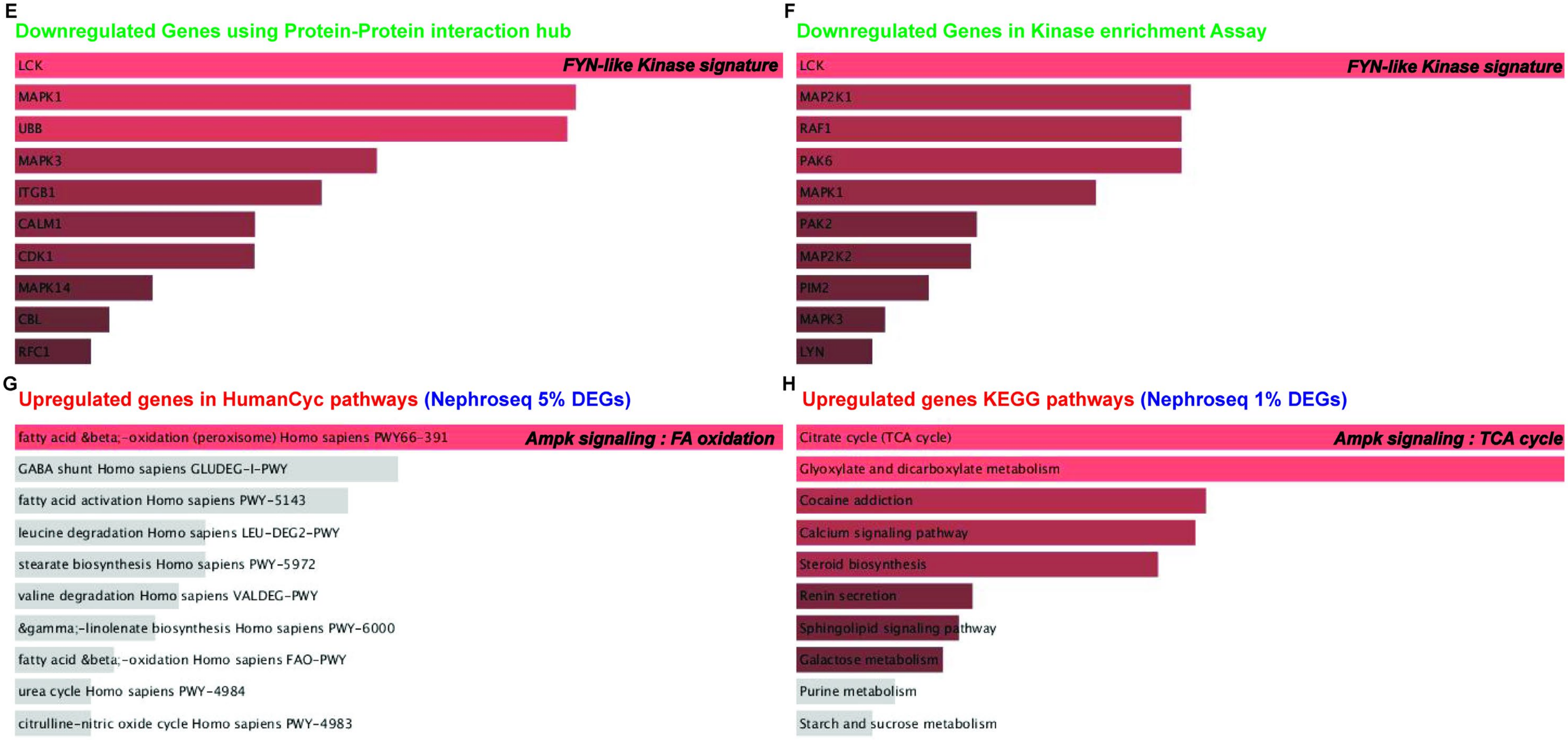

Figure S9. Transcriptomes of MCD vs FSGS reveals signatures of Fyn-kinase inactivation and Ampk-activation in MCD glomeruli (contd.): : Glomerular transcriptomes of MCD (n=9) vs FSGS (n=17) cases from NEPTUNE cohort (dataset GSE68127) were analyzed for AMPK signaling using expression microarray data to identify significant DEGs (LIMMA test $P<0.05$). Significant downregulated DEGs were analyzed using enrichment platforms in ENRICHR as follows **(E)** protein-protein interaction hub (STRING) **(F)** Kinase enrichment assay. Glomerular DEGS in MCD vs FSGS comparisons directly reported in Nephroseq database were analyzed similarly using ENRICHR. **(G)** Top 5% upregulated MCD DEGs showed enrichment of Beta oxidation of Fatty acids **(H)** while top 1% upregulated MCD DEGS identified enrichment of the TCA cycle. Top 10 results are ranked as bars by decreasing order of significance in each analyses (by P-value). Indicates p<0.05. Indicates p>0.05.

Supplementary Tables S1-2

Table S1A shows mean Vglom (μm^3) and Vglom components (normalized to a representative control animal) by Cavalieri method in Control vs Shroom3-KD mice (>10 Glomeruli/mouse, n=8)

Table S1B shows mean Vglom (μm^3) and Vglom components (normalized to a representative control animal) by Cavalieri method in Control vs Podocyte-Shroom3-KD mice (>10 Glomeruli/mouse, n=6 vs 5).

Table S2 shows mean Vglom (μm^3) and Vglom components (normalized to a representative control animal) by Cavalieri method in Control vs Shroom3-KD mice injected with Compound C (n=3 vs 4, respectively) used for AMPK inhibition studies.

Experiment Title	Glomerular volume (Vglom x 1000μm^3)			*Normalized volume Podocyte Component			*Normalized volume Mesangial component			*Normalized volume Capillary + Endothelial component		
	Control (Mean ± SEM)	Shroom3-KD (Mean ± SEM)	**p value	Control (Mean ± SEM)	Shroom3-KD (Mean ± SEM)	**p value	Control (Mean ± SEM)	Shroom3-KD (Mean ± SEM)	**p value	Control (Mean ± SEM)	Shroom3-KD (Mean ± SEM)	**p value
**TABLE S1A**												
Shroom3-KD (n=8 vs 8)	167.0 ± 5.8	128.4 ± 4.8	< 0.001	1.1 ± 0.1	0.9 ± 0.1	0.013	1.1 ± 0.1	1.0 ± 0.1	0.098	1.0 ± 0.1	0.9 ± 0.03	0.060
**TABLE S1B**												
Podocyte-Shroom3-KD (n=6 vs 5)	133.8 ± 6.3	107.8 ± 2.7	0.004	1.0 ± 0.0	0.8 ± 0.0	0.008	1.0 ± 0.0	1.0 ± 0.04	0.246	1.0 ± 0.1	0.8 ± 0.1	0.030
**TABLE S2**												
Compound C (n=3 vs 4)	113.7 ± 8.2	154.0 ± 11.3	0.044	1.0 ± 0.1	1.5 ± 0.0	0.057	1.0 ± 0.1	1.4 ± 0.3	0.228	1.0 ± 0.1	1.3 ± 0.1	0.114

* = Normalized to the mean of the control in each comparison
** = p values were obtained by Mann Whitney test

Supplementary Table S3

Table showing up- ( )and down-regulated ( ) genes in MCD to FSGS comparisons from the dataset GSE68127, NEPTUNE cohort.

Symbol	p	p.adj	Log2Rat
TMEM130	0.049937	0.64662	0.664134
PARD6A	0.049898	0.64662	0.727407
GPC6	0.04978	0.64656	0.66689
FXYD5	0.049775	0.64656	0.309322
LOC10050!	0.049696	0.646512	0.730653
PTDSS2	0.049611	0.646512	0.485209
PAM	0.049598	0.646512	0.260798
SUMO3	0.04941	0.646512	0.22884
CD53	0.049282	0.646512	0.171257
PAIP1	0.049173	0.646512	0.211177
PEX3	0.049144	0.646512	0.318623
HIST1H4J	0.048842	0.646172	0.322584
ALOX5AP	0.048788	0.646172	0.54565
RCAN1	0.048573	0.64502	0.510412
ANXA3	0.048466	0.644399	0.615571
MNDA	0.048425	0.644306	0.595985
RAB3B	0.048337	0.644019	0.669978
LOC10012!	0.048314	0.644019	0.265726
IL1RN	0.048311	0.644019	0.313273
IGHM	0.048276	0.644019	2.848967
TUBA4B	0.04818	0.644019	0.706305
MMP25	0.048166	0.644019	0.422245
FAM110B	0.048139	0.644019	0.333274
EXTL2	0.048131	0.644019	0.26814
OSMR	0.048047	0.644019	0.069113
CIAPIN1	0.047991	0.644019	0.283744
CRNDE	0.047965	0.644019	0.336241
CDH12	0.04793	0.644019	0.284133
PRR3	0.047928	0.643592	0.522912
CCL8	0.04792	0.644019	0.560407
PACSIN2	0.047753	0.643745	0.11724
MATK	0.047655	0.643391	0.50113
SYN1	0.047651	0.643391	0.378976
SCN1B	0.047577	0.643391	0.389584
CRELD1	0.047501	0.642792	0.161567
C10orf31	0.047193	0.640791	0.530133
ITGA5	0.047152	0.640783	0.384682
AOAH	0.047151	0.640783	0.433952
TAF5L	0.047117	0.640783	0.40806
ITLN2	0.047077	0.640783	0.478381
RAP1GAP2	0.047005	0.640783	0.567838
NLRC5	0.046913	0.640783	0.229957
SLC7A6	0.046834	0.640672	0.419061
RTN3	0.046784	0.640672	0.188754
GSDMD	0.046751	0.640672	0.964521
CDC20	0.046618	0.640323	0.757377

LY96	0.046586	0.640323	0.625324
PYGB	0.046541	0.640323	0.352968
PQLC3	0.046451	0.640134	0.272878
CCDC50	0.046307	0.639339	0.51486
GPR111	0.046261	0.639339	0.162138
HAS2	0.046222	0.639339	0.291673
ATP6V0E2	0.046193	0.639339	0.462937
BCR	0.046171	0.639339	0.132962
CCNA2	0.046116	0.639339	1.083348
MAP6	0.046083	0.639339	0.538697
SLC39A4	0.046	0.639339	0.364567
SLC35C1	0.045975	0.639339	0.379139
KDSR	0.045974	0.639339	0.356201
HAPLN3	0.045842	0.638993	0.400241
CMTM6	0.045773	0.638589	0.462198
LRP12	0.045748	0.638561	0.521099
EHBP1L1	0.045695	0.638561	0.51375
TNF	0.045686	0.636424	0.595463
ARF4	0.04564	0.638406	0.341147
CEP44	0.045554	0.63775	0.411796
HOXB2	0.045537	0.63775	0.202966
SNORA49	0.04544	0.637502	0.815987
PNMA3	0.045353	0.636614	0.229762
CUEDC1	0.045168	0.635325	0.460309
GTDC2	0.04511	0.63515	0.208532
SIL1	0.045095	0.63515	0.207258
ATP6AP1	0.044999	0.634962	0.398487
BEAN1	0.044992	0.634962	0.220165
IAH1	0.044876	0.633816	0.195669
ITSN2	0.044794	0.633218	0.196356
RPL23P8	0.044735	0.63266	0.427693
LOC64293	0.044696	0.63239	0.955073
TSPAN15	0.044674	0.63239	0.453195
IL1B	0.04464	0.63239	0.283901
VIM	0.044598	0.63239	0.226644
ALOX5	0.044566	0.63239	0.442307
PTPLA	0.044463	0.631972	0.253
AHDC1	0.044452	0.631972	0.261368
MIR3198-1	0.044423	0.631972	0.47727
IGKC	0.04435	0.631339	1.612673
NMRK2	0.044275	0.631171	0.269053
TNFAIP8	0.044257	0.631171	0.313598
LOC10028	0.044093	0.63055	0.288786
LINC00304	0.044037	0.630317	0.19498
LGALS9B	0.044029	0.630317	0.098925
PSD	0.044029	0.630317	0.414508
ZNF8	0.043991	0.630317	0.382747

FAM197Y1	0.043978	0.630317	0.205513
MFSD2A	0.04396	0.630317	0.332944
WRB	0.043959	0.630317	0.251489
SLC14A2	0.043912	0.630317	0.25158
C3	0.043836	0.630317	0.962548
PTPN18	0.043651	0.630317	0.17089
LRRC25	0.043519	0.629664	0.451618
STEAP3	0.043497	0.629664	1.058245
CCL3L3	0.043483	0.629664	0.411177
RPL27A	0.043409	0.629664	0.258134
RIPK3	0.043386	0.629664	0.23459
CENPE	0.043327	0.629664	0.443402
PLD4	0.043263	0.629664	0.177806
DUSP1	0.043261	0.629664	0.748328
C19orf59	0.043166	0.629664	0.304996
MARCO	0.043154	0.629664	0.339673
CHAC1	0.043131	0.629664	0.59377
VIMP	0.043037	0.629228	0.270836
ABCG4	0.043027	0.629228	0.326771
RSPRY1	0.042897	0.628616	0.285302
CHCHD5	0.042851	0.628516	0.365311
C19orf57	0.042704	0.62781	0.277381
IGHG3	0.042578	0.626521	0.53027
C1orf177	0.042544	0.626431	0.392515
POLR2L	0.042534	0.626431	0.156012
TOR1AIP2	0.042514	0.626431	0.373173
PLEK	0.04248	0.626431	0.956209
RNU4-7P	0.042438	0.626431	0.19608
DDX39A	0.042418	0.626431	0.480769
PAQR4	0.042246	0.625385	0.717923
HSPB8	0.042135	0.624314	0.210453
JOSD2	0.042057	0.623451	0.480719
SNORA71A	0.042051	0.623451	0.323285
GBP5	0.042048	0.623451	0.226488
EHD2	0.041917	0.623402	0.625741
CSRNP2	0.041741	0.621932	0.376282
UGCG	0.041637	0.62126	0.287376
TXNDC15	0.041597	0.621235	0.279522
NFKBIZ	0.041558	0.621232	0.664398
LOC10065	0.04153	0.621107	0.160745
C15orf39	0.041458	0.620311	0.421254
ANXA2	0.041443	0.620311	0.257919
STAP2	0.041313	0.619594	0.525556
TMEM41B	0.041201	0.618395	0.292946
RIOK3	0.041197	0.618395	0.23322
ULK4P3	0.041152	0.618395	0.301049
RHOBTB2	0.041149	0.618395	0.413675

CHPF	0.041138	0.618395	0.379639
PTPN2	0.041078	0.618395	0.260852
TRABD	0.040998	0.617884	0.447358
MIR27B	0.040997	0.617884	1.513017
C19orf33	0.040971	0.617884	0.608459
CBR1	0.040957	0.617884	0.160856
JMJD8	0.04092	0.617884	0.282802
FAM83H	0.04091	0.617884	0.503881
HCFC1R1	0.04085	0.617884	0.336282
AAK1	0.04071	0.617884	0.122711
LOXL1	0.040661	0.617884	0.297387
ARHGEF35	0.040638	0.617884	0.725666
SSC5D	0.040527	0.617884	0.592863
RNF24	0.040438	0.617766	0.620251
FAM155B	0.040295	0.61706	0.607168
STS	0.040257	0.61706	0.741637
KCNK17	0.040211	0.616994	0.343226
KAT2A	0.04021	0.616994	0.503261
LOC100500	0.04004	0.616345	0.003869
LOC100291	0.040029	0.616345	0.135043
TCF19	0.039957	0.616345	0.460917
RNASE6	0.039925	0.616345	0.921515
BHLHE40	0.039909	0.616345	0.517765
IQSEC3	0.039877	0.616345	0.46332
C10orf40	0.039866	0.616345	0.28856
RASSF5	0.03975	0.616196	0.370892
FXYD4	0.039704	0.616196	0.980441
LOC100499	0.039701	0.616196	0.466627
SCAP	0.039621	0.616196	0.075021
LOC100129	0.039595	0.616196	0.130182
TRADD	0.039581	0.616196	0.321755
C8orf56	0.039447	0.615953	0.336089
SNORD116	0.03942	0.615953	0.81054
RASGRP4	0.03938	0.615781	0.408694
TREM2	0.03935	0.615781	0.312036
PPP1R11	0.039307	0.61551	0.211413
CCDC61	0.039249	0.615497	0.41562
C1orf162	0.039234	0.615497	0.368464
RGS2	0.039137	0.615097	0.68866
OR14A16	0.039137	0.615097	0.333583
FLNB	0.039124	0.615097	0.478152
GUSBP5	0.039117	0.615097	0.293854
PTPRJ	0.039104	0.615097	0.213281
LOC100240	0.039095	0.615097	1.363215
KCNV2	0.039086	0.615097	0.273431
NME3	0.039024	0.615097	0.400198
CTF1	0.039013	0.615097	0.676461

PHF23	0.038924	0.615097	0.440692
LSM10	0.038833	0.614987	0.340481
EGR1	0.038733	0.614058	1.192184
ABLIM1	0.038733	0.614058	0.483608
CD8B	0.038719	0.614058	0.180215
RRP9	0.038679	0.614058	0.287404
ENAH	0.038667	0.614058	0.204065
AIDA	0.038664	0.614058	0.442591
KIF3C	0.038661	0.614058	0.316214
CTXN1	0.038639	0.614058	0.38153
RIPPLY1	0.038594	0.614058	0.779104
MESDC1	0.038547	0.614058	0.4242
ZDHHC8P1	0.038541	0.614058	0.324628
SAMHD1	0.038503	0.614058	0.350918
RAD1	0.038501	0.614058	0.299278
CHMP7	0.038252	0.614058	0.308493
SLITRK4	0.03821	0.614058	0.270701
TES	0.038203	0.614058	0.195051
REG1P	0.038132	0.613906	0.400265
ZNF71	0.038104	0.613773	0.720336
LOC10013(	0.038011	0.613773	0.204599
LPAR1	0.037944	0.613773	0.530986
YEATS2-AS	0.037883	0.613483	0.270637
TRBV2	0.037868	0.613483	0.272689
TRHDE	0.037832	0.613483	0.498005
PSMG4	0.037793	0.613483	0.360226
XAGE2	0.037777	0.613483	1.235137
IGHJ1	0.037699	0.613483	0.362244
DPYSL4	0.03769	0.613483	0.245648
NSUN5P1	0.037669	0.613483	0.150795
UBQLN3	0.037575	0.613483	0.303889
IRAK1	0.037465	0.613483	0.280413
C1QC	0.037434	0.613483	1.064488
LOC25357:	0.037423	0.613483	0.392744
MDK	0.037347	0.613483	0.348656
RNFT2	0.037297	0.613483	0.375254
MRPL41	0.037127	0.612208	0.248989
TSPY3	0.037066	0.611834	0.378624
LRRC61	0.037007	0.611175	0.456168
S100A14	0.036942	0.610559	0.301475
AP5M1	0.036901	0.610379	0.205749
LACTBL1	0.036896	0.610379	0.342784
YIF1B	0.036867	0.610379	0.292752
CCDC85B	0.036805	0.610379	0.443644
FAM60A	0.036802	0.610379	0.256773
LOC14651:	0.036706	0.61033	0.311646
CMTM4	0.036647	0.610272	0.687094

CCDC30	0.036629	0.610272	0.272626
KLF5	0.036582	0.610272	0.375326
ARRB2	0.036554	0.610272	0.546681
PSD4	0.036482	0.610272	0.410561
OR2AK2	0.03639	0.609582	0.196866
IL3RA	0.036359	0.609582	1.021581
PTPN7	0.036328	0.609582	0.602404
ENTPD2	0.036264	0.609136	0.916693
SMAGP	0.036214	0.609136	0.454024
ZDHHC20-/	0.03621	0.609136	0.536722
DKFZP434I	0.036174	0.609136	0.302947
C2orf69	0.036137	0.609136	0.428501
LAMC2	0.036036	0.609136	0.550744
MIR3143	0.036031	0.609136	0.705168
PARM1	0.035946	0.609063	0.428102
APOA1BP	0.035924	0.609063	0.239835
PAPPA2	0.035878	0.609063	0.429579
C6orf48	0.035864	0.609063	1.248914
CORO1C	0.03578	0.608838	0.317177
CABP2	0.035745	0.608838	0.374152
MIR4676	0.03562	0.608366	0.238242
LOC10013	0.035609	0.608366	0.262433
OSGEP	0.0356	0.608366	0.461928
C6orf47	0.035584	0.608366	0.658036
RASD1	0.035568	0.608366	1.042319
KLHL13	0.03554	0.608366	0.499784
KRTAP9-1	0.035486	0.608366	0.751258
SCGB3A1	0.035384	0.608237	0.441673
GALNT3	0.035348	0.607953	0.281014
ZNF469	0.035271	0.607926	0.589854
LINC00439	0.035084	0.607092	0.281638
SNORD115	0.034951	0.606405	0.375874
URB1	0.034861	0.606405	0.278297
SLC38A8	0.034861	0.606405	0.430086
GARS	0.034785	0.606405	0.248505
FMO6P	0.034758	0.606405	0.206662
KRT18P10	0.034451	0.606405	1.045792
PIWIL3	0.03444	0.606405	0.411362
TSSC1	0.034169	0.603232	0.313044
EIF4G3	0.033909	0.601307	0.151199
SSR2	0.033866	0.600879	0.102921
PIN1	0.033836	0.600666	0.252665
EDEM2	0.033831	0.600666	0.224076
PAX9	0.033774	0.600666	0.417637
WFDC1	0.033717	0.600666	0.599628
PLP2	0.033647	0.600666	0.113205
ABCC10	0.033615	0.600666	0.402733

KDELR3	0.033613	0.600666	1.300786
CSRNP1	0.033575	0.600666	0.836221
RASGEF1A	0.033473	0.600263	0.593751
CCDC36	0.033342	0.599225	0.340114
NDST1	0.033047	0.595898	0.276021
MGAT2	0.033036	0.595898	0.302043
ATP13A2	0.033015	0.595898	0.437942
MLLT1	0.033	0.595898	0.243088
FAM211A	0.032972	0.595898	0.210548
C18orf56	0.03296	0.595898	0.749915
RNU6-69	0.032913	0.595898	0.326121
CCT8L2	0.032885	0.595898	0.25873
FMO2	0.03283	0.595898	0.800424
TPGS2	0.032696	0.595898	0.293916
CDC27	0.032647	0.595898	0.521334
GNG5P2	0.032588	0.595898	0.439542
LOC72767	0.032579	0.595898	0.30495
ZCCHC10	0.032489	0.595898	0.215255
PKD2	0.032245	0.594539	0.308614
KBTBD8	0.032174	0.59357	0.430993
HEATR2	0.032093	0.592754	0.331275
CCDC70	0.032085	0.592754	0.257275
SFRP2	0.032034	0.592754	1.351151
LOC40139	0.031962	0.592754	0.26969
LGALS1	0.031915	0.592754	0.405489
SYAP1	0.031846	0.592754	0.109451
SNORD10	0.03163	0.591857	0.799539
MIR606	0.031589	0.59165	0.272386
SLC35E1	0.031534	0.59165	0.220248
SPINT1	0.031494	0.59126	0.476946
SRGN	0.031336	0.590372	0.503096
PPP2R1B	0.031259	0.589264	0.325636
LOC33879	0.031187	0.588944	0.342653
SPPL2A	0.031091	0.587818	0.429585
APOF	0.031075	0.587818	0.265117
FOS	0.031063	0.587818	0.581319
DOK5	0.031008	0.587818	0.509009
CKS2	0.030991	0.587818	1.537652
C1orf216	0.030976	0.587818	0.478796
TRBV6-1	0.030938	0.587818	0.203625
CHCHD1	0.030864	0.587818	0.388733
LSM2	0.030853	0.587818	0.436919
COLEC12	0.030847	0.587818	0.890556
ZYX	0.030829	0.587818	0.305417
RBM38	0.030818	0.587818	0.444533
PRR11	0.030755	0.587818	0.299837
ASRGL1	0.030652	0.587818	0.41109

ZMAT3	0.030519	0.587818	0.762282
WASF1	0.030497	0.587818	0.251002
UEVLD	0.030466	0.587818	0.274054
CASP8	0.030402	0.587327	0.264902
KIF5A	0.030398	0.587327	0.208363
RPS21	0.030363	0.587327	0.218113
EOGT	0.030314	0.587327	0.369608
TNFAIP2	0.03023	0.587327	0.40521
PDK3	0.030201	0.587327	0.369842
VARS	0.030169	0.586225	0.268665
TP53INP2	0.030157	0.587327	0.415077
ZNF542	0.030123	0.587327	0.534347
CYP51A1	0.030111	0.587327	0.43013
EHF	0.030076	0.587327	0.393854
NF2	0.030071	0.587327	0.339476
GLTP	0.030063	0.587327	0.30545
SENP5	0.03003	0.587327	0.315985
RHD	0.030028	0.587327	0.728128
S100A8	0.029955	0.587327	0.21845
F8A1	0.029926	0.587327	0.242726
MRPS18A	0.0298	0.586639	0.250852
B3GNT2	0.029729	0.586639	0.292044
NKX1-1	0.029665	0.586639	0.314002
MAPK1IP1	0.029367	0.585697	0.34619
MIR548AL	0.029358	0.585697	0.64245
DNASE2	0.029307	0.585234	0.957236
SCRN1	0.029264	0.585097	0.404353
FAM181B	0.029127	0.584338	0.59322
C16orf82	0.029108	0.584338	0.25203
ZNF226	0.028963	0.583344	0.506423
ATRNL1	0.028951	0.583344	0.469187
FASN	0.028884	0.583344	0.17027
SYK	0.028848	0.583344	0.88911
KCNJ14	0.028846	0.583344	0.337179
ETV5	0.028834	0.583344	0.531094
HLA-DRA	0.028786	0.564193	0.374363
MIR1266	0.028643	0.583344	0.244823
ITPK1	0.028551	0.583344	0.446776
HAUS1	0.028523	0.583344	0.495293
ST8SIA6	0.028405	0.583344	0.857427
C9orf9	0.028328	0.583344	0.358398
RNPEPL1	0.028306	0.583344	0.410532
PCDHB8	0.028256	0.583344	0.47499
NUPR1L	0.028224	0.583344	0.37882
PERP	0.028207	0.583344	0.454755
GRK7	0.028172	0.583344	0.314502
MTND2P2[illegible]	0.028163	0.583344	0.362917

GORAB	0.028129	0.583344	0.294123
C1orf85	0.028011	0.583344	0.360464
ZP3	0.027969	0.583344	0.365682
MT1M	0.027962	0.583344	0.329686
RN5S28	0.02791	0.583344	0.367961
GOLGA8E	0.027867	0.583344	0.436112
SNORD116	0.027858	0.583344	0.797737
TUBB2B	0.027737	0.583344	0.708562
CGB1	0.02771	0.583344	0.480575
JTB	0.027638	0.582813	0.174954
DUSP15	0.027618	0.582813	0.234097
KANSL1-AS	0.02759	0.582813	0.537147
LCA10	0.027589	0.582813	0.592288
JUNB	0.027581	0.582813	0.405141
CXCL9	0.02754	0.582813	0.703385
C16orf45	0.027529	0.582813	0.636627
LOC10028	0.027508	0.582813	0.380135
HOXA7	0.027477	0.582813	0.314707
MS4A6A	0.027458	0.582813	0.795207
LYPD1	0.027398	0.582813	0.855592
KIAA1609	0.027323	0.582813	0.091795
TRAF7	0.027313	0.582813	0.217378
SHISA8	0.027271	0.582813	0.368089
TISP43	0.027224	0.582813	0.704388
MS4A7	0.027181	0.582813	0.558003
SH3GL1	0.027147	0.582813	0.436525
DNAJB5	0.027077	0.582813	0.447292
MTA2	0.027046	0.582756	0.309953
PMP22	0.027013	0.582756	0.371531
SMCR8	0.026949	0.582101	0.106793
RNU105B	0.026869	0.581178	0.712066
P2RY13	0.02685	0.581178	0.254074
SLC35F5	0.02683	0.581178	0.302117
RNU5B-1	0.026776	0.580717	0.783535
NARF	0.026755	0.580656	0.338544
LOC10029	0.026622	0.579354	0.728032
ACLY	0.02649	0.57766	0.141493
RASSF10	0.026466	0.57753	0.508214
KRT8	0.026385	0.576318	0.51347
ELOVL5	0.026326	0.576029	0.375055
PUSL1	0.026218	0.575258	0.342015
PLXNA3	0.02621	0.575258	0.597522
C4orf48	0.026135	0.575258	0.446362
S1PR5	0.02611	0.575258	0.287546
C1orf122	0.025925	0.57371	0.331954
LOC40124	0.025918	0.57371	0.207
C16orf89	0.02586	0.573698	0.392204

DIRC2	0.025766	0.573583	0.352885
ID2B	0.025752	0.573583	0.257262
MSC	0.025559	0.57137	0.28284
ST8SIA5	0.025555	0.57137	0.297721
NETO1	0.025532	0.57137	0.793711
SNRPN	0.025465	0.56676	0.075477
PARP12	0.025405	0.57137	0.520985
ODF4	0.025365	0.57137	0.507072
MIR1275	0.025335	0.57137	0.302304
STIM1	0.025204	0.5694	0.50898
ADAMTS2	0.025138	0.569123	0.756498
SDC4	0.025112	0.569123	0.367206
BRD2	0.025028	0.569042	0.611354
FAM101B	0.024921	0.568633	0.446884
ZP1	0.024897	0.568633	0.2627
TGFB1	0.024736	0.56612	0.391811
LOC10013:	0.024669	0.565964	0.786948
SSBP4	0.024658	0.565964	0.389575
CFB	0.024655	0.565964	0.577267
TMSB15A	0.024604	0.565964	0.842259
C6orf120	0.024594	0.565964	0.425655
TIMM8A	0.024575	0.565964	0.867759
PLEKHB1	0.024565	0.565964	0.24893
ELOVL6	0.024556	0.565964	0.306616
CD14	0.024496	0.565964	0.634756
ITPKA	0.024467	0.565964	0.333273
SREBF2	0.024426	0.565964	0.319894
CDKN1A	0.024406	0.565964	0.499898
PHLDA2	0.024391	0.565964	0.492431
GPR6	0.024287	0.565964	0.367794
SNHG7	0.024169	0.565594	0.282274
TRIM10	0.024154	0.559171	0.305598
AEBP1	0.024147	0.565498	0.355444
PRKAB1	0.024111	0.56506	0.181305
CCNA1	0.02404	0.564214	0.25083
AADACL2	0.02397	0.563541	0.117034
NUAK1	0.023784	0.561106	0.400441
IL18	0.023784	0.561106	0.838016
KIAA1984	0.023751	0.561106	0.233645
OST4	0.023739	0.561106	0.243379
FBXL8	0.023631	0.561106	0.330348
CPOX	0.0236	0.561106	0.70068
ANK1	0.023566	0.561106	0.217211
HAUS2	0.023396	0.561106	0.660727
RAB39A	0.023356	0.561106	0.355168
ECEL1	0.023327	0.561106	0.223553
BCL2A1	0.023308	0.561106	0.598501

CORO1A	0.023297	0.561106	0.934018
COL8A1	0.023294	0.561106	0.67009
MTHFD1L	0.023265	0.561106	0.489938
TMEM211	0.023256	0.561106	0.258041
MIR127	0.023105	0.561106	0.424209
GPR143	0.023039	0.561106	0.378951
DDX21	0.022985	0.561106	0.429831
C5AR1	0.022894	0.561106	0.997565
C1orf88	0.022888	0.561106	0.417127
TRMT1	0.022871	0.561106	0.395461
GBA	0.02276	0.56007	0.360014
NFKBIE	0.022719	0.559935	0.871719
TMEM230	0.022675	0.559259	0.316139
CLC	0.022638	0.558857	1.095709
SLC35B2	0.022628	0.558857	0.331715
MOXD1	0.022622	0.558857	0.581705
G6PC3	0.02256	0.558578	0.541695
CD68	0.022546	0.558578	0.318481
CNP	0.022532	0.558578	0.476226
RMRP	0.022511	0.558578	0.211863
C9orf16	0.022411	0.558578	0.482107
LOC10013:	0.022398	0.558578	0.444032
LOC64136	0.022354	0.558578	0.342217
DNAJC25	0.022323	0.558578	0.322179
GABRA2	0.022306	0.558578	0.706437
C12orf75	0.022304	0.558578	0.665931
KIF1A	0.0223	0.558578	0.667781
ZNF212	0.022262	0.558578	0.423713
OSTM1	0.02224	0.558578	0.256062
SPI1	0.022234	0.558578	0.360219
BIRC5	0.022151	0.558578	0.370364
ABCA7	0.022095	0.558578	0.399047
PPP2R5A	0.022004	0.558578	0.530203
PRSS23	0.022004	0.558578	0.572378
RN5S240	0.021995	0.558578	0.54171
LRRC16A	0.02199	0.558578	0.333204
SIGLEC10	0.021955	0.558578	0.708023
MIR551B	0.021817	0.558578	0.524387
ETHE1	0.02181	0.558578	0.254958
LOC10018	0.021727	0.558578	0.399703
HS6ST2	0.021727	0.558578	0.567564
DEFB122	0.021703	0.558578	0.293078
MIR4486	0.02158	0.558578	0.243366
CASKIN1	0.021566	0.558578	0.204508
ADPGK	0.021566	0.558578	0.659725
RET	0.021516	0.558578	0.702437
RN5S53	0.021465	0.558578	0.627251

LBH	0.021339	0.557352	0.416413
RNU7-23P	0.0213	0.557352	0.306669
FCER1G	0.021243	0.557018	0.937161
TMEM97	0.0211	0.555553	0.308726
DDB2	0.02106	0.554961	0.330444
CST6	0.021012	0.554595	1.434322
MNAT1	0.021003	0.554595	0.144316
ARHGEF5	0.020996	0.554595	0.560287
HLA-DMA	0.020908	0.536832	0.346256
P2RX5-TAX	0.020835	0.554444	0.4328
C1QTNF9B	0.020818	0.554444	0.495275
C15orf41	0.020779	0.554444	0.440508
RNASE2	0.020775	0.554444	1.504676
DLGAP4	0.020739	0.554444	0.262228
CCL4L2	0.020651	0.554444	1.510221
NRN1L	0.020646	0.554444	0.22384
KCNN3	0.020626	0.554444	0.686941
ERGIC1	0.020609	0.554444	0.288879
PITPNM3	0.020528	0.554444	1.051111
SCARB1	0.020467	0.554444	0.269521
ATG9B	0.02041	0.554444	0.390308
LOC60672	0.020386	0.554444	0.407837
ASB6	0.020241	0.552944	0.467537
SST	0.019959	0.548572	1.120955
ZAP70	0.019923	0.548572	0.315191
SUMF2	0.019838	0.548572	0.331862
LINC00578	0.019824	0.548572	0.297472
NLE1	0.019647	0.546059	0.248613
LST1	0.019604	0.541584	0.562811
MYOF	0.019575	0.545805	0.410191
TYMS	0.019474	0.544091	1.608595
MNF1	0.019401	0.543476	0.336953
MRP63	0.019392	0.543476	0.232635
HOXB6	0.01937	0.543476	0.205741
RPL13	0.019272	0.542696	0.243564
SLX1B-SUL	0.019214	0.542029	0.559608
FAM49A	0.019189	0.542029	0.792555
PYCARD	0.019039	0.540896	0.590277
C4orf47	0.019007	0.54048	0.330008
SEZ6L2	0.018968	0.5402	0.537361
VSIG4	0.01896	0.5402	0.692296
ISX	0.018928	0.540139	1.509816
SLC6A15	0.018772	0.538085	0.146675
GADD45GI	0.018679	0.535915	0.347074
LYVE1	0.018656	0.535915	1.372656
LOC12468	0.018614	0.535915	0.258453
TBC1D24	0.018613	0.535915	0.284518

MXRA5	0.018595	0.535915	1.320335
MFSD3	0.018581	0.535915	0.389149
RFC2	0.018556	0.535915	0.433399
PGLS	0.018516	0.535915	0.571988
MTM1	0.018432	0.535915	0.229716
LCLAT1	0.018378	0.534946	0.38261
ST3GAL2	0.018329	0.534946	0.50234
UBE2C	0.018236	0.534706	0.698685
LOC28464	0.018185	0.534582	0.219972
ARL4C	0.018078	0.533456	0.859873
RASA4	0.018073	0.50749	0.935814
MAB21L1	0.018065	0.533456	0.326109
KLHDC7B	0.018013	0.533456	0.199605
HPCAL4	0.018013	0.533456	0.558315
MFSD10	0.018007	0.533456	0.490071
CARD16	0.017933	0.533456	0.436242
EPHA2	0.017896	0.533045	0.684436
RNF26	0.017876	0.532959	0.421867
RAX	0.017744	0.531678	0.41115
CDC34	0.017731	0.531678	0.695154
MAP2K1	0.017711	0.531678	0.557201
GALNT6	0.017615	0.531611	0.561454
S100A9	0.017568	0.53069	1.068247
SAT1	0.017567	0.53069	0.234691
WDR24	0.017524	0.53069	0.191321
LILRB4	0.017519	0.53069	0.40813
C1orf233	0.017507	0.53069	0.404019
POC1A	0.017489	0.53069	0.514163
PNMA2	0.017437	0.53069	0.409395
MAP2K3	0.017425	0.53069	0.493863
GAPDH	0.017411	0.53069	0.259079
SEC23A	0.01739	0.53069	0.294415
SLC7A14	0.017381	0.53069	0.794877
VPS37C	0.01738	0.53069	0.318333
COMT	0.017361	0.53069	0.241744
CDC42EP1	0.017169	0.53069	0.219287
LOC10050	0.017168	0.53069	0.284024
ARNTL2	0.017156	0.53069	0.432429
GPR89A	0.016989	0.53069	0.397622
C7	0.016827	0.53069	0.94347
PRIC285	0.0168	0.53069	0.636416
INSL4	0.016774	0.53069	0.37158
IQCF2	0.016675	0.53069	0.200907
TAF10	0.01662	0.53069	0.302967
LOC10029	0.016613	0.53069	2.781762
LY86	0.016601	0.53069	0.887031
NRG2	0.016598	0.53069	0.399839

IGLV2-18	0.016594	0.53069	1.521025
LOC72789(	0.016558	0.53069	0.550117
BRI3BP	0.016495	0.53069	0.428043
TNFRSF1B	0.016324	0.53069	0.557282
ACTN3	0.016255	0.53008	0.489309
LGALS3	0.016241	0.53008	0.491861
MYO1F	0.016204	0.530015	0.638026
HEXA	0.016198	0.530015	0.248378
RHBDD2	0.016094	0.528606	0.302697
PDDC1	0.016031	0.528606	0.521236
NXF5	0.015895	0.52801	0.542411
KRT18P49	0.01589	0.52801	0.302963
HCK	0.015886	0.52801	0.702312
PHF11	0.015761	0.527626	0.328226
TARP	0.015729	0.527626	0.347448
LRP10	0.015728	0.527626	0.45834
ZFYVE27	0.015722	0.527626	0.545286
SLC30A7	0.01572	0.527626	0.350613
KRTAP1-5	0.015667	0.527626	0.365108
GPR176	0.015634	0.527626	0.382593
TWISTNB	0.015581	0.527626	0.357332
PLAU	0.015578	0.527626	0.596709
ZFP36L1	0.015534	0.527626	0.210067
PTPRC	0.015451	0.527626	0.66236
THSD4	0.015432	0.527626	0.253828
PRKAG1	0.015399	0.527626	0.260624
OXCT2	0.015317	0.52686	0.338588
PRF1	0.015157	0.52576	0.961318
PILRA	0.015073	0.524105	1.130736
DIAPH3	0.015046	0.524105	0.549299
FADS1	0.015011	0.524105	0.347095
LDLR	0.015	0.524105	0.849231
GUK1	0.01498	0.524105	0.340147
APOBR	0.014795	0.521889	0.213667
NFIB	0.01479	0.521889	0.411751
KRT18P54	0.01469	0.521557	0.569027
FST	0.01458	0.518243	0.174875
IGHV1OR1	0.014481	0.517282	0.478167
GMIP	0.014437	0.517282	0.364526
SNORA79	0.014371	0.516534	0.554702
TGFB3	0.014314	0.516534	0.291533
CCL2	0.014208	0.516329	0.867088
ADORA2A	0.014156	0.515832	0.355043
CD300LB	0.014077	0.515455	0.358662
BIN2	0.01402	0.515455	0.415545
PITPNM1	0.01401	0.515455	0.635581
ANXA2P2	0.013907	0.515455	0.349008

ICAM4	0.013897	0.515455	0.831085
FAM57A	0.013822	0.514441	0.271042
GATSL1	0.013803	0.514441	0.525531
CAP2	0.013747	0.51433	0.314427
SYT11	0.013728	0.514243	0.473296
RNU1-13P	0.013717	0.514243	0.44269
MIR4640	0.013708	0.458785	0.272277
TOR4A	0.01366	0.514087	0.582319
FCN1	0.013657	0.514087	0.659509
BAI2	0.013588	0.514075	0.308613
MICB	0.013578	0.465661	0.523577
UBE2S	0.013385	0.51216	0.673462
NELF	0.013375	0.51216	0.624493
KLHL7-AS1	0.013317	0.511427	0.476193
SYP-AS1	0.013283	0.510707	0.480521
CSF1R	0.013237	0.509741	0.949812
ETV1	0.01322	0.509741	0.58384
MMP23A	0.013217	0.509741	0.809267
ZFP36L2	0.013123	0.509741	0.386896
ICAM1	0.013089	0.509741	0.595939
PPTC7	0.013028	0.509741	0.443757
PLCB2	0.013008	0.509741	0.458033
NUDT4P1	0.012774	0.508889	0.913985
CHEK1	0.012738	0.508509	0.408894
UCKL1-AS1	0.012686	0.508509	0.366153
CD9	0.012628	0.508509	0.256331
TFAP2A	0.012611	0.508509	0.369258
RNF144B	0.012569	0.443885	1.628391
KIF14	0.012475	0.507713	0.322121
RRP36	0.012416	0.507713	0.473738
SPATA18	0.012347	0.507685	1.01923
TMEM159	0.012337	0.507685	0.429925
MYC	0.012264	0.506054	0.517655
SSR3	0.012179	0.504649	0.356072
ORAI1	0.01217	0.504649	0.614387
IMMP1L	0.01216	0.504649	0.470551
ANXA1	0.01213	0.504649	0.614187
AVL9	0.012124	0.504649	0.45555
MAZ	0.012123	0.504649	0.469266
C22orf40	0.012123	0.504649	0.444122
MAPK1	0.012035	0.504649	0.369486
SRSF6	0.012032	0.504649	0.534239
IGHG2	0.012018	0.504649	0.657338
HMMR	0.012002	0.504649	0.64697
SYTL3	0.011959	0.504649	0.451475
KIR3DL2	0.011916	0.504649	0.699678
LOC10065	0.01185	0.504649	1.249444

MARCKS	0.011785	0.504649	0.30341
SNN	0.011757	0.504649	0.575462
VAMP7	0.011721	0.456006	0.450634
TSKU	0.011684	0.504649	0.538468
PDIA4	0.011654	0.504649	0.239176
FSTL3	0.011654	0.504649	0.70819
LOC10050(	0.011625	0.504649	0.310243
SLC38A1	0.011335	0.500203	0.85409
CHPF2	0.011283	0.499935	0.486767
CBFA2T3	0.011218	0.498715	0.688838
HLA-DQB1	0.011182	0.498205	0.731931
GNG12	0.011172	0.498205	0.212536
LRFN3	0.011146	0.497999	0.674851
HBE1	0.01109	0.496863	0.416356
NAT6	0.011079	0.496863	0.418261
IGFBP6	0.011046	0.496863	0.792354
ACTRT3	0.01103	0.496863	0.48483
TSPAN6	0.01101	0.496863	0.254347
ZNF747	0.01097	0.496863	0.467772
PROCR	0.010877	0.494951	0.690524
PTGES2	0.010718	0.489393	0.621848
CPSF3L	0.010685	0.489002	0.470396
ECSCR	0.01065	0.488508	0.71293
CX3CL1	0.010648	0.488508	0.599493
LOC15786(	0.010519	0.486362	0.361773
IGSF6	0.010474	0.485616	0.906055
TMEM183	0.010369	0.484688	0.47357
LOC10050!	0.010294	0.484688	0.600579
ZNF826P	0.010279	0.484688	0.995245
SENP3-EIF4	0.010272	0.484688	0.340821
FAM176B	0.010185	0.48444	0.541121
DUSP6	0.010154	0.48444	0.476381
MRPL43	0.010115	0.484418	0.32302
PROS1	0.010113	0.484418	0.332349
HLA-DMB	0.010082	0.444105	0.456118
UBL7	0.010005	0.483486	0.386996
AGPAT2	0.00995	0.482383	0.53728
IMPDH1	0.009924	0.482383	0.218165
TNFRSF9	0.009908	0.482383	0.339087
GLIPR2	0.009898	0.482383	0.769748
SCUBE3	0.009895	0.482383	0.725241
MIR571	0.009865	0.482383	0.458881
MLLT11	0.009824	0.482383	0.686288
ERMN	0.009794	0.482383	0.368419
ALKBH5	0.009775	0.482383	0.396192
PGM2L1	0.009767	0.482383	0.550646
UNKL	0.009684	0.481806	0.375904

C20orf112	0.009683	0.481806	0.340508
LOC55011:	0.009645	0.481806	0.269159
LDHA	0.009599	0.480612	0.308809
APOBEC3A	0.009562	0.480206	0.68737
TMEM191	0.009516	0.478634	0.672284
TSPO	0.009442	0.478634	0.416736
NCBP2-AS:	0.00941	0.478634	0.535591
ORMDL2	0.009354	0.478634	0.538352
CYorf17	0.009331	0.47838	0.407711
BACE2	0.009289	0.477762	0.805853
MIR744	0.00918	0.476879	0.354011
SELRC1	0.009166	0.476879	0.454653
SLX1A-SUL	0.009147	0.476879	0.624538
ADAM8	0.009052	0.476879	0.352213
PCDHA11	0.009034	0.476879	0.275127
MEGF10	0.008945	0.476879	0.571886
CMTM3	0.008894	0.476879	0.294239
MIR597	0.00888	0.476879	0.68456
FBXO36	0.008874	0.476879	0.74923
KIAA0100	0.008834	0.476879	0.177175
C20orf111	0.008819	0.476879	0.391689
SPCS3	0.008782	0.476879	0.39924
LOC10028	0.008741	0.476879	0.385438
JPH1	0.008656	0.475758	0.675835
LMCD1	0.00865	0.475758	0.497272
TNFRSF14	0.008502	0.472176	0.331508
NOC4L	0.008496	0.472176	0.325205
ASPHD1	0.008478	0.472176	0.769091
TICAM1	0.008418	0.470806	0.670539
NPAS1	0.00832	0.470268	0.472045
HIST1H3E	0.008311	0.470268	0.811647
C9orf91	0.008286	0.469978	0.53221
MCTP1	0.008282	0.469978	0.533579
CAD	0.008264	0.469978	0.495694
ADCY5	0.00822	0.469595	0.500482
FOSL1	0.008187	0.469371	0.750485
SIRT5	0.008165	0.468913	0.60708
CD99P1	0.008124	0.468273	0.615518
ZNF280B	0.008119	0.468273	0.452992
GABRE	0.00807	0.466839	0.470944
C1QA	0.008025	0.466775	0.534708
KRT19	0.007895	0.464252	0.939659
ECT2	0.007885	0.464252	0.650867
SFRP1	0.007824	0.463511	0.889895
SERPINE1	0.007772	0.462977	1.605955
HIST1H3I	0.007757	0.462961	1.519243
RHOG	0.00774	0.462827	0.356387

AP4E1	0.00774	0.462827	0.499622
POLE4	0.007702	0.462827	0.321354
SNORD32E	0.007637	0.460963	0.430417
GZMA	0.007636	0.460963	1.558192
LIPM	0.007635	0.460963	0.291459
FAM153B	0.007618	0.460963	0.662517
YJEFN3	0.007551	0.460963	0.467283
KIAA1755	0.007539	0.460963	0.171848
NGEF	0.007523	0.460963	0.660874
ECM1	0.007416	0.458887	1.060478
GNAO1	0.007413	0.458887	1.173772
SLC25A46	0.007331	0.456008	0.348741
MIR497HG	0.007293	0.455648	0.355225
ZFP36	0.007198	0.450585	0.355571
GADD45G	0.007167	0.450423	0.619264
FCHO1	0.007166	0.450423	0.482536
YIF1A	0.007144	0.450423	0.501202
GDE1	0.007123	0.450347	0.463856
LOC10012!	0.007098	0.449663	0.629842
IQSEC2	0.00709	0.449663	0.63315
PRAF2	0.007085	0.449663	0.52788
SH3BGRL3	0.007022	0.448399	0.335401
C7orf43	0.007006	0.448399	0.584154
MLX	0.006994	0.448399	0.451007
CRISPLD2	0.006931	0.448399	1.056192
C17orf56	0.006926	0.448399	0.488259
LOC10065:	0.006825	0.44805	0.483043
MUC3A	0.006787	0.448026	0.333607
LRRC20	0.00678	0.448026	0.408146
TXNL4A	0.006703	0.445758	0.306959
SNORA42	0.006647	0.444841	0.922734
RPS2P32	0.006612	0.444335	0.753114
TLR1	0.006584	0.444333	0.343616
GRINA	0.006562	0.443821	0.405995
GIPC1	0.006414	0.43842	0.377131
RHOV	0.006399	0.438365	0.371882
TRIB1	0.006375	0.437655	0.654354
INHBB	0.006299	0.436719	0.448648
SCO2	0.006236	0.434631	0.220032
SYNGR2	0.00623	0.434631	0.686547
AXL	0.006226	0.434631	0.864374
AQP3	0.006214	0.434631	0.620446
CYBB	0.006154	0.434631	0.633244
FLJ25917	0.006128	0.434631	0.295445
SPTSSB	0.006077	0.434631	0.563325
ELOVL7	0.006058	0.434631	0.386485
KIAA0040	0.005992	0.433102	0.573491

KIAA1161	0.005939	0.433778	0.663591
ANXA2P3	0.005921	0.433661	0.951933
TP53I13	0.00592	0.433661	0.639387
TNXB	0.005915	0.411503	0.609995
UBTD2	0.00588	0.433661	0.601387
CDYL2	0.005757	0.432306	0.455223
WIPI1	0.00575	0.432306	0.509983
ODF2L	0.005743	0.432306	0.293251
TSPAN3	0.005688	0.431436	0.422097
LINGO3	0.005684	0.431436	0.277263
LOC40109:	0.005671	0.431436	0.467435
ZNF267	0.005542	0.428266	0.471573
RNU5E-9P	0.005531	0.428266	1.030952
CCRN4L	0.005485	0.427162	0.553101
MMGT1	0.005458	0.427162	0.235186
TRIM16	0.005455	0.427162	0.443216
4-Mar	0.005368	0.425748	0.597659
RPL22L1	0.005283	0.424421	0.305898
RND3	0.005141	0.423798	0.568997
CCND2	0.005063	0.420656	0.36763
RMI2	0.005053	0.420656	0.409682
SNX18	0.004995	0.417805	0.408456
LHX5	0.004922	0.416505	0.336444
SNORA12	0.004849	0.413073	1.055702
LOC10028:	0.004791	0.412018	0.918934
C19orf24	0.004789	0.412018	0.54894
H1F0	0.004769	0.412018	0.429139
APH1B	0.004703	0.409918	0.479091
CDKN2C	0.004631	0.405913	0.761597
LPPR2	0.004605	0.40578	0.417634
KIR2DL3	0.004594	0.40578	0.661254
NCAM1	0.004568	0.40578	0.514789
TSPAN17	0.004556	0.40578	0.302703
NCF1B	0.004525	0.40578	0.952682
CYBA	0.004355	0.398085	0.411442
C11orf9	0.004352	0.398085	0.526042
HCST	0.004313	0.39708	1.034031
IP6K1	0.004308	0.39708	0.800195
SCARNA10	0.004276	0.396457	1.278499
VAC14	0.004271	0.396457	0.347162
RNF138	0.004154	0.391389	0.60875
ATP1B4	0.004143	0.391389	0.33782
GPX1	0.004132	0.391389	0.354493
CPE	0.004112	0.391389	0.810939
UBE2Z	0.004103	0.391389	0.399724
CITED4	0.004067	0.391389	0.676214
MED28	0.004051	0.391389	0.35521

ZNF35	0.004009	0.390989	0.459495
MMP15	0.003995	0.390989	0.851481
LOC10050!	0.003985	0.390989	0.458577
DPH3	0.003946	0.390765	0.439878
BGN	0.00385	0.384918	0.793536
EFR3A	0.003844	0.384918	0.403439
ORC6	0.003839	0.384918	0.540025
PNPLA2	0.003788	0.383474	0.497773
MUC1	0.003759	0.382965	0.930378
PTPN23	0.003758	0.382965	0.459259
NOP10	0.003705	0.382965	0.392891
NUDT14	0.003616	0.382965	0.980204
CELSR1	0.003605	0.382965	0.458046
LAPTM5	0.003562	0.38109	0.563321
DESI1	0.003395	0.370669	0.320934
UCHL1	0.003315	0.365635	0.370409
ZNF121	0.003297	0.365635	0.604349
FMOD	0.003209	0.365367	0.883908
LOC10065:	0.003193	0.364776	0.601411
RNF224	0.003189	0.364776	0.518976
TIMP1	0.003167	0.364776	0.506322
SERPINA1	0.003087	0.360455	0.424587
TFPI	0.00307	0.360163	0.613342
PPP1R18	0.003031	0.337173	0.680106
FADS3	0.003018	0.357568	0.337132
PEX26	0.002998	0.357568	0.255483
NDE1	0.002961	0.354766	0.759709
LOC38882(	0.00296	0.354766	0.497327
TCIRG1	0.002927	0.353382	0.450133
CLDN12	0.002875	0.349934	0.480864
TEAD4	0.002864	0.349934	0.621196
RNF157-AS	0.002821	0.349934	0.609483
RAC2	0.002777	0.348391	0.810074
TMEM181	0.002748	0.346066	0.493916
OR2A20P	0.002743	0.346066	0.737142
VSTM2L	0.002589	0.336668	0.733478
VCAN	0.002585	0.336668	0.888958
TGFBR3L	0.002488	0.336668	0.550973
RAP2B	0.002452	0.333836	0.413597
ADTRP	0.00245	0.333836	0.253098
CD44	0.002447	0.333836	0.578135
DLL3	0.002411	0.33242	0.460681
CCNE1	0.002405	0.33242	0.426332
ANO5	0.002389	0.33242	0.679646
WBSCR16	0.002384	0.33242	0.888675
CAMKK2	0.00238	0.33242	0.305684
LDOC1L	0.002373	0.33242	0.500603

VENTX	0.002347	0.33242	0.465279
CBR3	0.002316	0.33242	0.620287
ABCA3	0.002297	0.33242	0.808617
YDJC	0.00227	0.33242	0.47954
ARPC1B	0.00227	0.33242	0.456057
CLDN7	0.002217	0.33242	0.816055
COMMD9	0.002157	0.32707	0.281916
LOC10050	0.002148	0.32707	1.067612
ENPP5	0.00209	0.324573	0.6076
CD48	0.002049	0.322869	0.908235
MMD	0.002036	0.322455	0.620109
VILL	0.002008	0.320906	0.667494
KIAA1586	0.001989	0.319688	0.511888
SNAP23	0.001986	0.319688	0.384054
DERL1	0.001983	0.319688	0.487122
IER3	0.001972	0.321786	0.810639
IFI30	0.00192	0.319688	0.6881
SLC29A1	0.001908	0.319688	0.791624
ANAPC1	0.001905	0.319688	0.655394
PXDC1	0.00185	0.319688	0.578348
MECR	0.001846	0.319688	0.569472
SLC39A13	0.00183	0.319688	0.466279
LPXN	0.001811	0.319688	0.912063
OLFML2B	0.001787	0.319688	0.862165
PLGRKT	0.001786	0.319688	0.634866
AP2S1	0.001758	0.319552	0.438103
FAM129B	0.001749	0.319552	0.379679
CCL21	0.001739	0.319552	2.138012
AES	0.001714	0.318708	0.332935
PEX5L-AS2	0.001713	0.318708	0.394473
ADCY8	0.001711	0.318708	0.45442
MYOZ2	0.001676	0.318708	1.382462
CCDC64B	0.001623	0.31325	0.356109
CCDC113	0.001586	0.31309	0.534192
EIF5A2	0.001535	0.308836	0.979635
FAM92B	0.001501	0.307365	0.466732
UBE2H	0.001457	0.306553	0.445217
HGS	0.001434	0.306227	0.579328
S100A11	0.001432	0.306227	0.450997
SEMA7A	0.001425	0.306227	1.152799
FN1	0.001377	0.306227	1.118595
KPNA2	0.001373	0.306227	0.972467
VDAC1	0.001362	0.306227	0.509023
R3HDM4	0.00135	0.306227	0.538339
PMS2P3	0.001322	0.306227	0.918328
FHL3	0.001318	0.306227	0.828093
CFD	0.001316	0.306227	0.612823

MYLK3	0.001248	0.306227	1.132387
SPNS1	0.001235	0.306227	0.348144
KLHL21	0.001226	0.306227	0.408946
FAM210B	0.001215	0.306227	0.616093
NAA60	0.001188	0.306227	0.471505
SLC36A1	0.001185	0.306227	0.349128
LOC10050	0.001157	0.303317	0.383226
SNORA78	0.001124	0.299719	0.878607
DNAPTP3	0.001112	0.299719	1.127977
TLR6	0.001102	0.299719	0.534747
DAP	0.001083	0.299719	0.341656
CTSD	0.001083	0.299719	0.310579
CLCF1	0.001073	0.299719	0.770441
TOR1A	0.001073	0.299719	0.402204
CTSS	0.001058	0.299719	0.890571
S100A10	0.001055	0.299719	0.465003
APOC1	0.001047	0.299719	0.799003
ALG3	0.00104	0.299719	0.417367
ANKRD37	0.001012	0.299719	0.704261
CCDC90A	0.000886	0.292106	0.373885
SLC2A6	0.000853	0.288362	0.482042
SNORA48	0.000833	0.288362	0.429486
CCDC166	0.000827	0.288362	0.667081
MSMO1	0.000822	0.288362	0.471047
TRIM47	0.000819	0.288362	0.670462
STIL	0.000809	0.288362	0.661358
GCNT4	0.000803	0.288362	1.037566
MFSD12	0.000783	0.288362	0.616275
NT5E	0.000761	0.288362	1.189211
HEY2	0.000746	0.288362	1.078532
TYROBP	0.000727	0.288362	1.065247
HOMER3	0.000699	0.288362	0.985449
CSF2RB	0.000663	0.288362	0.912023
LOC10029	0.000659	0.288362	0.506479
NDUFA7	0.000595	0.288362	0.434203
LYZ	0.000564	0.288362	0.77305
PPP1R3C	0.000562	0.288362	0.921327
DDR1	0.000548	0.286872	0.449519
LUM	0.000514	0.288362	1.52041
SHC1	0.000514	0.288362	0.627412
RPA4	0.00051	0.288362	0.389293
DHCR24	0.000447	0.288362	0.827663
KXD1	0.000437	0.288362	0.618998
SNORA22	0.000422	0.288362	0.803881
RN5S60	0.000411	0.288362	0.507911
FAM49B	0.000388	0.288362	0.472184
MAN2B1	0.000292	0.288362	0.402094

TNFRSF12A	0.000256	0.272535	2.850331
CRIP1	0.000237	0.272535	0.639871
ATP6V0D1	0.000175	0.272535	0.473646
UNC93B1	0.000147	0.272535	0.853342
FAM22F	0.000107	0.263902	0.875801
IL10RA	0.000101	0.263902	1.00376
SNORD116	8.55E-05	0.248632	1.498301
B4GALT6	8.53E-05	0.248632	0.847976
EMP1	6.06E-05	0.248632	1.082341
LRRC8A	3.67E-05	0.234972	0.492781
MTHFSD	9.67E-06	0.154619	0.847003
LOC10050(	0.049865	0.64662	-0.26223
LOC64797!	0.049855	0.64662	-0.12629
TRBV7-7	0.049854	0.64662	-0.16463
EFCAB7	0.04976	0.64656	-0.32925
PLK1S1	0.049663	0.646512	-0.39816
LOC28636	0.049662	0.646512	-0.78223
LOC10050	0.049607	0.646512	-0.31539
LOC20026	0.049606	0.646512	-0.26009
LOC25412	0.049583	0.646512	-0.43602
DMGDH	0.049579	0.646512	-0.2717
RAB11FIP4	0.04957	0.646512	-0.21515
LOC64320	0.049506	0.646512	-0.50949
RNASE7	0.049485	0.646512	-0.74482
SEL1L	0.049438	0.646512	-0.22906
CHRM3-AS	0.049365	0.646512	-0.17535
DEFA4	0.049335	0.646512	-0.76266
LOC10012!	0.049245	0.646512	-0.23385
OR5AS1	0.049243	0.646512	-0.25786
TSPAN16	0.04909	0.646512	-0.49143
GCC2	0.049006	0.646512	-0.34087
NSD1	0.049003	0.646512	-0.30452
HMCN2	0.048928	0.646482	-0.33184
LOC44091(	0.048927	0.646482	-0.38122
PCIF1	0.048888	0.646482	-0.33854
TAF2	0.048841	0.646172	-0.31424
CLCNKA	0.048808	0.646172	-0.37526
C17orf74	0.048792	0.646172	-0.27035
NPHS1	0.048746	0.646172	-0.34073
GCSH	0.048734	0.646172	-1.07621
HS3ST3A1	0.048704	0.646172	-0.2723
KIAA1704	0.048439	0.644306	-0.66268
CPA6	0.048418	0.644306	-0.21909
GOLGA2P5	0.048379	0.644306	-0.42672
EPN2-IT1	0.048336	0.644019	-0.18594
ATP5J	0.048284	0.644019	-0.29546
ARHGAP32	0.048273	0.644019	-0.75353

ACBD4	0.048269	0.644019	-0.56781
SLC22A5	0.048241	0.644019	-0.32674
OR1L3	0.048155	0.644019	-0.24893
MIR3156-2	0.048131	0.644019	-0.33777
LOC14869(	0.048042	0.644019	-0.45227
TMEM150/	0.047851	0.644019	-0.45496
NEBL	0.047694	0.643409	-0.27781
MIR520G	0.047666	0.643391	-0.30656
CYP4A11	0.047658	0.643391	-0.53825
GNAI1	0.047628	0.643391	-0.20682
HNRPDL	0.047399	0.642219	-0.23171
RN5S81	0.04736	0.641962	-0.08086
PCMTD2	0.047313	0.641719	-0.21921
MIR1976	0.047088	0.640783	-0.30006
SRSF4	0.047012	0.640783	-0.15141
DDX27	0.046979	0.640783	-0.31634
SAMD5	0.046941	0.640783	-0.43336
RABGAP1L	0.046915	0.640783	-0.26484
C1orf145	0.046828	0.640672	-0.04095
SPINK5	0.04677	0.640672	-0.37567
MIRLET7F1	0.046668	0.640454	-0.08093
ADNP	0.046607	0.640323	-0.1711
GSTA1	0.046599	0.640323	-0.6206
LOC10021(	0.046485	0.640134	-0.6473
SNORD32A	0.046439	0.640134	-0.21344
ODZ3	0.046417	0.640134	-0.44342
OR5D18	0.04638	0.640077	-0.20017
SLC35A1	0.046303	0.639339	-0.63554
KLHL24	0.046286	0.639339	-0.39272
UBTF	0.046275	0.639339	-0.19469
FABP1	0.046263	0.639339	-0.40958
DUX4L9	0.046251	0.639339	-0.67348
STON2	0.046209	0.639339	-0.58106
RASSF8	0.046038	0.639339	-0.28214
ILF3	0.045978	0.639339	-0.17623
TAS2R31	0.045951	0.639339	-0.21889
C15orf29	0.045947	0.639339	-0.17815
C20orf11	0.045751	0.638561	-0.22134
LOC44002{	0.045729	0.638561	-0.63659
KRBA2	0.045545	0.63775	-0.32911
MIS18A	0.045456	0.637502	-0.50229
ATG10-IT1	0.045322	0.636461	-0.31878
ZADH2	0.045235	0.635568	-0.51046
SPATA8	0.045161	0.635325	-0.12817
MBD3L1	0.045017	0.634962	-0.2828
ANKRD12	0.044693	0.63239	-0.15521
EPC1	0.044667	0.63239	-0.43741

LOC10013:	0.04464	0.63239	-0.35555
WNT3A	0.044615	0.63239	-0.42265
KIAA1009	0.044533	0.63239	-0.07172
SKOR1	0.044469	0.631972	-0.34943
MAN1A2	0.044428	0.631972	-0.28436
MGC12982	0.044397	0.631972	-0.77546
PCK2	0.044169	0.630609	-0.46275
KC6	0.044167	0.630609	-0.14492
KIFAP3	0.04416	0.630609	-0.27955
RNU7-51P	0.044067	0.630456	-0.56016
SLC6A16	0.043991	0.630317	-0.76616
RNU6-63	0.043848	0.630317	-0.31989
RHPN1	0.043846	0.630317	-0.29137
LOC44117!	0.04383	0.630317	-0.15872
UBE2V2	0.043792	0.630317	-0.15856
TMEM150	0.04378	0.630317	-0.45325
LOC64366!	0.043763	0.630317	-0.25405
OR10C1	0.043565	0.629759	-0.31441
OR2T6	0.043561	0.629759	-0.76386
KIF27	0.04344	0.629664	-0.38809
ZNF608	0.043434	0.629664	-0.54725
MIR1911	0.043422	0.629664	-0.38354
RN5S489	0.043318	0.629664	-0.41332
SUV420H1	0.04322	0.629664	-0.36597
SMARCA2	0.043014	0.629228	-0.18898
6-Mar	0.042952	0.628847	-0.1534
C10orf118	0.042925	0.62874	-0.27415
PHOX2A	0.042876	0.628594	-0.36338
LOC10028!	0.042839	0.628516	-0.3585
GSTT2	0.042834	0.628516	-0.66705
CWC27	0.042552	0.626431	-0.15077
YWHAE	0.042499	0.626431	-0.20004
S100A7	0.042463	0.626431	-0.19493
FABP2	0.0423	0.625812	-0.29672
FEV	0.042237	0.625385	-0.26293
RN5-8S4	0.042049	0.623451	-0.54081
ODC1	0.042049	0.623451	-0.23669
POM121L9	0.042011	0.623451	-0.31414
UPK3B	0.041977	0.623451	-0.18364
PIK3IP1	0.041899	0.623402	-0.35304
UACA	0.041863	0.623174	-0.55466
SMU1	0.041768	0.622046	-0.33204
ELAC1	0.041715	0.621932	-0.49246
GPR19	0.041578	0.621235	-0.2902
SNORA27	0.041395	0.620311	-0.32256
MDFIC	0.041213	0.618395	-0.26895
DNAJC27-/	0.041075	0.618395	-0.51499

HOXD8	0.041006	0.617884	-0.37582
NPIP	0.040949	0.617884	-0.30673
MIR153-1	0.04087	0.617884	-0.20972
LOC100050(	0.040828	0.617884	-1.03883
IGBP1	0.040821	0.617884	-0.3056
LOC72771(	0.040801	0.617884	-0.26295
SFXN2	0.0407	0.617884	-0.54949
ANO1-AS1	0.040672	0.617884	-1.0739
RARRES2	0.040662	0.617884	-0.16683
TNFRSF8	0.040647	0.617884	-0.19821
DEFB119	0.040601	0.617884	-0.33468
LOC40075:	0.040528	0.617884	-0.61816
ZNF847P	0.04042	0.617766	-0.26687
SETD4	0.040365	0.617238	-0.3064
PA2G4	0.040328	0.61715	-0.14958
COL21A1	0.040286	0.61706	-0.49521
H3F3B	0.040233	0.616994	-0.24812
ELAVL1	0.040075	0.616345	-0.21672
LINC00324	0.040034	0.616345	-0.50557
DEPTOR	0.040001	0.616345	-0.30555
CYP2C18	0.039878	0.616345	-0.18008
LOC10012!	0.039684	0.616196	-0.23374
MGAM	0.039514	0.616196	-1.137
TRAJ56	0.039384	0.615781	-0.1582
FLJ30838	0.039309	0.61551	-0.15965
LOC28646:	0.039269	0.615497	-0.46335
SLC4A1AP	0.039261	0.615497	-0.21586
AZGP1	0.039255	0.615497	-0.41379
PHOSPHO2	0.039148	0.615097	-0.37324
11-Mar	0.039025	0.615097	-0.16272
FLJ46020	0.03902	0.615097	-0.14633
LINC00290	0.038887	0.615097	-0.20933
LOC10012!	0.038784	0.614519	-0.25116
SIK3-IT1	0.038736	0.614058	-0.4147
KLHL8	0.038683	0.614058	-0.38094
DDC	0.038671	0.614058	-0.53726
LOC90834	0.038532	0.614058	-0.4917
ZNRF3	0.038503	0.614058	-0.42401
TPTE2P5	0.038462	0.614058	-0.56021
FAM135A	0.03843	0.614058	-0.34022
C8orf12	0.038421	0.614058	-0.40233
ABAT	0.038413	0.614058	-0.63959
C11orf84	0.038314	0.614058	-0.26624
LSM11	0.038209	0.614058	-0.80221
CYP21A1P	0.038096	0.613773	-0.70652
GJC2	0.038032	0.613773	-0.50425
TSPAN9	0.037971	0.613773	-0.34348

CDK11A	0.037894	0.613483	-0.31337
CCDC14	0.037851	0.613483	-0.24748
CCP110	0.037787	0.613483	-0.26768
BMS1P4	0.037771	0.613483	-0.91406
RN5S404	0.037706	0.613483	-0.11446
OR2T10	0.037623	0.613483	-0.95585
TMEM121	0.037589	0.613483	-0.45518
OR10H4	0.03756	0.613483	-0.65687
C5orf34	0.037541	0.613483	-0.19831
FGFR4	0.037523	0.613483	-0.51064
RTN4	0.037505	0.613483	-0.17156
LOC38815	0.037476	0.613483	-0.69042
LOC64535	0.037469	0.613483	-0.27289
C11orf54	0.037415	0.613483	-0.30384
SYDE2	0.037389	0.613483	-0.3061
BEND7	0.037331	0.613483	-0.40763
TBC1D19	0.037199	0.612448	-0.23809
RN5S381	0.037106	0.612183	-0.30268
MAGI2	0.036886	0.610379	-0.22297
EML4	0.036841	0.610379	-0.36498
AKR1B15	0.036806	0.610379	-0.38367
THNSL1	0.036708	0.61033	-0.36443
MAGEF1	0.036699	0.61033	-0.18333
UFL1-AS1	0.036632	0.610272	-0.30864
MORN3	0.036586	0.610272	-0.50784
WDR33	0.036535	0.610272	-0.23782
SLIT3	0.036534	0.610272	-0.24734
TULP4	0.036527	0.610272	-0.2063
CHD1	0.036396	0.609582	-0.14182
ZNF414	0.036274	0.609136	-0.21106
FBXO9	0.036249	0.609136	-0.30794
DEK	0.036174	0.609136	-0.15135
SLC5A11	0.03617	0.609136	-0.57939
OSBPL1A	0.036125	0.609136	-0.09734
CYP2F1	0.036058	0.609136	-0.39064
LOC28574	0.036045	0.609136	-0.40453
FERD3L	0.03601	0.609136	-0.39832
PTMS	0.035893	0.609063	-0.15445
CREG2	0.035823	0.608911	-0.31984
PTPN12	0.035822	0.608911	-0.2639
TEX11	0.035766	0.608838	-0.42592
LOC72960	0.035755	0.608838	-0.66831
SCAF8	0.03575	0.608838	-0.28718
KATNAL1	0.035698	0.608838	-0.21979
HDAC11-A	0.0356	0.608366	-0.28333
RNU7-87P	0.035445	0.608366	-0.40944
MIR4320	0.035348	0.607953	-0.34747

LRPPRC	0.035325	0.607953	-0.21053
MIR802	0.035304	0.607953	-0.41224
CXCR7	0.035247	0.60784	-0.81033
MIR1538	0.035215	0.607617	-0.92735
TMEM86B	0.035166	0.607108	-0.46517
NAALADL2	0.035151	0.607108	-0.20678
LOC10050	0.03511	0.607108	-0.47778
OR8B4	0.035089	0.607092	-0.37124
LOC10050	0.035061	0.607092	-0.20134
ACTR8	0.034994	0.606429	-0.05299
TGM5	0.034965	0.606405	-0.09946
CCL7	0.03496	0.606405	-0.40233
PHACTR2	0.034948	0.606405	-0.16687
RAB41	0.034939	0.606405	-0.47392
BEX4	0.034931	0.606405	-0.24886
LOC44089	0.0349	0.606405	-0.62098
CNNM2	0.034895	0.606405	-0.33443
TRPC7	0.034853	0.606405	-0.51144
IL1A	0.034843	0.606405	-0.24224
ANGPTL3	0.034829	0.606405	-0.51736
MBD2	0.034783	0.606405	-0.19913
DCAF16	0.034773	0.606405	-0.34088
LOC10050	0.034749	0.606405	-0.51164
RNU7-81P	0.03472	0.606405	-0.4843
ZNF789	0.034685	0.606405	-0.23109
LOC10013	0.034651	0.606405	-0.10405
AGPAT9	0.034614	0.606405	-0.48443
THAP8	0.034607	0.606405	-0.31087
OR10D3	0.03452	0.606405	-0.4014
MIR4662A	0.034229	0.603955	-0.39124
MIR4759	0.034099	0.602342	-0.65917
DYNC2H1	0.034051	0.601826	-0.48193
SLC15A5	0.034021	0.601612	-0.27152
LOC10050	0.033979	0.601552	-0.58318
CHD2	0.033941	0.601532	-0.23521
SLC7A7	0.033825	0.600666	-0.42711
HECTD1	0.033788	0.600666	-0.18927
AIF1	0.03375	0.601152	-0.36597
RBP2	0.033686	0.600666	-0.26123
BMPER	0.033475	0.600263	-0.50941
LINC00475	0.033473	0.600263	-0.41717
LOC14814	0.033308	0.59895	-0.25763
PROX1-AS	0.032957	0.595898	-0.3293
LOC14937	0.032902	0.595898	-0.31774
LOC10013	0.032888	0.595898	-0.33255
GABARAPL	0.032886	0.595898	-0.498
CGNL1	0.032878	0.595898	-0.10904

GALNTL4	0.032877	0.595898	-0.47473
FBXO3	0.032847	0.595898	-0.31048
ZNF98	0.0328	0.595898	-0.0739
DOCK1	0.032783	0.595898	-0.21749
LOC10050!	0.032745	0.595898	-0.76612
DRD3	0.032713	0.595898	-0.01891
RABL2A	0.032659	0.595898	-0.46031
ULK4	0.032576	0.595898	-0.40505
CIDEB	0.032575	0.595898	-0.23484
CNTNAP3	0.032563	0.595898	-0.5733
PTPRD	0.032441	0.595898	-0.28222
LAMC1	0.032369	0.595898	-0.26862
CDC73	0.032148	0.593425	-0.2158
RN5S178	0.032084	0.592754	-0.35264
NDN	0.032037	0.592754	-0.36105
CNBP	0.032021	0.592754	-0.06549
GPR25	0.032001	0.592754	-0.47089
FAM226B	0.031996	0.591858	-0.292
ILVBL	0.031988	0.592754	-0.25617
SETD2	0.031974	0.592754	-0.2609
FLJ45743	0.031961	0.592754	-0.34875
OGT	0.031913	0.592754	-0.3132
RN5S445	0.031845	0.592754	-0.16084
LRRC6	0.031685	0.592051	-0.2281
COL28A1	0.031656	0.591857	-0.2037
CEP112	0.03164	0.591857	-0.2199
CCR9	0.031582	0.59165	-0.36131
ACADM	0.031479	0.59126	-0.48022
INTS7	0.031415	0.59081	-0.45519
FAM75A6	0.031257	0.589264	-0.19683
WBP2NL	0.03122	0.589218	-0.31282
LOC10085!	0.031159	0.588761	-0.25069
MESTIT1	0.03107	0.587818	-0.36784
MIR521-1	0.031028	0.587818	-0.43512
DNAJB4	0.030936	0.587818	-0.17735
KIAA0825	0.030922	0.587818	-0.1951
SNORA74A	0.030843	0.587818	-0.32392
CCDC117	0.030794	0.587818	-0.2238
RNF214	0.030762	0.587818	-0.25606
LOC64521:	0.030757	0.587818	-0.28652
SPHK2	0.030626	0.587818	-0.25613
TEX28	0.030431	0.58751	-0.19154
ITLN1	0.030383	0.587327	-0.30425
ZSCAN1	0.030381	0.587327	-0.21978
ENPEP	0.030278	0.587327	-0.29863
RALGAPA1	0.030182	0.587327	-0.28253
FRA10AC1	0.030146	0.587327	-0.3442

RSF1	0.030075	0.587327	-0.11694
PRSS3	0.029991	0.587327	-0.36506
MEF2C	0.029925	0.587327	-0.3657
RN5S443	0.029919	0.587327	-0.4335
DOK6	0.029831	0.586898	-0.86841
MBTD1	0.029791	0.586639	-0.23577
TCF7L1-IT1	0.029778	0.586639	-0.63875
MIR103B1	0.029709	0.586639	-0.69501
OR4K14	0.029701	0.586639	-0.03033
TET1	0.029688	0.586639	-0.48543
EHBP1	0.029687	0.586639	-0.4824
ZNF540	0.029648	0.586639	-0.27359
C11orf49	0.029595	0.586639	-0.25547
GZF1	0.029595	0.586639	-0.29185
RCBTB2	0.029521	0.586639	-0.29727
KRT76	0.029471	0.586639	-0.32816
SNORA55	0.029456	0.586639	-0.32336
BPIFB4	0.029285	0.585156	-0.25756
RN5S238	0.029197	0.584848	-0.20349
SNORA54	0.029195	0.584848	-0.45475
SNORD12	0.029135	0.584338	-0.44089
WWC2-AS:	0.029101	0.584338	-0.3937
TIAL1	0.028994	0.583344	-0.42241
MCRS1	0.02898	0.583344	-0.63798
FAM20B	0.028965	0.583344	-0.27496
JMJD1C	0.028961	0.583344	-0.3075
OTOP1	0.028927	0.583344	-0.38893
HEY1	0.0289	0.583344	-0.36463
OTOA	0.028894	0.583344	-0.34224
IRF9	0.028872	0.583344	-0.5457
LOC72811	0.028853	0.583344	-0.28452
CHCHD6	0.028782	0.583344	-0.23311
MIR3678	0.028767	0.583344	-0.87592
FAM126B	0.028749	0.583344	-0.32755
DLX6-AS1	0.028722	0.583344	-0.28429
HPD	0.028709	0.583344	-0.94367
LOC10050	0.028654	0.583344	-0.32976
ZFP62	0.028649	0.583344	-0.41304
MIR548AA	0.028645	0.583344	-0.28489
RNU7-84P	0.02864	0.583344	-0.27902
LINC00570	0.02852	0.583344	-0.05781
CHL1-AS2	0.02844	0.583344	-0.7861
MIR1294	0.028389	0.583344	-0.52072
RN5S444	0.028339	0.583344	-0.92041
RNU6-71	0.028295	0.583344	-0.1598
SHFM1	0.028284	0.578518	-0.427
PSIP1	0.028219	0.583344	-0.26834

RPS6KA6	0.028203	0.583344	-0.41754
MIR1185-2	0.028194	0.583344	-0.27538
NOL10	0.028152	0.583344	-0.30072
LOC100630	0.02804	0.583344	-0.55423
PM20D2	0.028037	0.583344	-0.4431
RPGR	0.028033	0.583344	-0.21851
RBM28	0.028004	0.583344	-0.49591
SLC26A10	0.027967	0.583344	-0.6299
IMPACT	0.027844	0.583344	-0.36934
RIBC1	0.027741	0.583344	-0.15811
HNRNPD	0.027731	0.583344	-0.22475
OR10J5	0.027622	0.582813	-0.13261
LOC10013	0.027552	0.582813	-0.89693
C6orf203	0.027536	0.582813	-0.41888
SHANK2-AS	0.027468	0.582813	-0.36581
UNC50	0.027442	0.582813	-0.2273
FAM65B	0.027386	0.582813	-0.49781
C3orf74	0.027362	0.582813	-0.27497
CUL3	0.027306	0.582813	-0.28221
CYP4F3	0.027041	0.582756	-0.96199
FAM156B	0.026913	0.581722	-0.27423
PRAMEF15	0.026715	0.580455	-0.35545
HRASLS5	0.026593	0.579113	-0.28043
MIR2681	0.026515	0.577805	-0.38694
HOXA-AS2	0.026258	0.575345	-0.67708
LOC10065	0.026171	0.575258	-0.45285
ZBTB48	0.026165	0.575258	-0.49003
DCC	0.026137	0.575258	-0.19322
OPN5	0.026131	0.575258	-0.37905
MLLT10	0.026105	0.575258	-0.39952
FBXO18	0.026018	0.574794	-0.34915
TMPRSS5	0.026007	0.574794	-0.16404
ASS1	0.025933	0.57371	-0.44034
BMP4	0.025897	0.57371	-0.44262
ZNF711	0.025853	0.573698	-0.65524
TRAJ1	0.025818	0.573698	-0.32571
CDY2A	0.025798	0.573698	-0.55629
LOC38976	0.025665	0.572541	-0.63245
MYO5C	0.025638	0.57233	-0.34294
ACVR2A	0.025617	0.572263	-0.26893
ROCK2	0.025524	0.57137	-0.27972
MIR4452	0.025523	0.57137	-0.22421
CLPX	0.025507	0.57137	-0.26279
LOC10013	0.025418	0.57137	-0.52063
RRAGA	0.025398	0.57137	-0.23075
ADHFE1	0.025361	0.57137	-0.47514
FLJ34521	0.025327	0.57137	-0.30065

DGCR9	0.025192	0.5694	-0.5606
NAALADL2	0.025135	0.569123	-0.158
RN5S20	0.02513	0.569123	-1.46086
SMARCC1	0.02513	0.569123	-0.32321
KRTAP10-4	0.025123	0.569123	-0.39072
MAST2	0.025013	0.569042	-0.51564
MIR4729	0.024997	0.569042	-0.62528
ATP1B2	0.024963	0.569042	-0.51266
PHAX	0.024916	0.568633	-0.27425
PVRL1	0.024872	0.568633	-0.36035
TPTE2P1	0.02474	0.56612	-0.79401
FLJ13197	0.024717	0.56612	-0.43271
ARL3	0.02468	0.565964	-0.26562
SHF	0.024672	0.565964	-0.25885
OR1M1	0.02466	0.565964	-0.69139
CENPT	0.024609	0.565964	-0.26314
RN5S187	0.024564	0.565964	-0.89235
RN5S137	0.024503	0.565964	-0.64155
PRPSAP2	0.024383	0.565964	-0.10166
TAPBPL	0.02436	0.565964	-0.31053
ERAS	0.024031	0.564214	-0.28481
ZC3H4	0.023976	0.563541	-0.42941
SMC3	0.023912	0.562862	-0.15099
FGF9	0.023901	0.562862	-0.37259
SKIV2L2	0.023757	0.561106	-0.15663
LOC10050	0.023687	0.561106	-0.2312
RBM12	0.023664	0.561106	-0.31265
LOC10050	0.023662	0.561106	-0.42015
UBOX5-AS	0.023659	0.561106	-0.37782
OCLM	0.023652	0.561106	-0.28203
NIPBL	0.023618	0.561106	-0.20917
LINC00347	0.023616	0.561106	-0.369
DKFZp451	0.023605	0.561106	-0.27334
HAO2-IT1	0.023497	0.561106	-0.84589
LOC33966	0.023444	0.561106	-0.34048
FAT2	0.023351	0.561106	-0.31284
SPOCK3	0.023321	0.561106	-0.01201
ZFYVE19	0.023274	0.561106	-0.54042
LOC10012	0.023233	0.561106	-0.69978
LINC00571	0.023221	0.561106	-0.42019
ALDH6A1	0.023149	0.561106	-0.54763
RAD54L2	0.023098	0.561106	-0.33888
LINC00486	0.022815	0.560551	-0.39765
CCDC74B-/	0.022814	0.560551	-0.45985
RASEF	0.02275	0.56007	-0.51892
C19orf29-/	0.022641	0.558857	-0.44558
HIATL1	0.022544	0.558578	-0.24076

SUN1	0.022527	0.558578	-0.34289
CNIH3	0.022502	0.558578	-0.311
LOC10013:	0.022418	0.558578	-0.58907
MIR642B	0.022398	0.558578	-0.48595
LYZL1	0.022291	0.558578	-0.4871
BBS10	0.022267	0.558578	-0.45225
VRK1	0.022234	0.558578	-0.27744
WDR85	0.022175	0.558578	-0.36408
FLJ31183	0.022164	0.558578	-0.42086
NTRK2	0.022131	0.558578	-0.37027
BPTF	0.022112	0.558578	-0.23616
ZNF407	0.022074	0.558578	-0.34402
DDX11L1	0.022048	0.558578	-0.51835
7-Sep	0.022022	0.558578	-0.43538
PPP1R12B	0.021973	0.558578	-0.24392
PPIP5K1	0.021972	0.555485	-0.84326
ABCG5	0.021959	0.558578	-0.17533
TJP1	0.021916	0.558578	-0.2412
SART1	0.021627	0.558578	-0.27216
LOC10050!	0.021527	0.558578	-0.5068
DCLRE1A	0.021395	0.557352	-0.36483
API5	0.021395	0.557352	-0.27476
HMGA1P7	0.021372	0.557352	-0.70985
LOX	0.021264	0.557101	-0.56075
DSERG1	0.021218	0.556825	-0.6665
BHMT	0.021173	0.556089	-0.47858
ZCCHC11	0.021144	0.555783	-0.20121
TRAJ33	0.021038	0.554834	-1.40645
ANP32B	0.020919	0.554444	-0.22606
SLC35G6	0.020914	0.554444	-0.44233
MIR4460	0.020882	0.554444	-0.05235
DICER1-AS	0.020878	0.554444	-0.41662
THBS4	0.020811	0.554444	-0.34981
USP27X	0.020801	0.554444	-0.53477
CHD5	0.020777	0.554444	-0.46082
FBP2	0.020755	0.554444	-0.3499
RBM39	0.020729	0.554444	-0.28263
TTTY14	0.020692	0.554444	-0.72693
HAT1	0.020672	0.554444	-0.22058
TTLL9	0.020669	0.554444	-0.23339
PCBD2	0.020651	0.554444	-1.45369
DCTN3	0.020595	0.554444	-0.35842
GRM7-AS1	0.020581	0.554444	-0.31913
CXorf48	0.020492	0.554444	-0.27048
DUOX1	0.02022	0.552847	-0.22294
NKAPL	0.020055	0.549755	-0.41003
FGF22	0.02005	0.549755	-0.43298

GJA8	0.019961	0.548572	-0.62596
TPR	0.019944	0.548572	-0.30524
C16orf71	0.01989	0.548572	-0.23763
SLC1A6	0.019877	0.548572	-0.60233
NFX1	0.019847	0.548572	-0.39474
PRAMEF5	0.019786	0.548476	-0.26658
ELP3	0.019685	0.546163	-0.28271
LOC10050!	0.019587	0.545805	-0.1867
MSANTD4	0.019496	0.54421	-0.23351
BTF3	0.01944	0.543595	-0.24034
TIGD7	0.019385	0.543476	-0.6648
COL6A5	0.019349	0.543476	-0.22215
RNU7-76P	0.019311	0.54333	-0.57489
TET2	0.019234	0.542105	-0.42558
SNORD69	0.019198	0.542029	-1.05867
ZGPAT	0.01915	0.541941	-1.02394
ARHGAP35	0.019096	0.541284	-0.41145
FAM19A1	0.019079	0.541284	-0.26626
FLJ44006	0.01898	0.5402	-0.59596
WWC2	0.018903	0.539927	-0.34583
EFEMP1	0.018866	0.539335	-0.36644
NBEAL1	0.018862	0.539335	-0.36062
C14orf118	0.018677	0.535915	-0.31654
SVIP	0.018671	0.535915	-0.18235
PCK1	0.018589	0.535915	-0.68134
SRRM2-AS	0.018539	0.535915	-0.46475
NCKAP5	0.018536	0.535915	-0.32046
CCDC74A	0.01837	0.534946	-0.71954
POU4F1-A!	0.018322	0.534946	-0.44567
OR52A5	0.018215	0.534582	-0.56621
ARID4A	0.018202	0.534582	-0.50996
LOC10050!	0.018093	0.533456	-0.77749
ANKRD20A	0.018081	0.533456	-0.44278
DCAF12L1	0.018031	0.533456	-0.48865
C2orf53	0.018018	0.533456	-0.18802
LOC10012!	0.017835	0.532212	-0.58754
OR2T2	0.017827	0.532212	-0.57227
DHFRL1	0.017771	0.531786	-0.39418
POLE	0.01775	0.531678	-0.42015
ATP6V1G2	0.017721	0.531678	-0.36786
FBXO4	0.017678	0.531678	-0.37466
C20orf79	0.017642	0.531678	-0.24039
PAIP2	0.017558	0.53069	-0.21352
ZNF551	0.01749	0.53069	-0.60735
SEMA6D	0.017444	0.53069	-0.28661
LOC15112	0.017394	0.53069	-0.53885
SERPIND1	0.017289	0.53069	-1.03988

RNU1-17P	0.017231	0.53069	-0.29538
MIR3911	0.01711	0.53069	-0.61253
RNU7-80P	0.017065	0.53069	-0.98658
CLN8	0.01705	0.53069	-0.39006
CC2D2A	0.016916	0.53069	-0.33609
USP50	0.016909	0.53069	-0.26416
KANK1	0.01685	0.53069	-0.23842
TBC1D1	0.016821	0.53069	-0.32025
RN5S105	0.016779	0.53069	-0.40676
RN5S415	0.016679	0.53069	-0.59247
KRT15	0.016655	0.53069	-0.12709
RN5S469	0.016631	0.53069	-0.80247
CHMP2A	0.016623	0.53069	-0.17855
LOC100500	0.016466	0.53069	-0.27722
SYF2	0.016456	0.53069	-0.20942
STX16-NPE	0.016434	0.53069	-0.38023
ZNF518B	0.016431	0.53069	-0.40765
CLK2P	0.016385	0.53069	-0.76054
PPP2R2B-I	0.016374	0.53069	-0.37243
LOC100130	0.016288	0.530608	-0.18974
REPS2	0.01623	0.53008	-0.45726
MIR4715	0.016169	0.529949	-0.8126
C20orf196	0.016081	0.528606	-0.55428
EDNRA	0.016051	0.528606	-0.88047
LOC727993	0.016013	0.528606	-0.51781
LOC100500	0.016003	0.528606	-0.92908
RN5S448	0.015974	0.528606	-0.9295
CPLX1	0.015946	0.528606	-0.69762
LOC100500	0.015832	0.52801	-0.40545
IRGM	0.015828	0.52801	-0.51503
NCKAP5-IT	0.015768	0.527626	-0.34909
CCDC150	0.015672	0.527626	-0.24807
FLJ44385	0.015639	0.527626	-0.3873
TMEM95	0.015612	0.527626	-0.61877
PARD3B	0.015608	0.527626	-0.5143
RN5S46	0.015575	0.527626	-1.97168
LOC100500	0.015488	0.527626	-0.51975
SNORD84	0.015411	0.527626	-0.3332
A1CF	0.015245	0.525858	-0.41253
MRO	0.015195	0.52576	-1.13427
FAM183B	0.015188	0.52576	-0.40797
FAM170B	0.015071	0.524105	-0.29801
VCX	0.015057	0.524105	-0.2544
TRAC	0.014947	0.523719	-0.68306
SENP7	0.014934	0.523719	-0.30038
KBTBD10	0.014875	0.522384	-0.3346
PLCG2	0.014847	0.522384	-0.77426

LOC40158!	0.014813	0.521889	-0.50236
EMC1	0.014779	0.521889	-0.4218
ZAK	0.014768	0.521889	-0.39795
MIR3942	0.014526	0.517453	-0.44837
USP11	0.014496	0.517282	-0.14366
ZC2HC1C	0.01446	0.517282	-0.35253
SART3	0.014441	0.517282	-0.35035
LOC10050(	0.014369	0.516534	-0.32701
SMAD9-AS	0.014357	0.516534	-0.36798
SLTM	0.014322	0.516534	-0.24743
CA2	0.014282	0.516534	-0.52905
RNF216	0.014254	0.516393	-0.37306
C20orf90	0.014239	0.516393	-0.4809
SUPT16H	0.01422	0.516329	-0.19582
NAGS	0.014158	0.515832	-0.44412
LOC44099:	0.014151	0.443702	-0.5229
ITGAD	0.014135	0.515832	-0.48353
PRKD1	0.014129	0.515832	-0.28363
ASF1A	0.014083	0.515455	-0.36787
OR2S2	0.014067	0.515455	-0.15753
LOC72967!	0.014055	0.515455	-0.58962
MIR1226	0.014009	0.515455	-0.64768
NLGN1-AS:	0.013957	0.515455	-0.32862
PAX2	0.013779	0.514441	-0.62799
MIR1185-1	0.013598	0.514075	-0.29862
MIR4429	0.013589	0.514075	-0.37435
VIP	0.013583	0.514075	-0.47464
TRBV7-3	0.013565	0.514075	-0.51947
MC4R	0.01352	0.514075	-0.28492
STK33	0.0135	0.514075	-0.42473
SPANXN4	0.013493	0.514075	-0.41386
C3orf37	0.013468	0.514075	-0.33497
REM2	0.013367	0.51216	-0.41374
PRKG1	0.013241	0.509741	-0.59033
HNRNPUL1	0.013228	0.509741	-0.26498
ZNF638	0.013211	0.509741	-0.19083
ALCAM	0.013168	0.509741	-0.47673
SERPINI2	0.013153	0.509741	-0.50673
HMP19	0.013102	0.509741	-0.30254
LOC10050(	0.013085	0.509741	-0.51821
MFI2	0.013078	0.509741	-0.61823
PSEN2	0.013016	0.509741	-0.36364
LOC10012!	0.01296	0.509741	-0.47661
FAM25A	0.012946	0.509741	-0.3718
LOC10028!	0.012843	0.505197	-0.3418
ZFP161	0.012835	0.509552	-0.28913
LINGO2	0.012792	0.508983	-0.55607

C1QTNF4	0.012728	0.508509	-0.57369
PNN	0.012691	0.508509	-0.13047
LOC28493:	0.012679	0.508509	-0.3738
ALPL	0.012665	0.508509	-0.73768
TRNAI2	0.012641	0.508509	-0.49884
C3orf62	0.012617	0.508509	-0.39087
SLC17A6	0.012597	0.508509	-0.41628
NAP1L1	0.01249	0.507713	-0.26222
RN5S475	0.012485	0.507713	-0.48928
VN1R4	0.012482	0.507713	-0.3857
VPS4B	0.01247	0.507713	-0.22333
LOC39228	0.012465	0.507713	-0.30596
ANKUB1	0.012213	0.504779	-0.22584
KRCC1	0.012126	0.504649	-0.42
KRTAP9-3	0.01202	0.504649	-0.47903
MIR4718	0.012016	0.504649	-0.74389
TRAJ22	0.011958	0.504649	-1.78301
FCER2	0.011926	0.504649	-0.33663
DDX42	0.011917	0.504649	-0.35286
ANO3	0.011899	0.504649	-0.90873
RN5S250	0.011897	0.504649	-0.46045
TTC18	0.011896	0.504649	-0.2515
METAP1	0.011839	0.504649	-0.21677
EPHA7	0.011775	0.504649	-0.60796
OR51D1	0.011759	0.504649	-0.48854
RN5S123	0.011705	0.504649	-0.9814
GLOD5	0.011663	0.504649	-0.53292
ST20	0.011578	0.504649	-0.41923
C5orf4	0.011524	0.503643	-0.31485
ZNF232	0.011509	0.503643	-0.47853
TYW3	0.011483	0.503214	-0.33243
MATN3	0.011453	0.502568	-0.74431
G0S2	0.011369	0.500263	-0.68262
ZMYM2-IT	0.011352	0.500203	-1.07794
MIR378I	0.01124	0.498715	-0.7054
TMF1	0.011138	0.497999	-0.20153
ST3GAL6-A	0.01103	0.496863	-0.97591
NOL8	0.010971	0.496863	-0.23089
ASAH2B	0.010811	0.492654	-0.73097
SLC24A3	0.010659	0.488508	-1.29627
LOC10050	0.010551	0.486362	-0.68163
EIF4B	0.01055	0.486362	-0.29347
LOC44195	0.01055	0.486362	-0.64056
BHLHB9	0.010394	0.484688	-0.57227
LOC33978	0.010332	0.484688	-0.49264
WDR49	0.010316	0.484688	-0.61081
ZNRF3-AS1	0.010274	0.484688	-0.27942

COL6A6	0.010181	0.48444	-0.47108
MIR4305	0.010108	0.484418	-0.24164
LOC10050(	0.010044	0.484418	-0.42054
NDUFA8	0.009967	0.482383	-0.19936
FLJ39639	0.009962	0.482383	-0.76197
ZNF630-AS	0.009907	0.482383	-0.47532
LINC00424	0.009878	0.482383	-0.54071
HBP1	0.009796	0.482383	-0.24953
PAN2	0.00966	0.481806	-0.22425
ZC3H12B	0.0096	0.480612	-0.80738
RPL37	0.009511	0.478634	-0.6566
H2AFJ	0.009482	0.478634	-0.28813
CYP2A7	0.009474	0.478634	-0.56297
MIR4532	0.00946	0.478634	-1.84522
DUSP4	0.009456	0.478634	-0.74753
LOC10012!	0.009432	0.478634	-0.50439
ST8SIA4	0.009394	0.478634	-0.49155
ZP4	0.009249	0.476879	-0.29655
DISP2	0.009218	0.476879	-0.48506
FAAH	0.009082	0.476879	-0.21581
CCDC148	0.00908	0.476879	-0.51578
BRD1	0.009078	0.476879	-0.46137
TRAJ11	0.009059	0.476879	-0.51048
DAB1	0.009045	0.476879	-0.5478
OR4C15	0.009023	0.476879	-0.29716
CHPT1	0.009007	0.476879	-0.2952
BEST1	0.008985	0.476879	-0.34943
CAPN11	0.008953	0.476879	-0.39011
CYP3A7-CY	0.008932	0.476879	-0.77211
ADAMTS9-	0.008895	0.476879	-0.60992
NSFL1C	0.008886	0.476879	-0.26836
ZNF195	0.008804	0.476879	-0.2059
CRNKL1	0.008785	0.476879	-0.22579
CHST6	0.008674	0.475977	-0.581
LGR4	0.008595	0.47408	-0.25214
KIAA0754	0.008544	0.472877	-0.30491
FAM166B	0.008454	0.471959	-0.44975
OPN1MW2	0.008409	0.470806	-0.24923
PLEKHA5	0.008378	0.470806	-0.34298
AASS	0.008252	0.469978	-0.51611
MIR766	0.008213	0.469595	-0.46703
MMAB	0.008157	0.468913	-0.36724
KAT6B	0.008044	0.466839	-0.37577
WWP1	0.008018	0.466775	-0.48462
CRYGA	0.007995	0.466723	-0.61398
TRA2B	0.007991	0.466723	-0.33075
LINC00525	0.007982	0.466723	-0.15259

MIR3202-1	0.00798	0.466723	-0.35158
SLC25A48	0.007872	0.464252	-0.32759
TSPAN8	0.00779	0.463203	-0.69189
ARHGAP21	0.007734	0.462827	-0.20962
MIRLET7C	0.007691	0.462827	-0.84098
ARHGAP31	0.007611	0.460963	-0.47737
LOC10050(	0.0076	0.460963	-0.49075
MFAP1	0.007597	0.460963	-0.21919
ARMS2	0.007434	0.459106	-0.43279
SUMO1P3	0.007341	0.456008	-0.87177
C14orf178	0.007341	0.456008	-0.41332
DMAP1	0.007192	0.450585	-0.30106
ZNF521	0.007011	0.448399	-0.55795
HNRNPU	0.006979	0.448399	-0.32208
PLGLB1	0.006975	0.448399	-0.80525
TUB	0.006961	0.448399	-0.7497
HTATSF1	0.006956	0.448399	-0.39466
MIR4636	0.006835	0.44805	-0.61378
OR56A3	0.006798	0.448026	-0.49369
DPF2	0.006716	0.445758	-0.33791
ZNF415	0.006628	0.444499	-0.68019
MIR3662	0.006599	0.444335	-0.30868
RYR3	0.006474	0.439705	-0.83242
TFAMP1	0.006472	0.439705	-0.49385
BATF	0.006449	0.439705	-0.32671
NR3C2	0.006366	0.437655	-0.32169
LATS1	0.006349	0.437655	-0.30718
PRPF6	0.006321	0.436719	-0.30995
ZIC2	0.006235	0.434631	-0.47127
SLC16A10	0.006182	0.434631	-0.28586
RN5S215	0.006166	0.434631	-0.92545
RN5S322	0.006162	0.434631	-0.66221
CXCL5	0.006153	0.434631	-0.39334
PCMTD1	0.006149	0.434631	-0.33337
OR2AP1	0.006089	0.434631	-0.32387
GAB4	0.006046	0.434631	-0.4108
DNAH6	0.006032	0.434631	-0.39016
RN5S430	0.00597	0.434631	-0.29049
IQUB	0.005924	0.433661	-0.84801
LOH12CR2	0.00591	0.433661	-0.59749
PLEKHH2	0.005795	0.43317	-0.57749
NEO1	0.00579	0.43317	-0.36161
ACAA1	0.0056	0.428571	-0.62762
ZNF445	0.005595	0.394468	-0.5819
PBX1	0.005588	0.428571	-0.91386
GPR157	0.005501	0.427162	-0.49906
IDO2	0.005493	0.427162	-0.49635

ZSCAN21	0.005458	0.427162	-0.44486
DPP6	0.005442	0.427162	-0.64438
LOC38772:	0.005377	0.425748	-0.82163
LOC72814!	0.005341	0.425005	-0.60616
PPIEL	0.00532	0.424421	-0.56479
LCN8	0.005319	0.424421	-0.66643
TRMT10B	0.005314	0.424421	-0.60989
TACO1	0.005302	0.424421	-0.51045
OR4A15	0.005271	0.424421	-0.30976
SEBOX	0.005268	0.424421	-0.5174
OGFOD2	0.005252	0.424421	-0.34665
GRIN3A	0.005232	0.424421	-0.44885
PPFIBP2	0.005229	0.424421	-0.43118
DGCR5	0.005153	0.423798	-0.53825
SRFBP1	0.005126	0.423683	-0.47023
LRP6	0.005081	0.421061	-0.27777
MSH2	0.004993	0.417805	-0.5379
NAALADL2	0.004868	0.413073	-0.57463
CG030	0.004864	0.413073	-0.9041
FKBP9L	0.004752	0.411973	-0.40539
CPNE8	0.004748	0.411973	-0.24373
MIB1	0.004581	0.40578	-0.49971
LOC43994!	0.004543	0.40578	-0.46751
C10orf113	0.004533	0.40578	-0.50473
ANKS1B	0.004381	0.39926	-0.43991
TOP2B	0.004257	0.396457	-0.17979
OSTN-AS1	0.00416	0.391389	-0.73499
ADAMTS9	0.004159	0.391389	-0.38124
MTTP	0.004151	0.391389	-0.5743
FAM133B	0.0041	0.391389	-0.51037
RTP1	0.004009	0.390989	-0.29711
LOC10012!	0.003958	0.390765	-0.73912
ZFP42	0.003925	0.389966	-1.12158
YLPM1	0.003915	0.389966	-0.28191
IK	0.003838	0.384918	-0.32757
ZNF248	0.003761	0.370998	-0.62986
A2M-AS1	0.003753	0.382965	-1.10399
TXNIP	0.003747	0.382965	-0.4693
PDIA5	0.003684	0.382965	-0.4269
LOC72998:	0.003656	0.382965	-0.42366
HNRNPM	0.003655	0.382965	-0.36993
FZD2	0.003632	0.382965	-0.81429
GULP1	0.003513	0.377071	-0.28231
JMY	0.003509	0.377071	-0.34331
PPM1A	0.003421	0.372238	-0.32085
C3orf15	0.003356	0.367695	-0.5076
LOC73018·	0.003348	0.367695	-0.57892

C20orf160	0.003306	0.365635	-0.28865
TRIM9	0.003299	0.365635	-0.48232
RUNX1T1	0.003291	0.365635	-0.70126
RN5S99	0.003252	0.365635	-1.09104
RNY1P6	0.003247	0.365635	-0.32165
SMAD5	0.00318	0.364776	-0.39698
LIAS	0.003163	0.364776	-0.54656
PCM1	0.003048	0.359789	-0.25197
DSCAML1	0.002906	0.352157	-0.33839
GHR	0.002877	0.349934	-0.81416
BMS1P2	0.002869	0.349934	-0.24901
TRAJ42	0.002866	0.349934	-0.76841
POLR1E	0.002823	0.349934	-0.59286
SAFB	0.002798	0.349613	-0.3685
LOC44145!	0.002732	0.346066	-0.31501
FAM228A	0.002722	0.346066	-0.83741
HRG	0.00269	0.34558	-1.62935
MUC12	0.002689	0.34558	-0.26252
ZNF441	0.002674	0.34558	-0.52081
C12orf42	0.002587	0.336668	-0.31489
RN5S358	0.00255	0.336668	-0.84373
YSK4	0.002496	0.336668	-0.46781
CWC25	0.002398	0.33242	-0.47028
NEK1	0.002384	0.33242	-0.39723
CCDC111	0.002284	0.33242	-0.77564
KCTD3	0.002266	0.33242	-0.50583
SAFB2	0.002234	0.33242	-0.43261
RTN4RL1	0.002147	0.32707	-1.05282
NXPH2	0.002113	0.326578	-0.9586
C18orf63	0.00207	0.322986	-0.4601
FZD7	0.001968	0.319688	-0.82894
CHD6	0.001843	0.319688	-0.50065
LOC10013(	0.00181	0.319688	-0.53896
RN5S195	0.001758	0.319552	-1.53164
MAST4-IT1	0.001713	0.318708	-0.44613
ROBO2	0.001568	0.311619	-0.82276
SNORD114	0.001568	0.311619	-0.55422
CWF19L2	0.001513	0.307365	-0.53907
RYR2	0.001493	0.307365	-0.36667
NOXRED1	0.00148	0.307365	-0.4584
SASS6	0.001473	0.307365	-0.76053
HIST2H2BF	0.001436	0.306227	-0.65056
PABPC5	0.001428	0.306227	-0.48647
SOS1-IT1	0.001397	0.306227	-0.71501
ANKRD5	0.001318	0.306227	-0.90626
1-Dec	0.001274	0.306227	-0.50249
YTHDC1	0.001139	0.301131	-0.47129

SLC7A5P2	0.001103	0.299719	-0.96288
LOC10050(	0.001071	0.299719	-0.80412
FP6628	0.001022	0.299719	-0.82705
OPN1MW	0.000951	0.299719	-0.63368
LUC7L3	0.000865	0.288362	-0.45031
PATE2	0.000865	0.288362	-0.36288
OR4N2	0.000862	0.288362	-0.4038
KCNG4	0.000856	0.288362	-0.53406
ELOVL2	0.000843	0.288362	-0.59038
VPS33B	0.000822	0.288362	-0.78783
GLIPR1L2	0.000786	0.288362	-1.16168
KRTAP4-7	0.000676	0.288362	-0.8547
AGTR1	0.000659	0.288362	-0.69718
RABL3	0.000657	0.288362	-0.48235
ZNF559	0.000599	0.288362	-0.74104
FBXW4P1	0.000579	0.288362	-0.32303
C1orf109	0.00053	0.288362	-0.70706
THEGL	0.000526	0.288362	-0.60641
MIR4794	0.000473	0.288362	-0.47949
FLJ38717	0.000443	0.288362	-0.62736
C1orf21	0.00044	0.288362	-0.82125
LOC40002	0.00043	0.288362	-0.89085
HOTAIR	0.000424	0.288362	-0.5754
ESPL1	0.000408	0.288362	-0.73465
KCNJ2-AS1	0.000323	0.288362	-0.77514
SOBP	0.00029	0.288362	-0.49058
EGOT	0.000237	0.272535	-1.97183
C12orf69	0.000212	0.272535	-0.92781
SHISA6	0.000202	0.272535	-0.96705
GCSHP3	0.000183	0.272535	-0.51125
GPX3	0.000169	0.272535	-1.1589
ZFP14	0.000146	0.272535	-0.86085
DCAF12L2	8.01E-05	0.248632	-1.44562
LOC10050!	2.65E-05	0.234972	-1.47811

Supplementary Table S4

Table S4 showing primer pairs (with corresponding DNA sequence) used in the study.

S. No.	Primer name	Sequence (5' -> 3')
1	18S rRNA-F	GGCCCTGTAATTGGAATGAGTC
2	18S rRNA-R	CCAAGATCCAACTACGAGCTT
3	5S rRNA-F	GGCCATACCACCCTGAACGC
4	5S rRNA-R	CAGCACCCGGTATTCCCAGG
5	HsRPS26-F	GAAAAGAAGGAACAATGGTCGTGCC
6	HsRPS26-R	CATCGAAGACGCTCGCTTCAGAA
7	MsRPS26-F	AAGAAGAAACAACGGTCGCGC
8	MsRPS26-R	CGTCGAAGACGCTTGCTTCAGATA
9	β-actin-F	AAATCTGGCACCACACCTTC
10	β-actin-R	GGGGTGTTGAAGGTCTCAAA
11	MsShroom3 Ex 2-4 1F	AGTTCCAGGAGAGAGGCTGT
12	MsShroom3 Ex 2-4 1R	CAGGCTGTGTCTCCCCAAAT
13	MsNphs1-F	GTGCCCTGAAGGACCCTACT
14	MsNphs1-R	CCTGTGGATCCCTTTGACAT

Supplemental Methods:

Cell Culture: Human podocyte cell line (Generous gift of Dr Moin Saleem (University of Bristol, UK) to Dr. He), were expanded using RPMI-1640 (1% ITS) and DMEM (GIBCO) media, respectively. Podocytes were differentiated in Collagen coated culture plates/flasks. Protein:DNA ratio assay was performed by seeding 1000-podocytes per well in Collagen-coated 96 well plates. Briefly, cells were lysed using 1X SSC buffer, for 1 hour with intermittent shaking. DNA determination was performed as described previously (1) following addition of Hoechst 33258 (Sigma) at 1µg/ml and read at excitation wavelength 360 nm, emission wavelength 460 nm using a SpectraMax M3 microplate reader. Protein concentration was determined by Bradford protein assay (Bio Rad laboratories). Protein/DNA ratios were then calculated and shown as dot plots.

Reverse transcription: RNA was extracted using TRIzol and was transcribed into cDNA using a High Capacity cDNA Reverse Transcription Kit (Applied Biosystems) with starting total RNA ~ 1000 ng.

Quantitative-PCR: Transcript expression was assayed *in vitro/in vivo* by real-time polymerase chain reaction (qPCR) (Applied Biosystems 7500). Amplification curves were analyzed using automated 7500 software platform, via the delta-delta CT method. Actin was used as endogenous control. Primers were designed for human and mouse 18S RNA, 5S RNA, RPS26, SHROOM3, Nphs1 & Actin are listed in (Table S4).

Western Blotting: Cells were lysed with a buffer containing 25 mM Tris-HCl pH 7.4, 150 mM NaCl, 1 mM EDTA, 1% NP-40 and 5% glycerol, a protease inhibitor mixture and tyrosine and serine/threonine phosphorylation and phosphatase inhibitors. Lysates were subjected to immunoblot analysis using polyclonal SHROOM3 Rabbit antibody (#SAB3500818, Sigma), Fyn Rabbit polyclonal (#4023S, Cell Signaling), Actin Mouse monoclonal antibody ; (# A5441, Sigma), phospho-mTOR (Ser2448) Rabbit monoclonal antibody [EPR426(2)] (#ab109268, Abcam), mTOR (Ser2448) Rabbit polyclonal antibody (#ab2732, Abcam), phospho-AMPK (Thr172) Rabbit polyclonal antibody (#2535, Cell Signaling), AMPK Rabbit polyclonal antibody (#2532, Cell Signaling), phospho-eEF2 (Thr56) Rabbit polyclonal antibody (#2331, Cell Signaling), eEF2 Rabbit polyclonal antibody (#2332, Cell Signaling), turboGFP Mouse monoclonal antibody, clone OTI2H8 (#TA150041, CiteAb), Phospho-ULK1 (Ser555) Rabbit polyclonal Antibody (#5869, Cell Signaling), ULK1 (D8H5) Rabbit monoclonal antibody (#8054, Cell Signaling), LC3A/B Rabbit polyclonal antibody (#4108, Cell Signaling). Densitometry was performed on images of western blots using ImageJ software.

shRNA suppression studies: Human *SHROOM3* (2, 3) and *FYN* short hairpin clones (Dharmacon, inc, USA) were tested for optimal suppression in human podocytes. The selected GFP-tagged or (red fluorescent protein) RFP-tagged hairpins and respective scrambles were used to generate a mammalian VSV pseudotyped lentiviral expression construct. Lentiviral medium was used to infect human podocytes at 33°C. Cells were passaged in puromycin (2µg/ml)-RPMI 1640 for experiments after atleast 7-days differentiation at 37°C.

Immunofluorescence (Podocytes): Stably infected Shroom3-, Fyn-shRNA and Scramble-1 & 2 podocytes were plated in 12-cm wells on collagen-coated cover slips (20% rat-tail collagen, BD biosciences, San Jose CA) and allowed for differentiation for 7-10 days. Followed by formalin-fixation (4% HCHO, 0.1% TritonX100 in PBS), blocking in 3% BSA and 0.5% Triton X- 100 containing PBS. Blocked podocytes were incubated overnight with primary antibodies i.e., LC3A/B Rabbit polyclonal antibody (#4108, Cell Signaling) or LKB1 Rabbit monoclonal antibody (D60C5F10) (IHC Formulated) #13031, Cell Signaling) at a 1:100 dilution followed by 1xPBS washes and secondary antibody incubation using Alexa-Fluor 488 (Invitrogen, Life technologies, Grand Island NY) at 1:200 dilution in 3% BSA-PBS for immunofluorescence. Samples were mounted using VECTASHIELD Antifade Mounting Medium with DAPI for nuclear staining. **Immunofluorescence (Kidney Tissues):** For IF, 5-µm paraffin sections of formalin-fixed kidney-tissues were deparaffinized and processed for unmasking and antigen retrieval followed by overnight incubation with primary antibodies anti-pAMPK, anti-WT1; for fluorescence microscopy as described previously. >30 glomeruli per mouse were assayed in 40X images for pAMPK/WT-1/DAPI co-staining.

Quantitative Image Analysis: pAMPK-Immunofluorescence: Glomeruli were outlined using Zen pro (Zen 2.6 (blue edition)) software (40X images), and area of pAMPK-staining were measured for signal intensity and expressed as average signal intensity total area/glomerulus. All glomeruli in the biopsy tissues of the mice were included (ranging from 24-58, average of 44 glomeruli per mouse).

Murine Shroom3 knockdown model: Tetracycline-responsive, shRNAmir-mediated Shroom3 knockdown mouse strain based on tested shRNA guide sequences was developed with Mirimus Inc, NY. In this mouse model, Shroom3 knockdown was induced with tetracyclines by a specific shRNA hairpin in a MIR-30 backbone, selected after application of unique design algorithms(4), and followed by *in vitro* validation in murine kidney cell lines has been reported before by our group(2, 3). In the double-transgenic Chicken beta-Actin promoter (CAGS) rtTA/Nphs1-rtTA; Shroom3 RNAi mice, shRNAmir-mediated knockdown and GFP production were driven by a universal promoter and, inducible by Doxycycline feeding (DOX). Nphs1-rTTA/Shroom3 RNAi mice mice were generated for podocyte-specific shRNA expression (generous gift from Dr Miner to Dr He(5)). CAGS/Nphs1-rtTA animals were backcrossed into BALBc background. Male mice (~8-week-old) were DOX-fed for 6 weeks (600mg/g DOX chow), Non transgenic DOX-fed littermates were used as controls. Kidney tissues were collected for histology, EM, immunofluorescence (snap frozen for IF) RNA, protein. Glomeruli were extracted using DYNA-bead perfusion.

***For Ageing studies**,* Control and Shroom3-KD mice were aged >1 year, before DOX feeding for 6 weeks was initiated.

AMPK inhibition studies were performed in control vs Shroom3-KD mice using Compound C. Adult mice (after 4 weeks of Dox feeding to induce Shroom3 knockdown) mice were injected intraperitoneally with 4 doses every 24 hours at 20mg/kg Compound C in mineral oil (vehicle) and observed till week 8 before sacrifice (15, 16).

AMPK activation studies were performed in BALBc mice - FSGS model (5/6 nephrectomy) using PF-06409577. Mice were orally dosed at 50mg/kg PF-06409577 in Vehicle (0.5% methyl cellulose/0.1% Tween-80 before surgery-1 removing 2/3rd of the left kidney, followed by 100mg/kg PF-06409577 after surgery-2 and continued for 3 times a week for 6-wks (14). For histology evaluation, 85.91±26.5 glomeruli were utilized.

Metformin protocol for aged mice: Metformin (dose= 300 mg/kg body weight per day(6)) in drinking water was added at week-2 of DOX feeding (after onset of proteinuria based on prior data), and continued till sacrifice.

In vivo sample assays: BUN measurement was performed on serum samples using Quantichrom™ assay (Bio Assay, Inc) as described (2).

Serum creatinine was assayed using a handheld analyzer, single-use, biosensor i.e. StatSensor® assay (Novabimedical inc, MA; Measurement range= 0.3-12.0 mg/dL)). The assay was adapted for use in mice by 1:1 dilution of sample with the provided creatinine standard (1mg/dl).

[Actual creatinine concentration (mg/dl) = 2 x Observed creatinine concentration – 1]

Uninephrectomy/5/6th Nephrectomy: For Uninephrectomy studies, Eight-week old Doxycyline fed male control vs Shroom3-KD/ Podocyte-Shroom3-KD mice were subjected to Unilateral nephrectomy, nephrectomized kidneys (Nx-Kidney) were saved. Mice were sacrificed either 1 or 2 weeks after surgery to obtain the residual kidney (Rx-Kidney) for various morphometry, histology studies.

For 5/6 nephrectomy, 7-8-week-old mice were used. During the first surgery, 2/3rd of the left kidney was removed, by exposing it through an angular incision in the Left aspect of erector spinae muscle (left lumbar region). The upper and lower poles for 2/6 reduction of total renal mass was ligated and cut. The secured remnant 1/3rd left kidney was placed inside the muscle layer, the muscle layer and skin were then separately closed using 4-0 vicryl. After 7 days, angular Incision was made in Right aspect of erector spinae muscle (right lumbar region) exposing right kidney. 4-0 Vicryl suture was used to run a loop around the vascular pose of the kidney (hilum) (including renal artery, vein and ureter). Kidney was held with atraumatic forceps and a clean cut made lateral to the secured loop; and removed for processing and data. The secured hilum was placed inside the muscle layer. The muscle layer and skin were separately closed using 4-0 vicryl (7).

Drug treatments: AMPK inhibition studies were performed in control vs Shroom3-KD mice using Compound C. 7-8 week old mice (following 4 weeks of Dox feeding to induce Shroom3 knockdown) mice were injected intraperitoneally with 4 doses every 24 hours at 20mg/kg Compound C in mineral oil (vehicle) and observed till week 8 before sacrifice (8, 9).

AMPK activation studies were performed in BALB-c mice - FSGS model (5/6 nephrectomy) using PF-06409577. 7-8 week old mice were orally dosed at 50mg/kg PF-06409577 in Vehicle (0.5% methyl cellulose/0.1% Tween-80 before the first stage of surgery removing 2/3rd of the left kidney, followed by 100mg/kg PF-06409577 once one right kidney was removed and continued for 3 times a week for 42 days before sacrifice (7).

in vitro autophagy inhibition studies were performed in Scramble-1 and SHROOM3 shRNA podocytes using Bafilomycin A1. Bafilomycin A1 (10) was added to the culture media at Day7 of differentiation at a concentration

of 100nM for 24 hours, following which the podocytes were fixed using 4% Formalin in 1xPBS and followed for immunofluorescence study (11).

Glomerular Morphometry:

Tissue Processing: Kidneys were perfused with PBS for five minutes. One-millimeter cubes were cut from the cortex and placed in glutaraldehyde. Tissue cubes were then post-fixed with 1% osmium tetroxide, dehydrated through a series of ethanol and embedded in Polybed 812 (Electron Microscopy Sciences, Hatfield, PA).

Glomerular Volume-Weibel-Gomez Method: *Glomerular Volume-Weibel-Gomez Method:* The Weibel-Gomez method was used to measure glomerular volume (Vglom) in human NS biopsies. This method uses one PAS-stained and one Trichrome stained paraffin section from Aperio-scanned images of NS biopsies from the NEPTUNE study (12). The areas of all complete glomerular profiles present in the section were measured by planimetry (Figure S1A).

Vglom = $A^{3/2}$ x 1.38 μm^3 where A is the average glomerular tuft area and 1.38 is the shape correction factor assuming glomeruli are spheres(13). The mean-Vgloms obtained from two sections within each patient were highly correlated (Fig S1B). The PAS-stained sections were used for stereological analyses.

The mean-Vgloms obtained from two sections within each patient were highly correlated (Fig S1B). The PAS-stained image data was used for clinical analyses.

Glomerular Volume-Cavalieri Method: Glomerular volume (Vglom) was measured using the Cavalieri Principle (14). Serial 1-µm-thick epon sections were cut using an ultramicrotome and every 10th section was saved to a slide and stained with 1% toluidine blue. Using the 10x microscope objective, a map was drawn of all the glomerular profiles present in the first section. Using the map and the subsequent sections, newly appearing glomeruli were mapped and sequentially numbered. Only complete glomeruli defined as having a section before its appearance on a section and after its disappearance were used for measurements (Figure S1A). For each complete glomerulus, the 100x objective was used to image each profile from the individual glomeruli. An average of 6.9 profiles was imaged per glomerulus. Using Adobe Photoshop's layers function a grid of points (100mm apart) was superimposed over each profile and the number of grid points "falling" over a profile was counted. $V_{glom} = 10 \times (d/mag)^2 \times \sum Points\ \mu m^3$ where 10 is the distance between profiles in µm, d is the distance between grid points in µm, mag is the magnification and ∑Points is the sum of grid points "falling" on the profiles from a glomerulus (Figure S1A). An average of 11.0 glomeruli per animal was measured and an average of 1509 grid points were counted over all the glomeruli per animal.

Glomerular Component Volume: One-half of the images obtained for Cavalieri were used for estimation of the volumes of glomerular components (Fig S1B-D). For odd numbered glomeruli the odd numbered images were used and for even numbered glomeruli the even numbered images were used. A representative image used for glomerular component volume analyses is shown in Fig S1B. Glomerular profile was first defined by a minimal polygon around the glomerular profile in image. Podocyte substructure such as pseudocysts are visible on light microscopy using this technique as published earlier(15). As described previously(2, 16), we defined four glomerular components: podocyte, capillary lumen + endothelial cell, mesangium, and "other" (pseudo-color in Fig S1C). The "other" component was defined as Bowman's space, glomerular basement membrane and non-resolvable areas within the glomerular profile. The "other" data was not analyzed in this study. Using Photoshop's layers function a grid of points was superimposed over the images. The number of points falling on each component was counted (Fig S1D). The volume fraction of component X per glomerulus [V_v(Comp X/glom)] = $\sum Points_{Comp\ X} / \sum Points_{Total}\ \mu m^3/\mu m^3$ where $\sum Points_{Comp\ X}$ is the number of points "falling" on component X, $\sum Points_{Total}$ is the number of points "falling" on all four components of the glomerulus(13). An average of 169.2, 152.4, 255.6, and 78.6 points fell on podocyte, mesangium, capillary lumen + endothelial cell and "other" respectively. The volume of a Component X (CompxVglom) is calculated by multiplying the component volume fraction by the glomerular volume: CompxVglom = V_v(Comp X/glom) x Vglom μm^3.

Podocyte Number: The fractionator/disector method was used to count the number of podocytes per glomerulus (N_{podo}) (17, 18). Podocyte nuclei were surrogates for podocytes assuming only one nucleus per podocyte. For this method pairs of sections are needed. We used the 1-µm epon sections available for the Cavalieri determination of Vglom. In addition, at the time of saving sections for the Cavalieri method the section adjacent to the Cavalieri section was saved to make disector pairs. At the time of imaging for the Cavalieri method the adjacent section was also imaged. Using Photoshop's pencil tool all the podocyte nuclei profiles were mark on all the sections. Then looking at the pairs of images next to each other the number of podocyte nuclei profiles

from nuclei present in one section but not in the other were counted. This was repeated for each disector pair from a glomerulus. An average of 6.9 disector pairs per glomerulus was available. $N_{podo} = \sum Q^{-} \times 10$, where $\sum Q^{-}$ is the number of nuclei profiles from podocytes present in one section but not present in the other section of a disector pair and 10 is the reciprocal of the fraction of the glomerulus sampled. An average of 128.8 Q^{-} were counted per kidney.
Foot process width quantification: For quantitative ultrastructural analysis of the glomerulus by transmission EM (TEM), the number of podocyte foot processes present in each micrograph was divided by the total length of GBM; to calculate the mean density of podocyte foot processes. Capillary loops in atleast three separate glomeruli of each animal, adding up to a mean of 1088±339.6 foot processes were counted over an average of 467.9±113.7 mm of the basement membrane. The thickness in each image was measured using ImageJ software.

Data Collection: Publicly available human microarray dataset for all kidney diseases [focal segmental glomerulosclerosis (FSGS), membranous nephropathy (MN), minimal change disease (MCD) and others] was downloaded from GEO (GSE68127). We collected high-throughput transcriptome data for 99 disease and control samples. Each dataset manually selected the samples with clinical information. For each study, we grouped the samples with the clinical and phenotypic information reported by the corresponding original studies. Then, for the raw microarray data, we performed quality assessment, and all the microarray platform data were re-annotated to the most recent NCBI Entrez Gene Identifiers (Gene IDs) by AILUN (http://ailun.ucsf.edu). All the expression values were base-two log-transformed and normalized by quantile–quantile normalization.

DEG identification: Principle Component Analysis (PCA) was first performed to assess the sample correlations using the expression data of all the genes. The LIMMA test was applied for analysis of data. A specific gene was considered differentially expressed if the P value given by these methods was ≤0.05.

Pathway Network, Generation, and Analyses: The DEGs for microarray and sequencing in kidney diseases compared with normal were identified by meta-analysis. Then, DEGs for the tubules and glomeruli were compared to obtain the unique and overlapping DEGs. We used two methods to perform gene enrichment analysis. DEGs with a fold change cutoff of ≥1.5 were used INGENUITY IPA (www.ingenuity.com/products/ipa) and Enrichr (https://amp.pharm.mssm.edu/Enrichr/) for GO and pathways.

Statistical analysis: De-identified clinical and demographic information was obtained for the NS morphometry cohort and linked to morphometry measurements using unique-IDs. ***For human data,*** univariate comparisons of clinical factors and demographics between NS categories were done using ANOVA (Kruskal Wallis for corresponding nonparametric analysis with post-test Dunn's test) for continuous variables, and Chi-Square for proportions. Spearman correlation coefficient was used to compare Vgloms in two random sections within the same patient. Cox proportional hazard models were used for multivariable survival associations, including clinic-demographics identified as significantly different in uni-variable analyses. NEPTUNE determined outcomes of End stage renal failure, eGFR decline ≥ 40% from baseline, or a composite of these events were evaluated as outcomes. Time from biopsy to event was utilized. ***For in vitro and in vivo experiments,*** unpaired t test was used to analyze data between two groups. *in vitro* experiments were repeated multiple times to obtain standard deviations, and representative experiments are shown. Univariate comparisons of continuous variables were done using unpaired t-test (Mann-Whitney test for corresponding non-parametric analysis). When >2 groups were compared, ANOVA or Kruskal Wallis (for non-parametric analyses with post-test Dunn's test) was used. Statistical significance was considered with two-tailed $P<0.05$. ***Software:*** Graphpad Prism Version 9 (Graphpad, LaJolla, CA) and SPSS version 24 (IBM, NY) were used for analyses.

Study approval: Institutional IACUC approved protocol was available for all mouse experiments performed according to humane endpoints. The human data in this work was obtained via a NEPTUNE anciliary study to examine morphometric, genomic and signaling changes in MCD, FSGS and all NS. The anciliary study was approved in 2018 and renewed in 2020 to permit analyses. Institutional IRB approval (Exemption 4) was obtained to examine de-identified NEPTUNE data.

Supplemental References:

1. Rao J, and Otto WR. Fluorimetric DNA assay for cell growth estimation. *Anal Biochem.* 1992;207(1):186-92.
2. Wei C, Banu K, Garzon F, Basgen JM, Philippe N, Yi Z, et al. SHROOM3-FYN Interaction Regulates Nephrin Phosphorylation and Affects Albuminuria in Allografts. *J Am Soc Nephrol.* 2018;29(11):2641-57.
3. Menon MC, Chuang PY, Li Z, Wei C, Zhang W, Luan Y, et al. Intronic locus determines SHROOM3 expression and potentiates renal allograft fibrosis. *J Clin Invest.* 2015;125(1):208-21.
4. Premsrirut PK, Dow LE, Kim SY, Camiolo M, Malone CD, Miething C, et al. A rapid and scalable system for studying gene function in mice using conditional RNA interference. *Cell.* 2011;145(1):145-58.
5. Lin X, Suh JH, Go G, and Miner JH. Feasibility of repairing glomerular basement membrane defects in Alport syndrome. *J Am Soc Nephrol.* 2014;25(4):687-92.
6. Bachmanov AA, Reed DR, Beauchamp GK, and Tordoff MG. Food intake, water intake, and drinking spout side preference of 28 mouse strains. *Behav Genet.* 2002;32(6):435-43.
7. Kir S, Komaba H, Garcia AP, Economopoulos KP, Liu W, Lanske B, et al. PTH/PTHrP Receptor Mediates Cachexia in Models of Kidney Failure and Cancer. *Cell Metab.* 2016;23(2):315-23.
8. McCullough LD, Zeng Z, Li H, Landree LE, McFadden J, and Ronnett GV. Pharmacological inhibition of AMP-activated protein kinase provides neuroprotection in stroke. *J Biol Chem.* 2005;280(21):20493-502.
9. Abdulrahman RM, Boon MR, Sips HC, Guigas B, Rensen PC, Smit JW, et al. Impact of Metformin and compound C on NIS expression and iodine uptake in vitro and in vivo: a role for CRE in AMPK modulation of thyroid function. *Thyroid.* 2014;24(1):78-87.
10. Riediger F, Quack I, Qadri F, Hartleben B, Park JK, Potthoff SA, et al. Prorenin receptor is essential for podocyte autophagy and survival. *J Am Soc Nephrol.* 2011;22(12):2193-202.
11. Bhaskar Das DPW. LKB1-AMPK ACTIVATORS FOR THERAPEUTIC USE IN POLYCYSTIC KIDNEY DISEASE. https://pubchem.ncbi.nlm.nih.gov/patent/US2017334892#section=Patent-Submission-Date.
12. Lemley KV, Bagnasco SM, Nast CC, Barisoni L, Conway CM, Hewitt SM, et al. Morphometry Predicts Early GFR Change in Primary Proteinuric Glomerulopathies: A Longitudinal Cohort Study Using Generalized Estimating Equations. *PLoS One.* 2016;11(6):e0157148.
13. ER W. *Stereological Methods*. London: Academic Press; 1979:44-5.
14. Gundersen HJ, and Jensen EB. The efficiency of systematic sampling in stereology and its prediction. *J Microsc.* 1987;147(Pt 3):229-63.
15. Zhou Y, Castonguay P, Sidhom EH, Clark AR, Dvela-Levitt M, Kim S, et al. A small-molecule inhibitor of TRPC5 ion channels suppresses progressive kidney disease in animal models. *Science.* 2017;358(6368):1332-6.
16. Basgen JM, and Sobin C. Early chronic low-level lead exposure produces glomerular hypertrophy in young C57BL/6J mice. *Toxicol Lett.* 2014;225(1):48-56.
17. Sterio DC. The unbiased estimation of number and sizes of arbitrary particles using the disector. *J Microsc.* 1984;134(Pt 2):127-36.
18. Bai XY, and Basgen JM. Podocyte number in the maturing rat kidney. *Am J Nephrol.* 2011;33(1):91-6.

Supplemental acknowledgements:

We acknowledge Tina Mainieri and Jonathan Troost, Dr Holzman and members of the NEPTUNE anciliary study committee, and the NEPTUNE consortium investigators.

Members of the Nephrotic Syndrome Study Network (NEPTUNE)

NEPTUNE Enrolling Centers

Cleveland Clinic, Cleveland, OH: K Dell*, J Sedor**, M Schachere#, J Negrey#

Children's Hospital, Los Angeles, CA: K Lemley*, B Silesky#

Children's Mercy Hospital, Kansas City, MO: T Srivastava*, A Garrett#

Cohen Children's Hospital, New Hyde Park, NY: C Sethna*, K Laurent #

Columbia University, New York, NY: P Canetta*, A Pradhan#

Emory University, Atlanta, GA: L Greenbaum*, C Wang**, C Kang#

Harbor-University of California Los Angeles Medical Center: S Adler*, J LaPage#

John H. Stroger Jr. Hospital of Cook County, Chicago, IL: A Athavale*, M Itteera

Johns Hopkins Medicine, Baltimore, MD: M Atkinson*, T Dell#

Mayo Clinic, Rochester, MN: F Fervenza*, M Hogan**, J Lieske*, V Chernitskiy#

Montefiore Medical Center, Bronx, NY: F Kaskel*, M Ross*, P Flynn#

NIDDK Intramural, Bethesda MD: J Kopp*, J Blake#

New York University Medical Center, New York, NY: H Trachtman*, O Zhdanova**, F Modersitzki#, S Vento#

Stanford University, Stanford, CA: R Lafayette*, K Mehta#

Temple University, Philadelphia, PA: C Gadegbeku*, S Quinn-Boyle#

University Health Network Toronto: M Hladunewich**, H Reich**, P Ling#, M Romano#

University of Miami, Miami, FL: A Fornoni*, C Bidot#

University of Michigan, Ann Arbor, MI: M Kretzler*, D Gipson*, A Williams#, C Klida#

University of North Carolina, Chapel Hill, NC: V Derebail*, K Gibson*, E Cole#, J Ormond-Foster#

University of Pennsylvania, Philadelphia, PA: L Holzman[*], K Meyers**, K Kallem[#], A Swenson[#]

University of Texas Southwestern, Dallas, TX: K Sambandam[*], Z Wang[#], M Rogers[#]

University of Washington, Seattle, WA: A Jefferson[*], S Hingorani**, K Tuttle**[§], M Bray [#], E Pao[#], A Cooper[#§]

Wake Forest University Baptist Health, Winston-Salem, NC: JJ Lin*, Stefanie Baker[#]

Data Analysis and Coordinating Center: M Kretzler*, L Barisoni**, J Bixler, H Desmond, S Eddy, D Fermin, C Gadegbeku**, B Gillespie**, D Gipson**, L Holzman**, V Kurtz, M Larkina, S Li, S Li, CC Lienczewski, J Liu, T Mainieri, L Mariani**, M Sampson**, J Sedor**, A Smith, A Williams, J Zee.

Digital Pathology Committee: Carmen Avila-Casado (University Health Network, Toronto), Serena Bagnasco (Johns Hopkins University), Joseph Gaut (Washington University in St Louis), Stephen Hewitt (National Cancer Institute), Jeff Hodgin (University of Michigan), Kevin Lemley (Children's Hospital of Los Angeles), Laura Mariani (University of Michigan), Matthew Palmer (University of Pennsylvania), Avi Rosenberg (Johns Hopkins University), Virginie Royal (University of Montreal), David Thomas (University of Miami), Jarcy Zee (University of Pennsylvania) Co-Chairs: Laura Barisoni (Duke University) and Cynthia Nast (Cedar Sinai).

*Principal Investigator; **Co-investigator; [#]Study Coordinator

[§]Providence Medical Research Center, Spokane, WA
